# DNA dynamics and computation based on toehold-free strand displacement

**DOI:** 10.1038/s41467-021-25270-7

**Published:** 2021-08-17

**Authors:** Hong Kang, Tong Lin, Xiaojin Xu, Qing-Shan Jia, Richard Lakerveld, Bryan Wei

**Affiliations:** 1 School of Life Sciences, Tsinghua University-Peking University Center for Life Sciences, Center for Synthetic and Systems Biology, Tsinghua University, Beijing, China; 2 Department of Chemical and Biological Engineering, The Hong Kong University of Science and Technology, Clear Water Bay, Hong Kong, China; 3 Center for Intelligent and Networked Systems, Department of Automation, Beijing National Research Center for Information Science and Technology (BNRist), Tsinghua University, Beijing, China

**Keywords:** Molecular self-assembly, DNA computing, DNA nanostructures

## Abstract

We present a simple and effective scheme of a dynamic switch for DNA nanostructures. Under such a framework of toehold-free strand displacement, blocking strands at an excess amount are applied to displace the complementation of specific segments of paired duplexes. The functional mechanism of the scheme is illustrated by modelling the base pairing kinetics of competing strands on a target strand. Simulation reveals the unique properties of toehold-free strand displacement in equilibrium control, which can be leveraged for information processing. Based on the controllable dynamics in the binding of preformed DNA nanostructures, a multi-input-multi-output (MIMO) Boolean function is controlled by the presence of the blockers. In conclusion, we implement two MIMO Boolean functions (one with 4-bit input and 2-bit output, and the other with 16-bit input and 8-bit output) to showcase the controllable dynamics.

## Abstract (web summary)

Synthetic DNA constructs can to used to recognise and respond to input signals. Here the authors present complex DNA nanostructures with toehold-free strand displacement for generation of ON/OFF switches and Boolean gates.

## Introduction

In self-assembled DNA nanostructures and especially ones from the scaffolded origami method, designated base pairing can be specified to result in a deterministic structure^1–4^. However, in most complex DNA nanostructures, a pre-specified segment of base pairing is always static once complemented. On the other hand, toehold mediated strand displacement has enabled many DNA dynamic systems^5–7^, including switchable devices^8, 9^, walkers^10–15^, triggered amplification^16, 17^, and circuits^12, 18, 19^. Especially, there are a few examples of dynamic megadalton DNA nanostructures in which the paired segments with toeholds make the systems switchable from one state to another^20–25^.

**Fig. 1:**
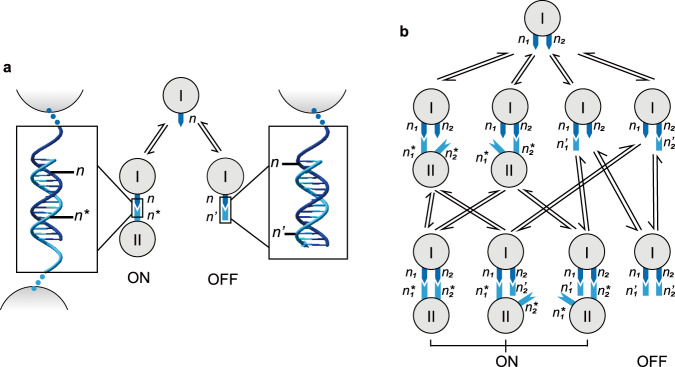
Schematic diagrams of controllable dynamics of DNA nanostructures based on toehold-free strand displacement. Tree maps of binding reactions of (**a**) one species of blocker to displace one species of pre-specified segment (nn*) or (**b**) two species of blockers to displace two species of pre-specified segments (*n*_1_*n*_1_*, *n*_2_*n*_2_*).

Instead of toehold mediated strand displacement, we demonstrate that controlled dynamics can be achieved by the simpler implementation of toehold-free strand displacement^26^. Under such a scheme, when a single-stranded DNA (ssDNA) blocker (e.g., *n*’) fully complementary to a certain segment of paired duplex (e.g., nn*) is presented at an excess amount, it can displace the specific segment by pairing competition (e.g., *nn*’) (Fig. 1a). The blocking can be applied to an arbitrary set of segments of paired duplexes (e.g., NN*, *N* = {*n*_1_, *n*_2_, …, *n*_*i*_}; N* = {*n*_1_*, *n*_2_*, …, *n*_i_*}). When a counterpart set of ssDNA blockers (e.g., *N*’ = {*n*_1_’, *n*_2_’, …, *n*_*i*_’}) is presented at an excess amount, the original pairing scheme will be outcompeted and displaced (Fig. 1b). In other words, when the original pairing scheme of NN* without strand displacement is defined as an ON state, the pairing after strand displacement (*NN*’) can be defined as an OFF state.

In general, our experimental and simulated results exhibit the functional mechanism of toehold-free strand displacement. The controllable dynamics has been applied in a number of DNA nanostructure systems for ON/OFF switch and Boolean functions. We believe the simple and effective implementation could enable advanced computation with DNA nanostructures^27^.

## Results

### Proof-of-concept system to illustrate the basic dynamics

To demonstrate the dynamics based on toehold-free strand displacement, we first designed a simple proof-of-concept system. In our polyacrylamide gel electrophoresis (PAGE) assays involving fluorescently labeled strands, we prove the strand displacement indeed happens without toeholds. The toehold-free design results in a reversible strand displacement reaction, which is fundamentally different from the conventional toehold-mediated design.

**Fig. 2:**
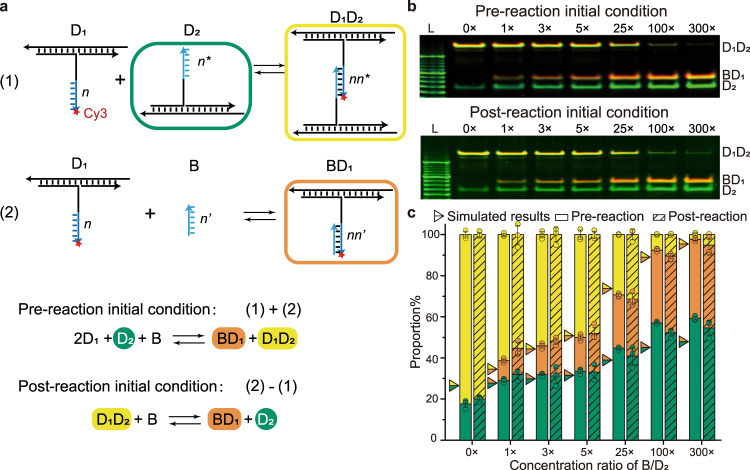
Proof-of-concept system of toehold-free strand displacement. **a** Schematic diagram of reaction. Reaction (1): binding of D_1_ and blocker. Reaction (2): binding of D_1_ and D_2_. Details in Supplementary Note 1 and Supplementary Figs. 1–14. Green square highlights D_2_, yellow square highlights D_1_D_2_ and orange square highlight BD_1_. The same color theme applies to all panels across this figure. **b** PAGE results of pre-reaction and post-reaction initial condition at 40 °C with concentration gradient of blocker from 0 × to 300 × (1 × = 125 nM). Three independent experiments were repeated (detailed statistics in Supplementary Figs. 3 and 5). **c** Statistical analysis from PAGE results (mean ± SD, *N* = 3) and the corresponding simulations. Unshaded bars: pre-reaction initial condition; shaded bars: post-reaction initial condition; triangles: simulated results from reaction equilibrium model.

In this proof-of-concept system, we designed two duplexes (D_1_ and D_2_) to be connected by a single pair of binding partners (segment *n* in D_1_ and segment n* in D_2_). We also introduced a blocker (B, strand *n*’) into this system (Fig. 2a), which can dissociate the two duplexes if toehold-free displacement is kinetically favorable. With fluorophore (e.g., Cy3) modified on the 3′ end of segment *n*, the different molecule species (e.g., D_1_, D_1_D_2_, and BD_1_) can be identified after native PAGE assay. Our results with PAGE purified strands demonstrate that the fraction of D_1_D_2_ complex decreases with increasing blocker concentration and that, therefore, the single-pair system indeed functions according to a mechanism involving toehold-free strand displacement. To better understand such mechanism, we developed a probabilistic model based on master equations to simulate the kinetics of the base pairing of D_1_ with D_2_ or a blocker (Supplementary Note 2)^28–31^. This model simulates the kinetic pathways leading to displacement and can help elucidate whether the observed experimental trends can be explained mechanistically from DNA hybridization kinetics at the base-pair level. The model simulations predict that the probability of observing D_1_ in a D_1_D_2_ complex reduces with the increasing concentration of blocker according to the equilibrium probability distribution. The predicted time to reach equilibrium from the model simulations of around a hundred seconds matches the experimental fluorescence data at least by order of magnitude (Supplementary Figs. 19 and 20), suggesting that 1 h is sufficient for the toehold-free displacement system to reach equilibrium. Similar experimental results are obtained for blocking in a pre-reaction or post-reaction initial condition (Fig. 2b), which further shows the reversibility of the system. The toehold-free system behaves qualitatively different from a conventional toehold-mediated system. Model simulations predict that the probability of displacement by a blocker essentially vanishes when the length of blocker is minimally reduced, which has been confirmed by an experiment with a blocker that is 1-nt shorter than the binding partner of D_2_ (Supplementary Figs. 18 and 21). Any displacement system with such 1-nt shorter blocker will be less reversible. The probabilistic model based on master equations can broadly explain the observed reversibility of the toehold-free displacement system and its sensitivity with respect to the blocker concentration by simulating the displacement pathways with hybridization kinetics at the base-pair level. However, it cannot describe the equilibrium concentrations of all species in the system. Therefore, we also developed a deterministic reaction pathway model to understand the impact of design parameters on the species concentrations at equilibrium. The Δ*G* of each pairing or blocking reaction in this model was fitted to experimental data, which reveals that the Δ*G* of each reaction is higher compared to the one predicted from the nearest neighbor thermodynamics model^32^ (Supplementary Notes 3–6). Such higher Δ*G* can potentially be attributed to a lower Δ*S* of the pairing reaction of the binding partner of D_1_ compared to standard duplex formation. Furthermore, the Δ*G* for the formation of the D_1_D_2_ complex is lower than the Δ*G* for the formation of BD_1_, which suggests that the D_1_D_2_ complex is more stable at the tested temperatures. The estimated Δ*G* for the formation of BD_1_ decreases with increasing temperature, which suggests that the OFF state of such system is favored at a higher temperature (Supplementary Fig. 14). This decreasing Δ*G* with temperature would suggest that the blocking process is entropy-driven (Δ*S* > 0), but could also be caused by other temperature-dependent effects that are not captured by the model.

A dual-pair system with two pairs of binding partners was also designed and implemented (Supplementary Fig. 8).The product distribution as function of the blocker concentration is different for the case of such dual-pair system (Supplementary Fig. 22) compared to the single-pair system discussed earlier (Supplementary Fig. 21). A significant dissociation of the D_1_D_2_ complex in case of the dual-pair system only occurs when the concentration of the blockers exceeds a certain threshold, whereas the fraction of the D_1_D_2_ complex in the product distribution of the single-pair system decreases more gradually with the blocker concentration. The reaction pathway model of the single-pair system can be extended to simulate the dual-pair system after fitting to experimental data to understand whether this qualitative difference in behavior between the single-pair and dual-pair system can be explained from basic reaction equilibria. Such simulations indeed also predict the existence of a blocker threshold for significant dissociation in case of the dual-pair system (Supplementary Note 6), which suggests that such threshold is an inherent characteristic of a multi-pair system. Intermediate structures can exist for the dual-pair system. By labeling two component strands with different fluorophores (i.e., Cy3 on D_1_ and Cy5 on blocker B_2_), we managed to identify these intermediate structures of D_1_D_2_ (i.e., D_1_B_1_D_2_, D_1_B_2_D_2_, in which one of the binding partners of D_1_D_2_ is blocked). Simulations of the reaction equilibrium model of the dual-pair system revealed that the fraction of these intermediate structures of D_1_D_2_ is <1% (Supplementary Note 6), which explains why these intermediates could not be separated by PAGE. The low fraction of intermediate structures and the blocker threshold imply cooperativity between the binding partners of multi-pair systems. Finally, the estimated Δ*G* suggested that the D_1_D_2_ complex is energetically less stable than the blocked duplex, which is different from the single-pair system. One reason for such difference could be an entropy penalty that arises from the internal loop formation of the D_1_D_2_ complex with dual binding partners^33^. The cooperativity associated to the dual-pair system implies an improved stability of switches based on multi-pair designs compared to a single-pair design. Therefore, we believe the design of DNA nanostructures with multiple binding partners will benefit the MIMO Boolean functions. Furthermore, the experimental and simulation results show that the higher temperature stabilizes the blocked state better compared to the lower temperature, which allows the switch to be triggered at lower blocker concentrations and is therefore preferred.

The length of binding partner was set as 16-nt for the proof-of-concept systems and also for most systems in the rest of the article. Systems of 8-nt binding partners (e.g., *n* and n*) were also tested, but the 8 bp binding was too weak and transient to sustain the association of D_1_ and D_2_ (Supplementary Figs. 7 and 13).

### Dynamic ON/OFF switch of a dual-unit 2D origami system

To employ the concept in complex DNA nanostructures, we then applied the control to the binding of two preformed 2D origami structures. With different numbers of pairing partners, we successfully demonstrated the controllable ON/OFF switch based on toehold-free strand displacement.

**Fig. 3:**
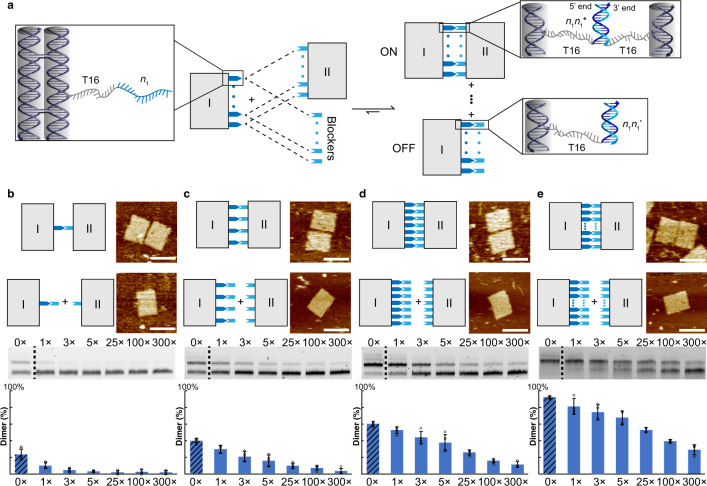
ON/OFF switch of a dual-unit 2D DNA nanostructure system. **a** Schematic diagram of ON/OFF switch. The binding of two 24H × 26T origami units is resulted from the complementation of as many as 15 pairs of connecting staples, which can be displaced by as many as 15 blockers. Strand level of details shown in insets for different dynamic states. **b**–**e** Results with one pair of sticky ends and one blocker (**b**), four pairs of sticky ends and four blockers (**c**), seven pairs of sticky ends and seven blockers (**d**) and all 15 pairs of sticky ends and 15 blockers (**e**). Top, diagrams and corresponding AFM images (scale bars: 100 nm). Middle, agarose gel electrophoresis results without blocker (0×) and of different blocker to sticky end concentration ratios (1× to 300×); dimers as upper bands and monomers as lower bands. Bottom, yield analysis of dimer formation based on gel results for different blocker concentrations in blue histograms (yield without blocker (0×) in shaded histogram). Four independent experiments were repeated and the detailed statistics (e.g., mean and SD) in Supplementary Figs. 24–27.

The first example is to control the binding of two preformed 2D origami structures. Each of the two origami rectangles (24 helices × 26 helical turns from close-packed monolayer helices with twist correction^34, 35^) was designed with sticky ends on one of the long edges for dimerization (Fig. 3a). These two rectangles units (I and II) shared the same core staples but varied by connection staples. A typical connection staple was designed with three segments, a common staple segment complementary to the scaffold, a linker segment (16-nt poly-T), and a sticky end segment (16-nt). Unit I was specified with sticky end set *N* (*N* ⊆ *N*_15_ = {*n*_1_, *n*_2_, …, *n*_*i*_, …, *n*_15_}) and unit II with sticky end set N* (N* ⊆ *N*_15_* = {*n*_1_*, *n*_2_*, …, *n*_i_*, …, *n*_15_*}). The index of *N*_15_ represents the total number of sticky ends, *i* represents the position of a sticky end in *N*_15_. Such a complementation scheme corresponded to the binding of units I and II as an ON state. A counterpart set of blockers *N*’ (*N*’ ⊆ *N*_15_’ = {*n*_1_’, *n*_2_’, …, *n*_*i*_’, …, *n*_15_’}), fully complementary to the sticky end set *N* respectively (*NN*’), was designed to displace the pairing of NN* and switch OFF the binding (Fig. 3a).

When the blocker set of only one species (*N*_1_’ = {*n*_8_’}) was applied at excess amount to the system of preformed units I and II with only one sticky end pair (*N*_1_ = {*n*_8_}; *N*_1_* = {*n*_8_*}), the binding of the two units was significantly reduced when compared to that without blockers as a control. As shown in agarose gel electrophoresis assay, monomer and dimer products ran as separate bands and fluorescent intensities of monomer and dimer bands were used in the analysis of ON/OFF switch efficiency (Fig. 3b, middle row). When product bands were excised out from the gel and eluted samples of purification were subjected to atomic force microscopy (AFM) imaging, purified dimer structures were shown on AFM images (Fig. 3b, top right). Not surprisingly, binding efficiency increased when the number of sticky ends increased from one pair to four pairs (*N*_4_ = {*n*_2_, *n*_6_, *n*_10_, *n*_14_}; *N*_4_* = {*n*_2_*, *n*_6_*, *n*_10_*, *n*_14_*}), seven pairs (*N*_7_ = {*n*_2_, *n*_4_, *n*_6_, *n*_8_, *n*_10_, *n*_12_, *n*_14_}; *N*_7_* = {*n*_2_*, *n*_4_*, *n*_6_*, *n*_8_*, *n*_10_*, *n*_12_*, *n*_14_*}), and all 15 pairs (*N*_15_, *N*_15_*) (Fig. 3c-e, top left). An increasingly higher concentration of blockers was necessary to compensate for the kinetic favor of neighboring sticky ends.

To ensure the components react under the scheme of toehold-free strand displacement, a control experiment (Supplementary Fig. 29) with PAGE purified strands serving as sticky ends (seven pairs of sticky end segments locate at 3′ sticky end of the strands respectively) was performed. The results show a similar trend as the counterpart in Fig. 3d, which further confirms that our dynamic systems are toehold-free. Moreover, 8-nt sticky ends were also tested. Dimerization was presented in absence of blockers, but the yield was lower than samples with 16-nt sticky ends. Binding competition from blockers were not available even when blockers were applied in high concentration (Supplementary Figs. 34 and 35), which was presumably due to weak binding of individual 8-nt sticky ends.

### Boolean functions based on dual-unit and quadruple-unit 3D origami systems

Basic dual-unit and more complex quadruple-unit 3D origami systems then were designed under the scheme of toehold-free strand displacement. With blockers serving as controllers which switch ON/OFF a certain binding by toehold-free strand displacement, a 4-bit input/2-bit output and a 16-bit input/8-bit output Boolean functions are then implemented based on the reliable dynamic switch.

**Fig. 4:**
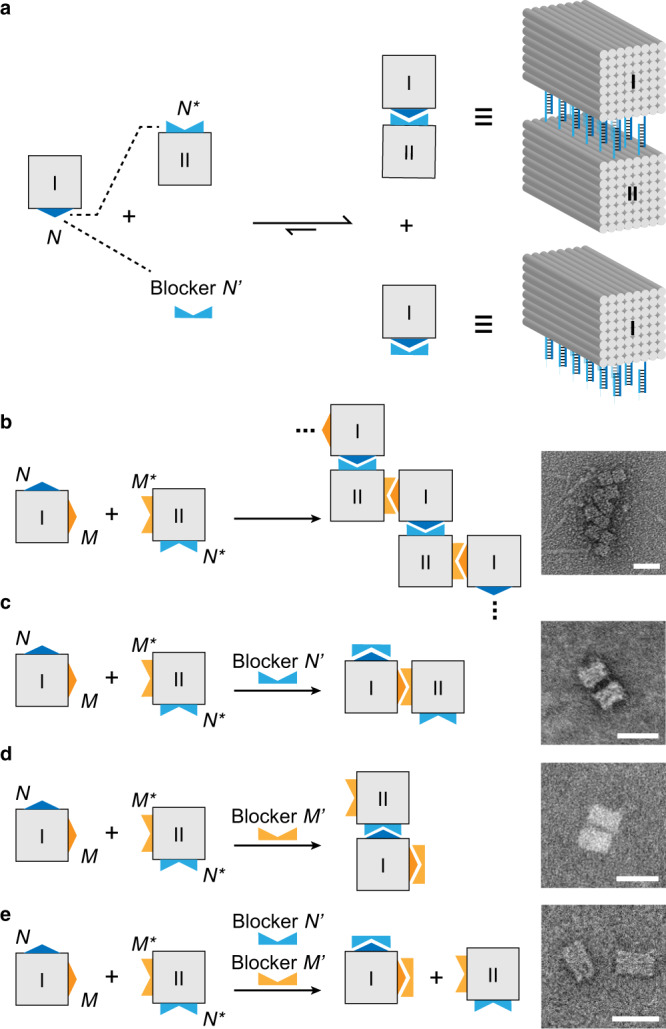
Boolean functions implemented on a dual-unit 3D DNA nanostructure system. **a** Schematic diagram of ON/OFF binding switch of two cuboid origami units (8H × 8H × 10T): the binding of cuboids I and II in the absence of blockers (as ON) and the displacement in the presence of blockers (as OFF). **b**–**e** Boolean function of 4-bit input and 2-bit output. 4-bit input comprises system state of 2 bits (NN* and MM*) of two units and controller of 2 bits (blockers *N*’ and *M*’). 2-bit output is read out as the resulted shapes (multimer, dimer or monomer) under TEM. Scale bars: 50 nm.

In a dual-unit system, two basic units of 8H × 8H × 10T cuboids shared the same core staples but varied by connection staples. Twelve connection staples with peripheral sticky ends were arranged along with two of the eight helices for a particular side face of the cuboid (Supplementary Fig. 36). According to a certain complementation scheme, a specific ‘sticky face’ of unit I pairs with its complementary ‘sticky face’ of a matching unit II, and pairing was subjected to displacement at the presence of the corresponding set of blockers (Fig. 4a). When blockers were not available, binding of units I and II was presented upon mixing as an ON state (e.g., sticky face *N* of unit I pairs with sticky face N* of unit II; Supplementary Figs. 37 and 38). When the cuboids were mixed with blockers at the excess concentration, on the other hand, the binding was displaced as an OFF state (e.g., sticky face *N* of unit I pairs with blocker set *N*’; Supplementary Figs. 37 and 39). ON/OFF switch (1-bit output) of the system in which the complementation of one face pair (1-bit input) to be controlled by one set of blockers (1-bit input) can be viewed as a Boolean function of 2-bit input and 1-bit output (Supplementary Table 5).

Similarly, when two sets of face pairs in this dual-unit system and the two corresponding sets of blockers are available, a MIMO Boolean function with 4-bit input and 2-bit output can be implemented (Fig. 4b–e and Supplementary Table 6). Notably, it can be viewed as a composite of two Boolean functions of 2-bit input and 1-bit output. Among the 4-bit input in this dual-unit system, the first 2 bits define the system states, representing whether *N* and *M* have been blocked by *N*’ and *M*’ before binding reaction, respectively (Fig. 4b–e); and the rest 2 bits serve as the controllers, representing whether blockers *N*’ and *M*’ are presented at excess amount during the binding reaction. The 2-bit output is the new system state after the control operation. In our implementation, 2-bit initial system states were fixed as *N* not blocked by *N*’ and *M* not blocked by *M*’ and the 2-bit controllers were variables. This means that among the 4-bit input, the first 2 bits were kept as constant in our experimental setup, and the rest 2 bits were subject to combinatorial specifications. The computation led to 4 (2^0^•2^2^) types of output: the absence of both *N*’ and *M*’ resulted in the pairing of both face pairs and corresponding formation of zig-zag shaped multimers (Fig. 4b); the presence of either *N*’ or *M*’ resulted in the blocking of either sticky face pair while leaving the remaining pair complemented and the corresponding dimer formation (Fig. 4c and d); the presence of both *N*’ and *M*’ resulted in a total blocking of the two sticky face pairs and the corresponding monomer formation (Fig. 4e). The resulted shapes reflected the new system states and served as reliable output of the Boolean functions. The specific output of computation was characterized in agarose gel electrophoresis assay (Supplementary Fig. 40), in which monomer and dimer showed as discrete bands and multimer showed as ladder-like bands of incremental mobility. Morphologies of the monomer, dimer and zig-zag shaped multimer products under transmission electron microscopy (TEM) further confirmed the results of desired computation (Fig. 4b–e, right panels; Supplementary Figs. 41–44).

**Fig. 5:**
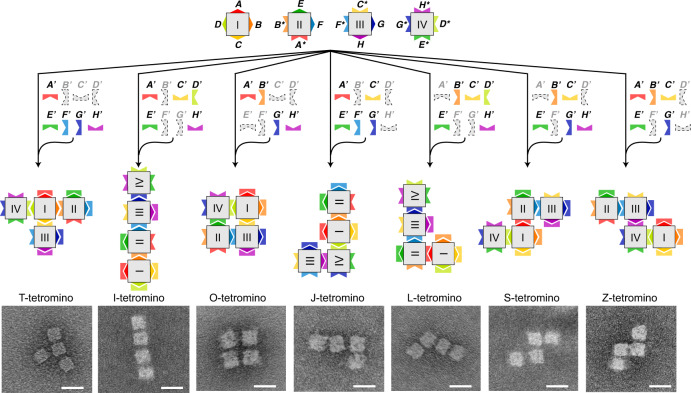
Tetromino assembly based on a quadruple-unit system. There are four origami cuboid units (8H × 8H × 10T) in the system (I, II, III, and IV), and each unit with four sticky faces to bind with sticky faces of the matching units (convex and concave in a matching color depict a certain face pair). The sets of blockers in the presence (blockers in use highlighted in respective colors while blockers not in use faded in gray) react with the four basic units to result in the formation of one of the seven tetrominoes, T-, I-, O-, J-, L-, S-, and Z-tetrominoes. Scale bars: 25 nm.

With four information-bearing sticky faces for each 3D cuboid origami unit, we then extended the concept in a quadruple-unit system, which involved four basic units of 8H × 8H × 10T origami cuboids (I, II, III, and IV). Four sticky faces were designed for each unit and sixteen sticky faces in total (eight pairs of sticky faces: AA*, BB*, CC*, …, and HH*) for the quadruple-unit system. Accordingly, eight sets of blockers (*A*’, *B*’, *C*’, …, and *H*’) were designed to displace the corresponding sticky face pairs, respectively. A MIMO Boolean function with 16-bit input and 8-bit output was implemented accordingly. Among the 16-bit input, the first 8 bits define the system states, representing whether each of the eight independent sticky face pairs has been blocked before binding reaction, respectively; the rest 8 bits serve as the controllers, representing whether the eight blockers are presented at an excess amount during the binding reaction, respectively. The 8-bit output is the new system state after the control operation. In our implementation, the initial system states before the binding reaction were fixed as none of the eight pairs of sticky face being blocked. Theoretically, there are 256 (2^0^·2^8^) output combinations in total, and 69 of them correspond to finite shapes (simulated results in Supplementary Fig. 63). Especially, 44 output shapes are tetramers (Supplementary Table 8). Experimentally, the presence of the chosen four or five sets of blockers (as controllers), with the four basic origami units resulted in the formation of one of the seven tetrominoes (T-, I-, O-, J-, L-, S-, and Z-tetrominoes, seven tetramer shapes according to the Tetris game) (Fig. 5). For example, four species of purified cuboid units were mixed chosen sets of blockers at an excess amount (blocker sets *A*’, *E*’, *F*’, *G*’ and *H*’), which led to the blocking of five specific face pairs and the pairing of the remaining three, resulting in the formation of a T shape tetromino (Fig. 5, leftmost column). There are more than one combination sets of blockers to result in a certain tetromino shape, but we only performed experiments of a particular combination for a certain shape. Similarly, six other tetrominoes were generated with the same four basic units but varying sets of blockers, respectively. The successful formations of all seven tetrominoes were verified by gel assay and TEM imaging. Tetramer bands corresponded to the desired tetrominoes in agarose gel electrophoresis results (Supplementary Figs. 45 and 46), and the desired morphologies were observed when the purified samples were subjected to TEM (Fig. 5; Supplementary Figs. 47–53). Moreover, the mirrored shapes (J piece against L piece, and S piece against Z piece) were also specifically identified by adding an extra blocked cuboid to the resulted shapes (Supplementary Fig. 54).

Besides the one-pot reaction pathway, hierarchical reaction pathways can be designed and performed for the same Boolean function. For the J-tetromino as an example (Supplementary Figs. 60–62), alternative pathways of two or three hierarchies of iterations were designed with fewer input and output bits at each round of computation to result in the same outcome of the original Boolean function (Supplementary Note 14), though the overall efficiency might be compromised (Supplementary Fig. 60). Since an output shape can be viewed as a new system state of a certain Boolean function, the sequence information embedded at the shape boundaries from a certain round of Boolean function can be used in successive rounds. In other words, the function implemented on the dynamic system can be extended in an iterative way.

## Discussion

The dynamic switch in this study is reversible, which we predicted with reaction model simulations and validated with experiments showing that different initial states led to identical final states. Simulations demonstrate that the reversibility largely vanishes when introducing short toeholds for displacement (Supplementary Figs. 18 and 21). Because of the reversibility, the equilibrium composition can be engineered by simple concentration adjustment of substrates and products to drive reaction directionality. Influx of fuel strands and generation of waste products, which drives the cycling of toehold-mediated strand displacement, are not necessary in our reversible systems. In most of the implementations, ON or OFF states are switched from a pre-reaction (i.e., pre-computation) state in which reactions between binding components are not available. The switch can also be operated from an ON state to an OFF state (Supplementary Note 8) or from OFF to ON (Supplementary Figs. 54–59). Theoretically, the final state is only determined by the ratio between the origami units and the blockers at a certain temperature, but not the initial state. However, an elevated temperature or elongated reaction time may be necessary for a full ON to OFF or OFF to ON switch in a more complex origami system.

Concentration and temperature adjustments are useful kinetic handles to implement the toehold-free strand displacement for reconfiguration of DNA nanostructures. Changes to the solution composition, such as the concentrations of ions, and denaturing and crowding agents, can also be applied to adjust the corresponding energetics and kinetics. Taken together, fine tuning would result in precise control of the desired reaction pathways.

Distinguishable shapes readily identifiable can naturally represent specific system states at certain stages of a computation process. Therefore, shape information was adopted as output for most of the Boolean functions demonstrated in this study. Although the multi-round Boolean computation is feasible according to the results J-tetromino from different reaction pathways, implementation of shape information (boundary sequence information) in general Boolean computation is beyond the scope of this study. At the same time, each origami unit has four programable sticky faces (four side faces of the origami cuboid) to be arbitrary complementary to designated sticky faces of other partner origami units, and this general architecture is also similar to a typical DNA tile computing system, which fits our systems under the same Wang tile theory paradigm as the other DNA tile based computation systems^36–38^. The opportunity of using our origami systems in Iterated Boolean Circuit for general Boolean computation is rather apparent, though the implementation could be non-trivial. We decide to schematically present two 6-bit input Boolean computation examples using the quadruple-unit system (e.g., the parity and the multiple of 3) without experimental demonstration (Supplementary Figs. 64–66). Since blockers can provide an extra layer of control to the systems, state-of-the-art DNA computation can in theory be pushed one step closer to general computation. For example, a specific Boolean computation could be implemented by a chosen set of blockers at the presence of a shared set of origami units for multiple computation pathways to direct the progression of the select one. (Supplementary Fig. 67) The control by blockers could even be applied on the fly for computation process that requires interventions on demand.

Similar dynamic control based on toehold-free strand displacement was also applied to a typical DNA tile system, in which the assembly of more than 300 species of tiles can be controlled by blockers of as many species (Supplementary Figs. 68–74). That is a substantial scaling up from the origami systems composed of just a few origami units. Moreover, the results of the tetromino from the hierarchical pathway indicates that a certain computing process of higher complexity can be designed in hierarchies. When DNA dynamics of a higher level of controllability and reliability in conjunction with scalability and hierarchical architecture lead us to the precise execution of advanced computation, such as composition and iteration, one can imagine sophisticated computation tasks to be implemented by DNA nanostructures.

## Methods

### Structural and sequence design

Strands used in simple proof-of-concept system to explore the mechanism of toehold-free strand displacement were designed and generated through *Uniquimer* software (version 1.0)^39^. In the single-pair system, each unit contains three strands, including a 15-nt strand, a 30-nt strand, and a 15-nt strand with extended segments (16-nt poly-T linker segment and 16-nt sticky end segment *n*). The Cy3 fluorophore was modified at the 3′ end of sticky end segment *n*. In the dual-pair system, each unit contains three strands, including a 30-nt strand and two 15-nt strands with extended segments (16-nt poly-T linker segment and 16-nt sticky end fragment *n*_1_′ and *n*_2_′, respectively). The Cy3 fluorophore was modified at the 3′ end of sticky end segment *n*_1_. The Cy5 fluorophore was modified at the 3′ end of blocker *n*_2_′. DNA strands were ultra-PAGE synthesized by Sangon Biotech. 2D origami rectangle (24H × 26T) in this study was adapted from an earlier study^34^. Fifteen connection staples locate along the top boundary helix. For each connection staple, a combined segment of a linker (16-nt poly-T) and a sticky end (16-nt) were sequentially appended to a common staple segment. 3D origami cuboid^40^ (8H × 8H × 10T) was designed by *caDNAno* software (version 2.0.0)^41^. A connection staple is also composed of three segments, a common staple segment, a linker segment (12-nt poly-T), and a sticky end segment (16-nt). Connection staples located along the eight highlighted helices (details in Supplementary Fig. 36). There are six connection staples designed along each highlighted helix (i.e., 12 connection staples for a sticky face). 2D rectangle from SSTs (24H × 29T) was adapted from an earlier study^42^. It consists of 375 component SSTs. The SSTs for the 16th helix were subjected to split-up by blockers. The split-up resulted in a large piece of 16H × 29T rectangle and a small piece of 8H × 29T rectangle. DNA sequences of the origami rectangle and the SST rectangle were obtained from earlier reports^34, 42^ and DNA sequences of the origami cuboid were generated by *caDNAno* software^41^. Sequences of sticky ends and the corresponding blockers were generated by *Uniquimer*  software^39^ . DNA strands were synthesized by Bioneer Corporation and Integrated DNA Technology, Inc.

### Structural assembly

To assemble the units of simplified system, strands were mixed in a roughly equimolar concentration of 1 μM for pre-reaction initial condition, and 500 nM for post-reaction initial condition in 1 × TAE buffer (40 mM Tris, pH8.0, 20 mM acetic acid, and 1 mM EDTA) supplemented with 15 mM MgCl_2_. The samples were annealed in a thermo cycler (90 °C for 3 min and a cooling from 90 to 25 °C over a period of 1 h) before reacted with different concentration of blocker. The competition experiments were conducted under 25 °C or 40 °C for 1 h. The results were analyzed by non-denaturing PAGE. To assemble the 24H × 26T origami rectangle, 10 nM scaffold M13mp18 (New England Biolabs, Inc.), 25 nM core staples and 100 nM connection staples were mixed in 1 × TE buffer (10 mM Tris, pH7.9, 2 mM EDTA) supplemented with 15 mM MgCl_2_. The sample was annealed in a thermo cycler (85 °C for 4 min and a cooling from 85 to 25 °C over a period of 2.5 h) before native agarose gel electrophoresis. To assemble the 8H × 8H × 10T origami cuboid, 10 nM scaffold M13mp18, 50 nM core staples and 100 nM connection staples were mixed in 1 × TAE buffer (40 mM Tris, pH8.0, 20 mM acetic acid, and 1 mM EDTA) supplemented with 12.5 mM MgCl_2_. The sample was annealed in a thermo cycler (85 °C for 10 min and then 52 °C for 10 h). The sample was then purified using polyethylene glycol (PEG)^43^ or native agarose gel electrophoresis. To assemble the SST rectangle, the core component DNA strands were mixed in a roughly equimolar concentration of 150 nM (component SSTs of 16th and 17th rows at 300 nM) in 0.5 × TBE buffer (44.5 mM Tris, 44.5 mM boric acid, and 1 mM EDTA) supplemented with 15 mM MgCl_2_. The sample was annealed in a thermo cycler (65 °C for 15 min and then 45.7 °C for 17 h) before native agarose gel electrophoresis. For each structural assembly and intermolecular interaction, there’re more than three independent experiments were conducted.

### Agarose gel electrophoresis and the related purification

1% or 1.5% agarose gel was prepared in 0.5 × TBE buffer supplemented with 10 mM MgCl_2_ and pre-stained with SYBR Safe (Thermo Scientific). The annealed samples were subjected to native agarose gel electrophoresis at 90 V in an ice-water bath. Then the target gel bands were excised, carefully crushed using the flat end of a plastic pestle in a Freeze’N Squeeze column (Bio-Rad), and then directly subjected to centrifugation at 106 *g* for 2 min at 4 °C or room temperature. Samples centrifuged through the column were collected by SynGene_GeneTools software (version 4.03.05.0) and used for further analysis by AFM or TEM.

### Non-denaturing PAGE analysis

12% separating gel of non-denaturing PAGE with 4% stacking gel (Shanghai WSHT Inc.) were used to analyze the results of simplified system in 0.5 × TBE buffer. The competitive equilibrium samples were subjected to non-denaturing PAGE at 80V for 1 h and 120V for 3 h in an ice-water bath and stained with SYBR Safe. Then the non-denaturing PAGE was observed by Amersham^TM^ Typhoon^TM^ RGB Biomolecular Imager (GE Healthcare) with different excitation lights and analyzed by Amersham Typhoon Control software (version 2.0.0.6).

### Polyethylene glycol (PEG) purification

Precipitation buffer PEG 8000 solution was added to the unpurified 3D cuboid origami sample solution with an equal volume^43^. A thorough mix was then subjected to centrifugation at 16,000 *g* for 25 min at 25 °C. We removed the supernatant and dissolved the obtained pellet in 0.5 × TAE buffer supplemented with 35 mM MgCl_2_. Then the purified samples were directly used or incubated overnight at 25 °C.

### AFM imaging

AFM images were obtained using an SPM Multimode with Digital Instruments Nanoscope V controller (Vecco) and collected by NANOSCOPE ANALYSIS (Bruker Corp.; version 1.50). A 50 μL drop of solution for structure assembly and a 5 μL droplet of the sample were applied to a freshly cleaved mica surface and left for ~2 min. The images were captured under liquid tapping mode, with C-type triangular tips (resonant frequency, *f*_*0*_ = 40–75 *kHz*; spring constant, *k* *=* 0.24 *N* *m*^*−1*^) from the SNL-10 silicon nitride cantilever chip (Bruker Corporation). For each structure, the AFM image were independently repeated at least three time.

### TEM imaging

A 10 μL droplet of the purified sample (2–10 nM) was applied to a plasma-treated, carbon-coated grid (Electron Microscopy Sciences) for 4 min, and then wicked off and stained for 15 s with 4 μL of stain buffer (2% aqueous uranyl formate with 25 mM NaOH). Then stain buffer was blotted off by filter paper and left on the grid to be air-dried. Sometimes, the grid was coated with 10 μL 100 mM MgCl_2_ for 4 min, then wicked off and to be air-dried before adding a sample, especially for imaging tetrominoes. The stained samples were analyzed by FEI Tecnai Spirit with iCorr, operated at 120 kV. The images were collected by TEM Imaging & Analysis software (version 4.6.4). For each structure, the TFM image were independently repeated at least three time.

### Yield quantification by gel electrophoresis

Yields were estimated by analyzing specific bands in native agarose gel electrophoresis or PAGE. The ratio between the fluorescent intensity of a target band and that of the sum of the entire lane in were taken as estimates of the yield of structural formation. Software *ImageJ* (version 1.52a)^44^ and *ImageQuant* (GE Healthcare, version 8.1) were used to calculate the fluorescent intensity.

### Reporting summary

Further information on research design is available in the Nature Research Reporting Summary linked to this article.

## Supplementary information

Supplementary Information

Supplementary Dataset 1

Supplementary Dataset 2

Description of Additional Supplementary Files

Reporting Summary

## Data Availability

All data of this study are provided within the paper and supplementary information.
