# Supplementary material for: DNA dynamics and computation based on toehold-free strand displacement
Source: Nat Commun. 2021 Aug 17;12:4994. doi: 10.1038/s41467-021-25270-7 (PMC8371076; doi:10.1038/s41467-021-25270-7)
Supplement: Supplementary file 1 — Supplementary Information [file 41467_2021_25270_MOESM1_ESM.pdf]

Supplementary Information for
DNA dynamics and computation based on toehold-free strand displacement

Hong Kang[1], Tong Lin[1,2], Xiaojin Xu[2], Qing-Shan Jia[3,*], Richard Lakerveld[2,*], Bryan Wei[1,*]

*Correspondence to : bw@tsinghua.edu.cn (B.W.); r.lakerveld@ust.hk (R.L.); jiaqs@tsinghua.edu.cn (Q.-S. J.).

This PDF file includes:

Supplementary Figures 1 to 74
Supplementary Tables 1 to 8
Supplementary Notes 1 to 14
Supplementary References 1 to 9

Other Supplementary Information for this manuscript include the following:

Supplementary Data 1 – DNA sequences
Supplementary Data 2 – Codes for simulation models

Supplementary Figures

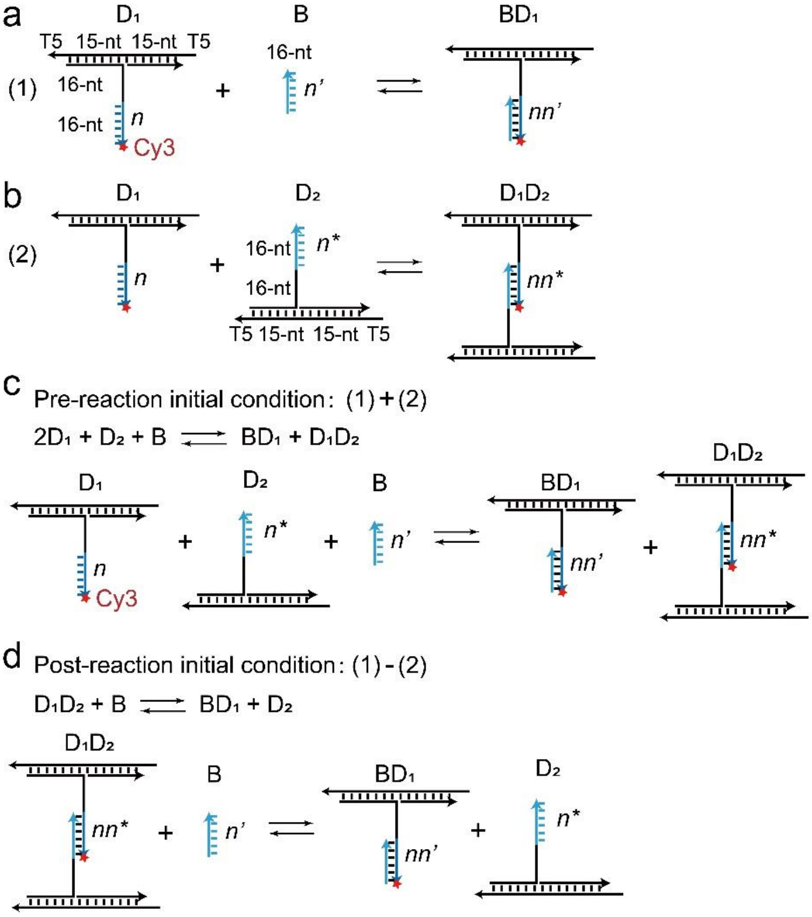

Supplementary Figure 1. Schematics of different reaction initial conditions in the single-pair system. (a) Reaction (1) is that duplex D_1 binds with corresponding blocker to form a blocked-D_1(BD_1). (b) Reaction (2) is that duplex D_1 binds with duplex D_2 to form a dimer D_1D_2. (c) Pre-reaction initial condition is reaction (1) plus reaction (2). (d) Post-reaction initial condition is reaction (1) minus reaction (2).

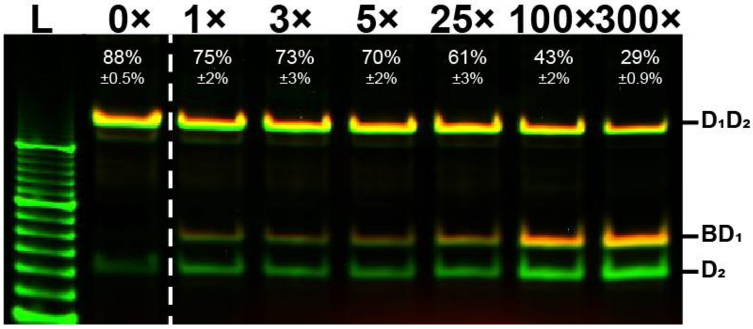

Supplementary Figure 2. Native polyacrylamide gel electrophoresis results of ON/OFF switch at pre-reaction initial condition in the single-pair system at 25℃. Lane L: 50 bp DNA ladder. Lane 0×: sample with binding switched ON; lanes n× (n=1, 3, 5, …, 300): samples with binding switched OFF. Percentages above dimer bands indicate dimer yields (mean ± SD, N=3).

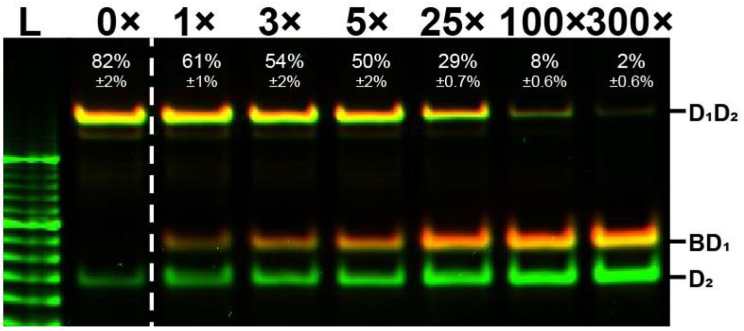

Supplementary Figure 3. Native polyacrylamide gel electrophoresis results of ON/OFF switch at pre-reaction initial condition in the single-pair system at 40℃. Lane L: 50 bp DNA ladder. Lane 0×: sample with binding switched ON; lanes n× (n=1, 3, 5, …, 300): samples with binding switched OFF. Percentages above dimer bands indicate dimer yields (mean ± SD, N=3).

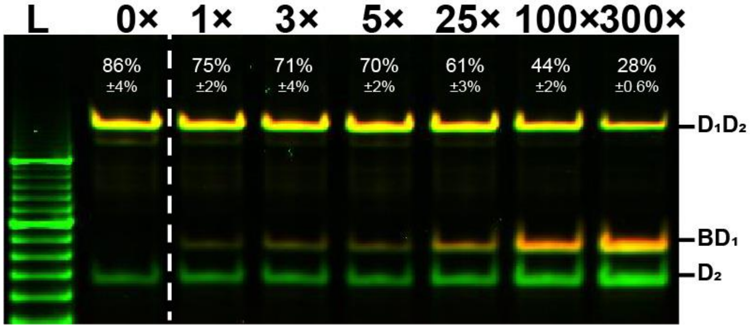

Supplementary Figure 4. Native polyacrylamide gel electrophoresis results of ON/OFF switch at post-reaction initial condition in the single-pair system at 25℃. Lane L: 50 bp DNA ladder. Lane 0×: sample with binding switched ON; lanes n× (n=1, 3, 5, …, 300): samples with binding switched OFF. Percentages above dimer bands indicate dimer yields (mean ± SD, N=3).

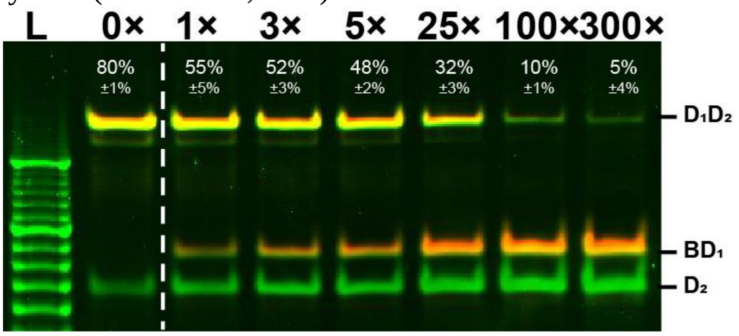

Supplementary Figure 5. Native polyacrylamide gel electrophoresis results of ON/OFF switch at post-reaction initial condition in the single-pair system at 40℃. Lane L: 50 bp DNA ladder. Lane 0×: sample with binding switched ON; lanes n× (n=1, 3, 5, …, 300): samples with binding switched OFF. Percentages above dimer bands indicate dimer yields (mean ± SD, N=3).

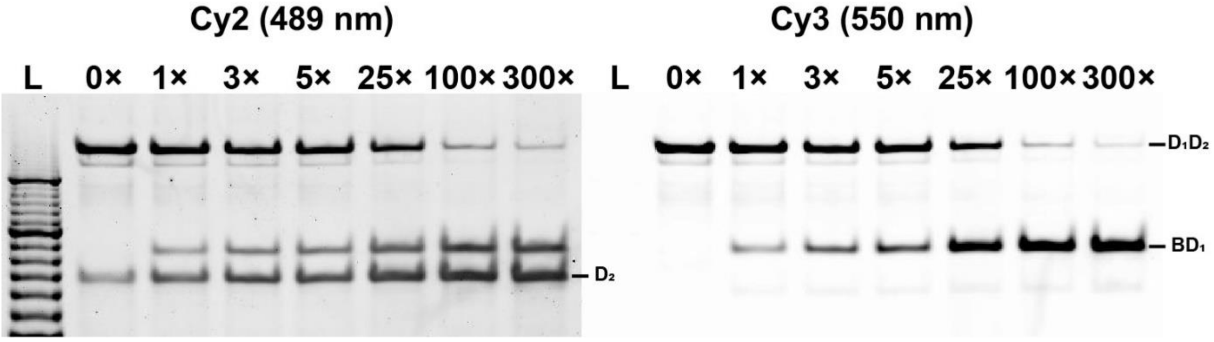

Supplementary Figure 6. Native polyacrylamide gel electrophoresis results of the ON/OFF switch excited by different lights. The fluorophore Cy3 specifically points out the position of D_1 from that of D_2. Lane L: 50 bp DNA ladder. Lane 0×: sample with binding switched ON. The D_1 is fully involved in the formation of dimer. Lanes n× (n=1, 3, 5, …, 300): samples with binding switched OFF. The D_1 is mainly reconfigured in the formation of BD_1 with the increased concentration of blocker.

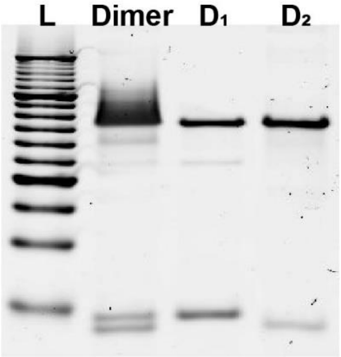

Supplementary Figure 7. Native polyacrylamide gel electrophoresis results of a single-pair system with 8-nt binding partners. The sticky end is not long enough to form dimer. Lane L: 20 bp DNA ladder.

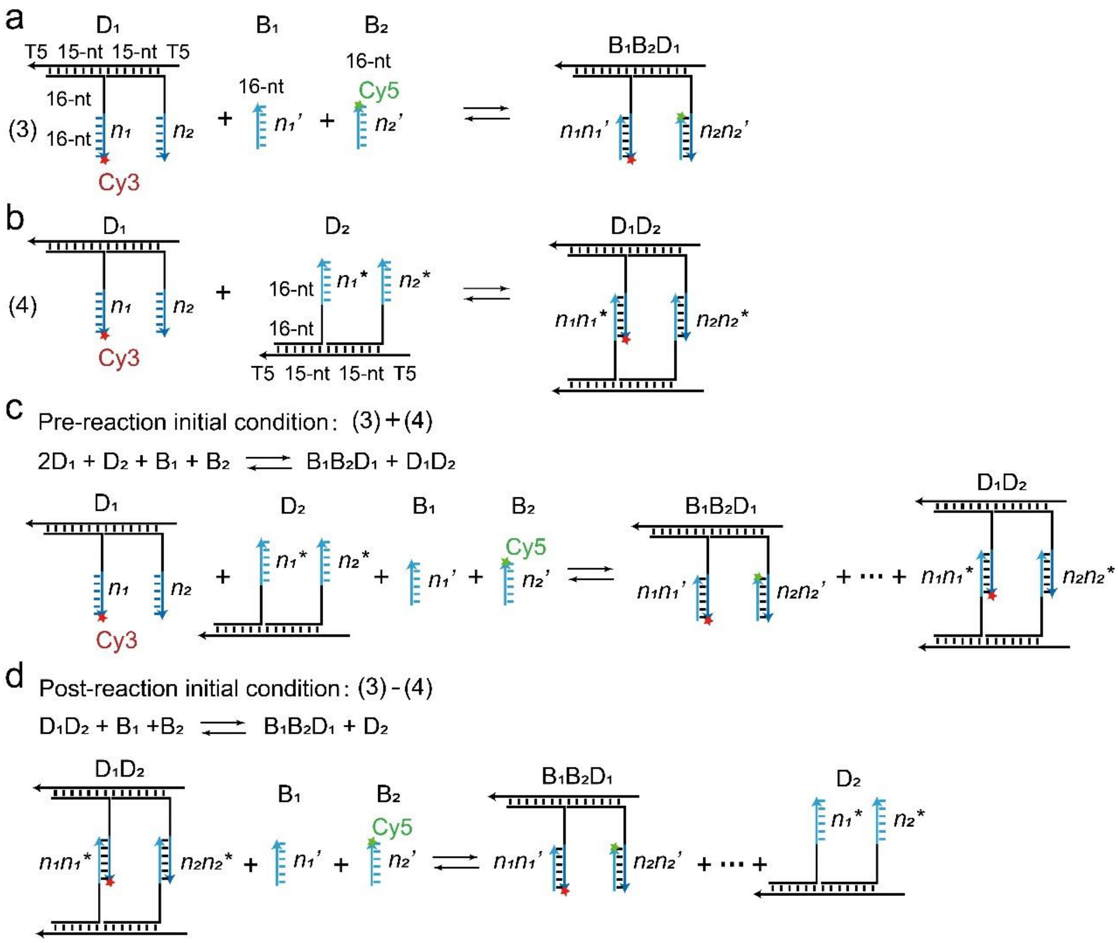

Supplementary Figure 8. Schematics of different reaction initial conditions in the dual-pair system. (a) Reaction (3) is that duplex D_1 binds with corresponding blocker to form a blocked-D_1(BD_1). (b) Reaction (4) is that duplex D_1 binds with duplex D_2 to form a dimer D_1D_2. (c) Pre-reaction initial condition is reaction (3) plus reaction (4). (d) Post-reaction initial condition is reaction (3) minus reaction (4). The intermediates are omitted but considered in simulation.

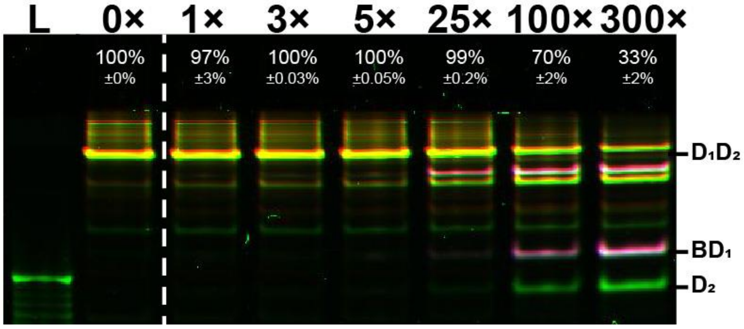

Supplementary Figure 9. Native polyacrylamide gel electrophoresis results of switch from ON to OFF at pre-reaction initial condition in the dual-pair system at 25°C. Lane L: 50 bp DNA ladder. Lane 0×: sample with binding switched ON; lanes n× (n=1, 3, 5, …, 300): samples with binding switched OFF. Percentages above dimer bands indicate dimer yields (mean ± SD, N=3).

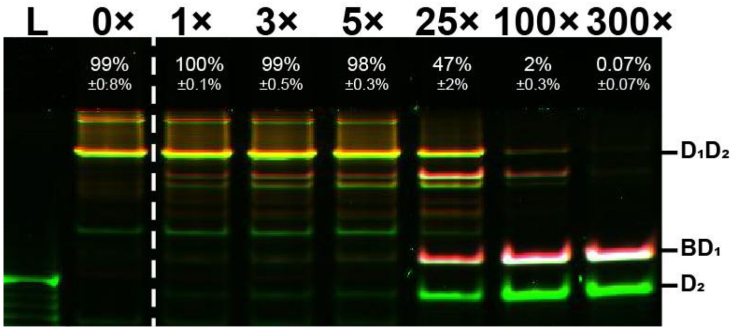

Supplementary Figure 10. Native polyacrylamide gel electrophoresis results of switch from ON to OFF at pre-reaction initial condition in the dual-pair system at 40°C. Lane L: 50 bp DNA ladder. Lane 0×: sample with binding switched ON; lanes n× (n=1, 3, 5, …, 300): samples with binding switched OFF. Percentages above dimer bands indicate dimer yields (mean ± SD, N=3).

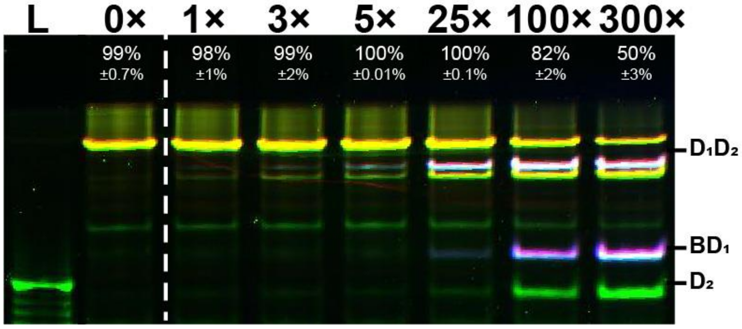

Supplementary Figure 11. Native polyacrylamide gel electrophoresis results of switch from ON to OFF at post-reaction initial condition in the dual-pair system at 25°C. Lane L: 50 bp DNA ladder. Lane 0×: sample with binding switched ON; lanes n× (n=1, 3, 5, …, 300): samples with binding switched OFF. Percentages above dimer bands indicate dimer yields (mean ± SD, N=3).

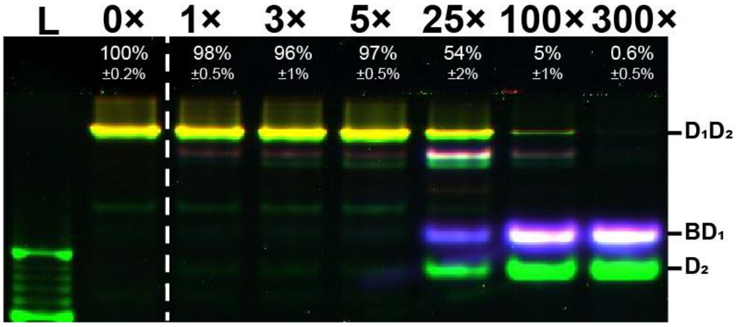

Supplementary Figure 12. Native polyacrylamide gel electrophoresis results of switch from ON to OFF at post-reaction initial condition in the dual-pair system at 40°C. Lane L: 50 bp DNA ladder. Lane 0×: sample with binding switched ON; lanes n× (n=1, 3, 5, …, 300): samples with binding switched OFF. Percentages above dimer bands indicate dimer yields (mean ± SD, N=3).

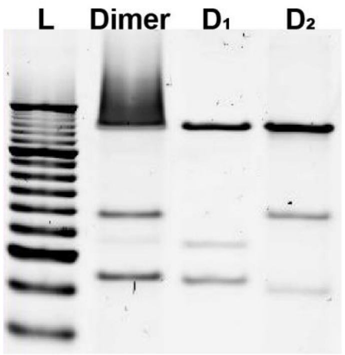

Supplementary Figure 13. Native polyacrylamide gel electrophoresis results of a dual-pairs system with 8-nt binding partners. The sticky end is not long enough to form dimer. Lane L: 20 bp DNA ladder.

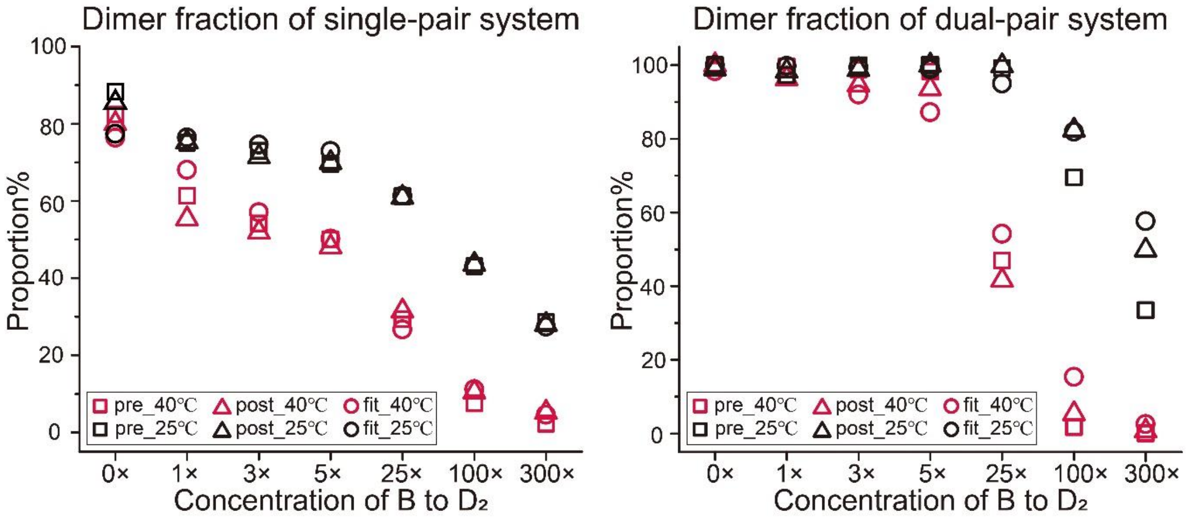

Supplementary Figure 14. Comparison results of different tendencies of dimer fraction in the single-pair system (left) and the dual-pair system (right). The black symbols mean the dimer fractions at 25℃, and red ones mean at 40℃. Different shapes mean different experimental and simulated data. The squares mean the pre-reaction data, the triangles mean the post-reaction data, and the circles mean the simulated data.

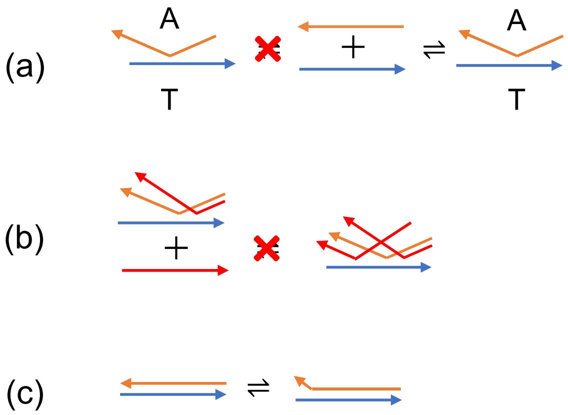

Supplementary Figure 15. Illustration of assumptions for model reduction. (a) incorrect (LHS) and correct (RHS) A-T binding; (b) neglected displacement from a third strand; (c) fraying from the edges of a double-strand domain

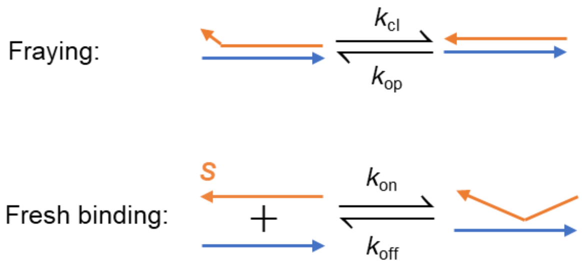

Supplementary Figure 16. Schematic of transition events. The blue arrow represents the target strand (D_1); the orange arrow represents the substrate (D_2 or B); k_{op}, k_{cl} are the kinetic constants for forward and backward reactions of end fraying; k_{on} and k_{off} are those for primary binding.

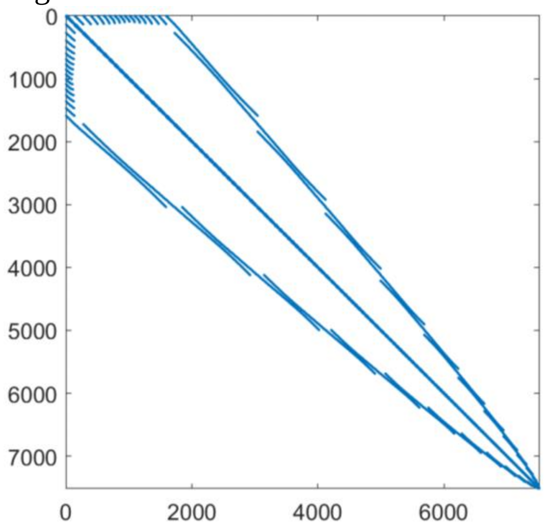

Supplementary Figure 17. Sparsity pattern of the propensity matrix in the toehold-free strand displacement of 16-nt. The row and column indices represent different states of D_1, and the dots represents a non-zero propensity for the feasible transition between two states.

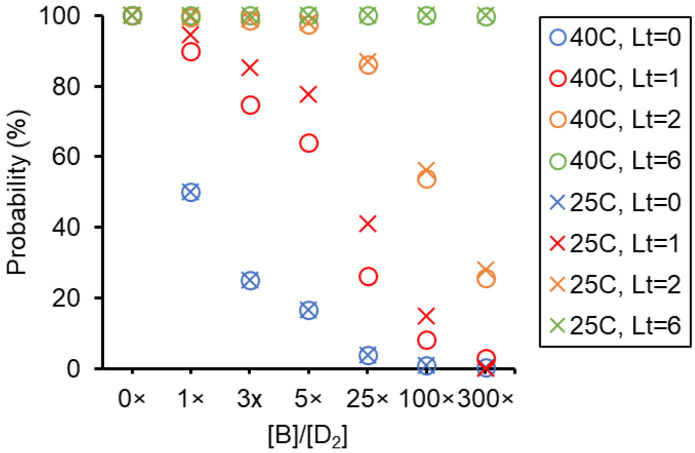

Supplementary Figure 18. The probabilities of observing the system in a dimer configuration. Different toehold lengths ($L_t \in \{0, 1, 2, 6\}$) at 3600s and two temperatures (25°C, 40°C) as a function of the concentration ratio of blocker (B) to strand D_2. The data are obtained from solving the master equations.

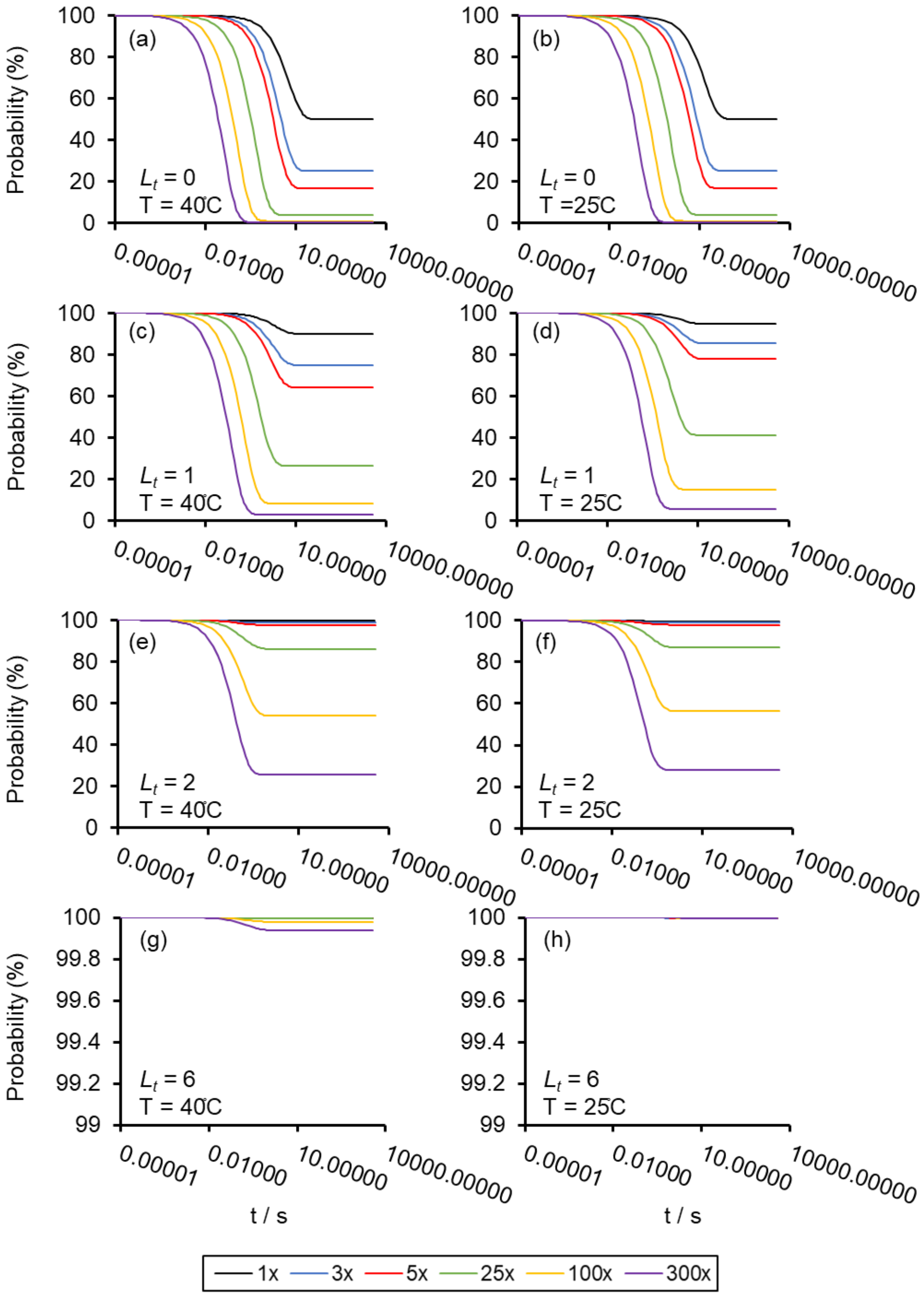

Supplementary Figure 19. Dynamic trajectories of the probabilities for observing the system in dimer state. Different lengths of the toehold domain $L_t \in \{0, 1, 2, 6\}$ were observed at two temperatures (25°C, 40°C). The data are obtained from solving the master equations. The colors of the trajectories represent different concentration ratios of blocker to D_2.

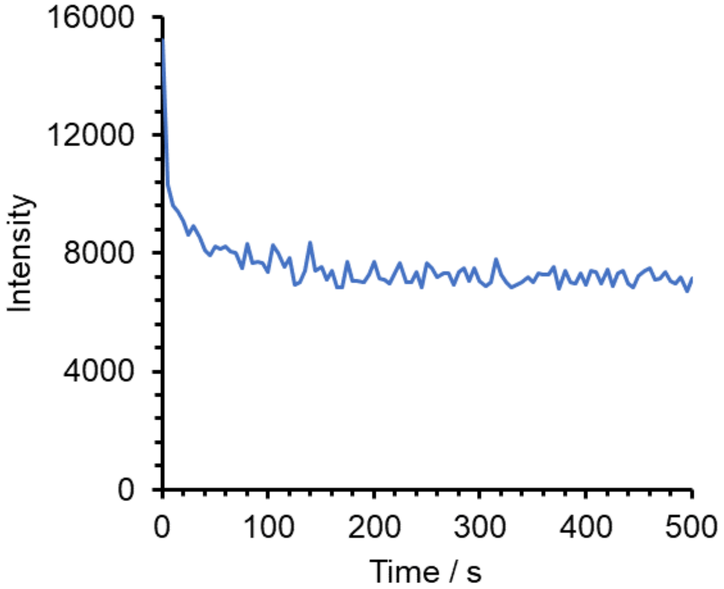

Supplementary Figure 20. Dynamic development of the fluorescence intensity for toehold-free strand displacement. A pre-assembly experiment at a concentration ratio of B to D_2 of 1× and a temperature of 40℃.

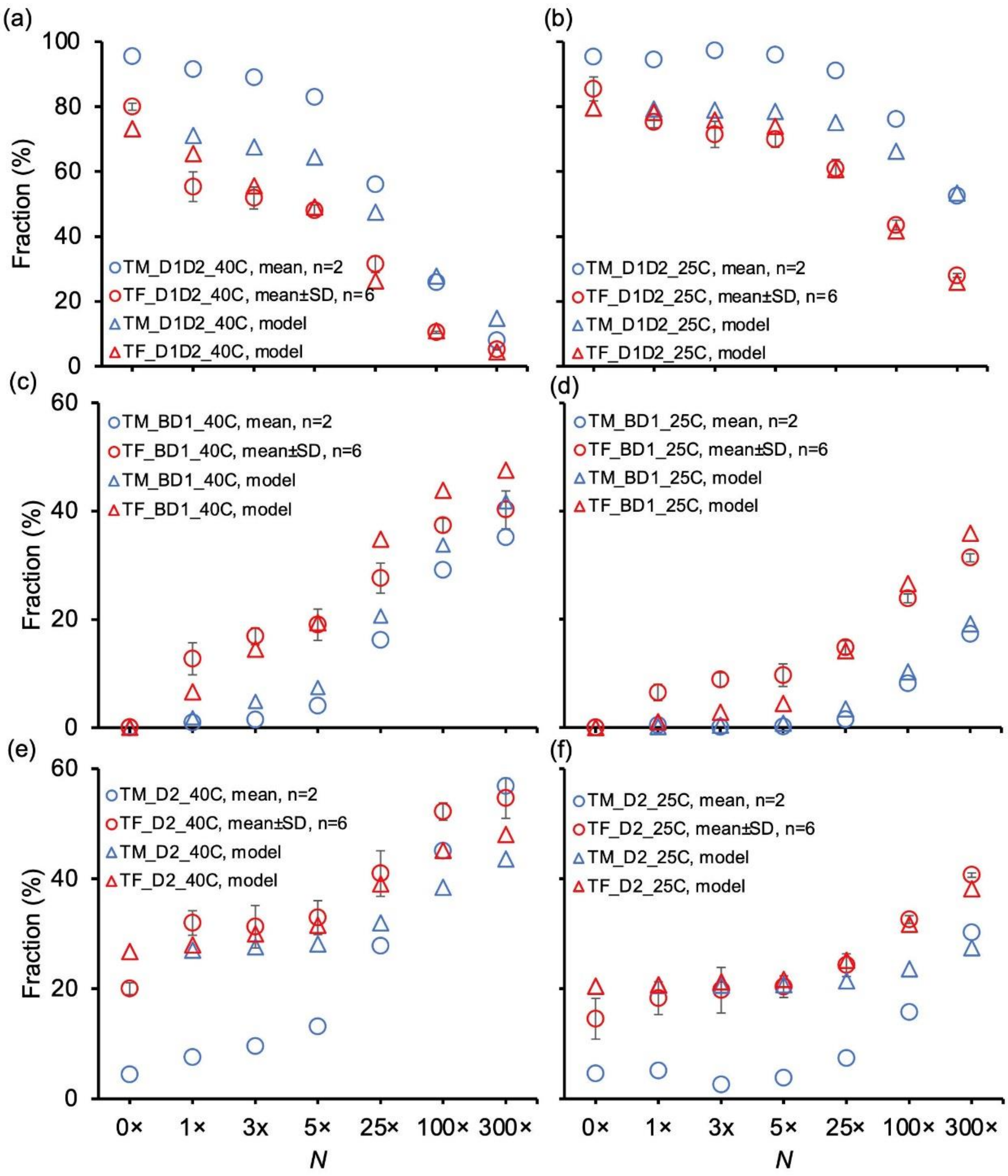

Supplementary Figure 21. Fractions of different species from experiments and model simulations at different temperatures (25℃ and 40℃). (a) and (b) for fractions of D_1D_2; (c) and (d) for fractions of B_1D_1 and B_2D_1; (e) and (f) for fractions of D_2. Indices on the horizontal axis represent the concentration ratios of blocker to species D_2. Blue and red labels represent the results of toehold-mediated (TM) and toehold-free (TF) strand displacement, respectively. The error bars represent standard deviation of measured fractions from n experiments.

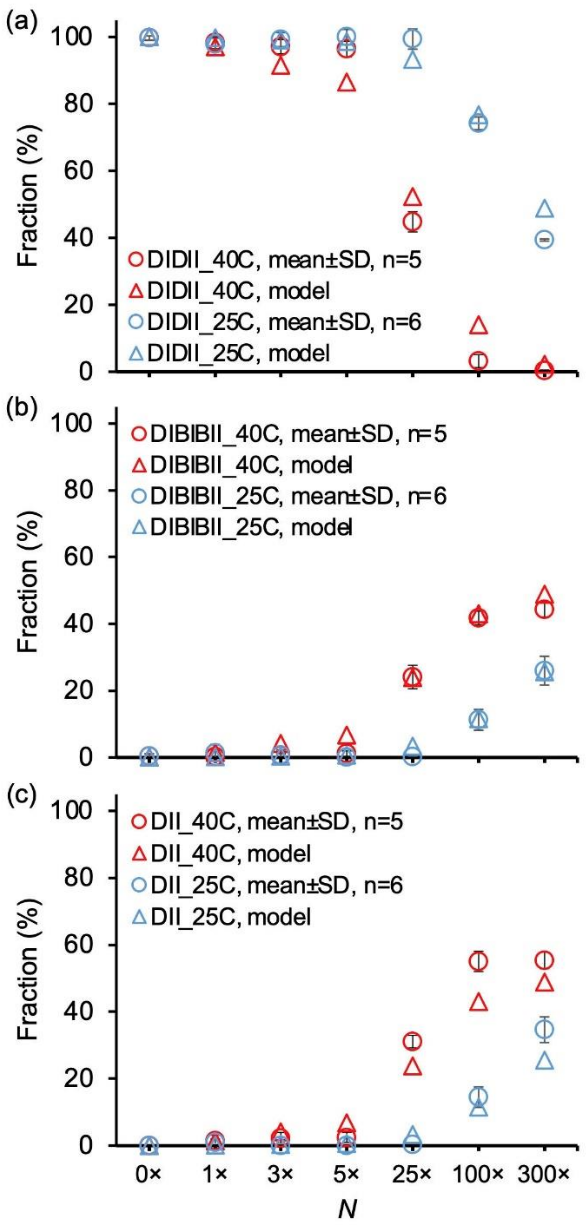

Supplementary Figure 22. Fractions of different components from experiments and modelling. (a) for fractions of $D_I D_{II}$; (b) for fractions of $D_I B_I B_{II}$; (c) for fractions of D_{II}. Indices on horizontal axis are the concentration ratios of blocker to D_{II}. Blue and red labels represent the results at 25°C and 40°C, respectively. Plots in triangles are the results from model simulations. The error bars represent standard deviation of measured fractions from n experiments.

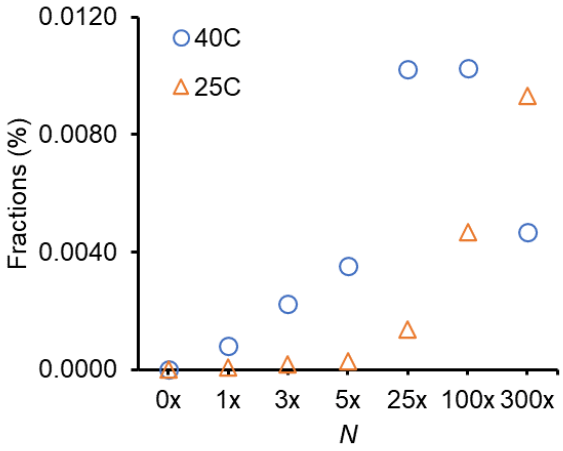

Supplementary Figure 23. Concentration fractions of intermediates ($D_IB_ID_{II}$ and $D_IB_{II}D_{II}$) to the detected species (D_ID_{II}, $D_IB_IB_{II}$ and D_{II}) at 25°C and 40°C.

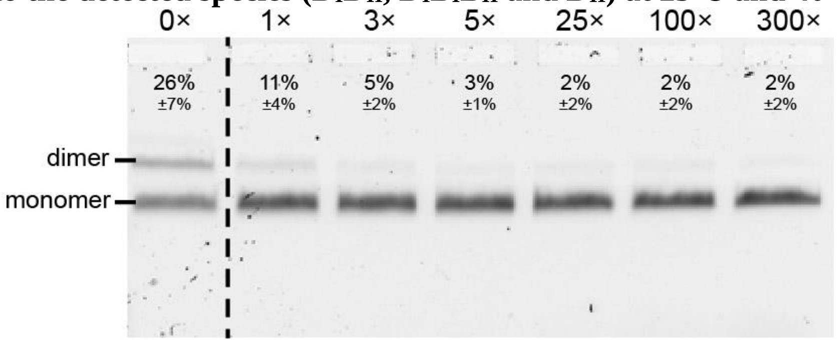

Supplementary Figure 24. Native agarose gel electrophoresis results of ON/OFF switch at pre-reaction state with one pair of sticky end and one blocker. Lane 0×: sample with binding switched ON; lanes n× (n=1, 3, 5, …, 300): samples with binding switched OFF. The incremental concentration of blocker set *N*' resulted in an increasingly higher efficiency of blocking. Percentages above dimer bands indicate dimer yields (mean ± SD, N=3).

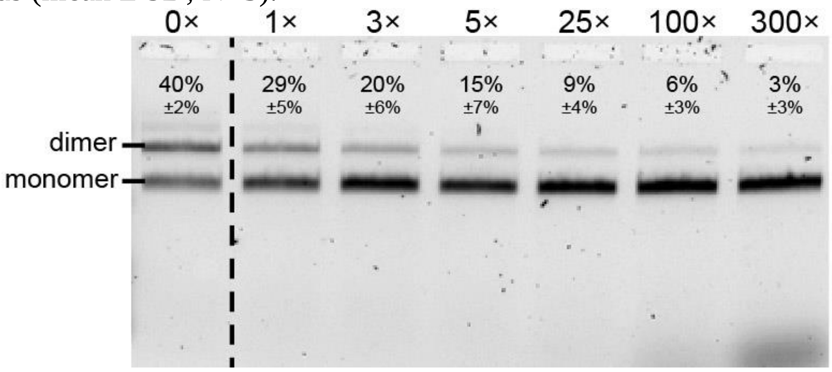

Supplementary Figure 25. Native agarose gel electrophoresis results of ON/OFF switch at pre-reaction state with four pairs of sticky ends and four blockers. Lane 0×: sample with binding switched ON; lanes n× (n=1, 3, 5, …, 300): samples with binding switched OFF. The incremental concentration of blocker set *N*' resulted in an increasingly higher efficiency of blocking. Percentages above dimer bands indicate dimer yields (mean ± SD, N=3).

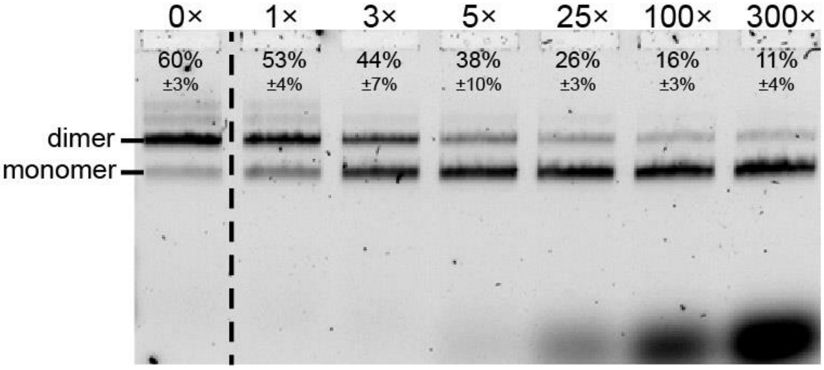

Supplementary Figure 26. Native agarose gel electrophoresis results of ON/OFF switch at pre-reaction state with seven pairs of sticky ends and seven blockers. Lane 0×: sample with binding switched ON; lanes n× (n=1, 3, 5, …, 300): samples with binding switched OFF. The incremental concentration of blocker set *N*' resulted in an increasingly higher efficiency of blocking. Percentages above dimer bands indicate dimer yields (mean ± SD, N=3).

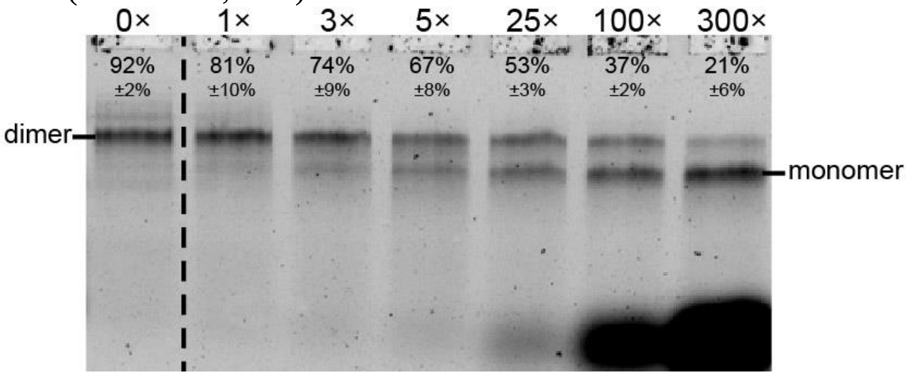

Supplementary Figure 27. Native agarose gel electrophoresis results of ON/OFF switch at pre-reaction state with fifteen pairs of sticky ends and fifteen blockers. Lane 0×: sample with binding switched ON; lanes n× (n=1, 3, 5, …, 300): samples with binding switched OFF. The incremental concentration of blocker set *N*' resulted in an increasingly higher efficiency of blocking. Percentages above dimer bands indicate dimer yields (mean ± SD, N=3).

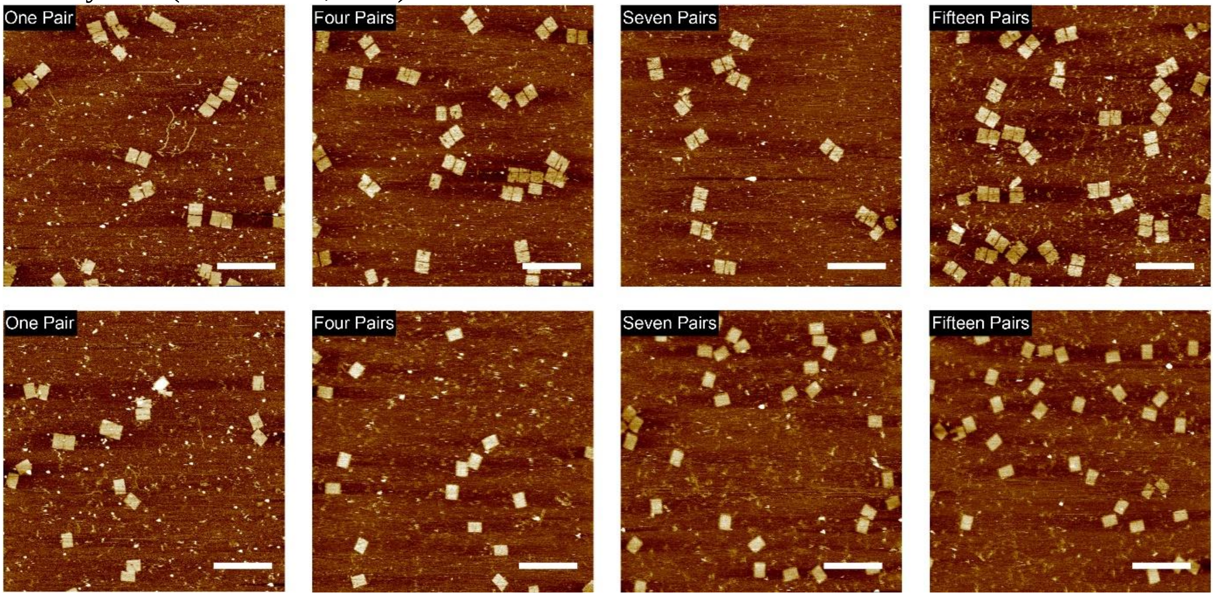

Supplementary Figure 28. AFM results of ON/OFF switched at pre-reaction state with different numbers of sticky end pairs (numbers of sticky end pairs displayed

on the AFM images). Top row: dimer product after gel-based purification. Bottom row: monomer product after gel-based purification. Scale bars: 400 nm.

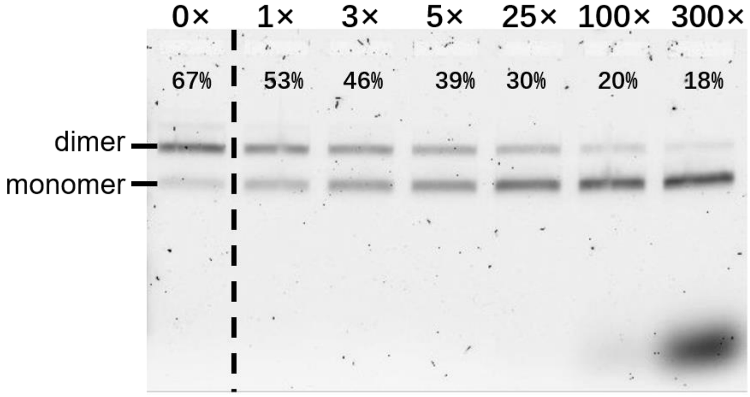

Supplementary Figure 29. Native agarose gel electrophoresis results of ON/OFF switch at pre-reaction state with seven pairs of exclusive 3' sticky ends and seven blockers with PAGE purified strands. Lane 0×: sample with binding switched ON; lanes n× (n=1, 3, 5, …, 300): samples with binding switched OFF. The trend and percentages of dimer are similar with that of above Supplementary Figure 26. Percentages above dimer bands indicate dimer yields.

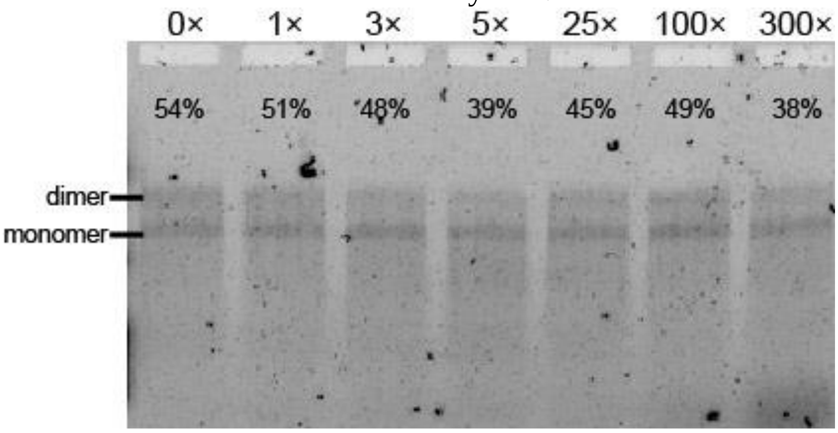

Supplementary Figure 30. Native agarose gel electrophoresis results of switch from ON to OFF with one pair of sticky end and one blocker. Lane 0×: sample with binding switched ON; lanes n× (n=1, 3, 5, …, 300): samples with binding switched from ON to OFF. Percentages above dimer bands indicate dimer yields.

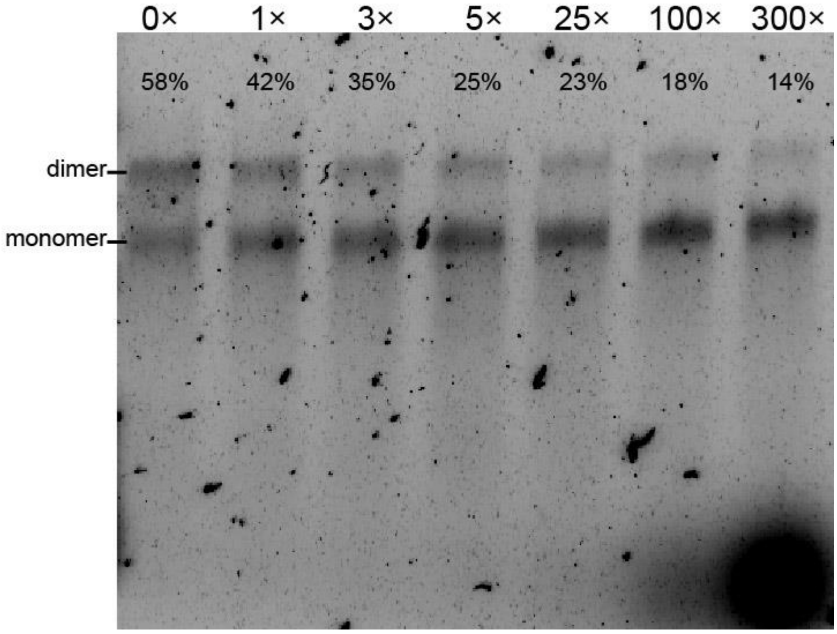

Supplementary Figure 31. Native agarose gel electrophoresis results of switch from ON to OFF with four pairs of sticky ends and four blockers. Lane 0×: sample with binding switched ON; lanes n× (n=1, 3, 5, …, 300): samples with binding switched from ON to OFF. Percentages above dimer bands indicate dimer yields.

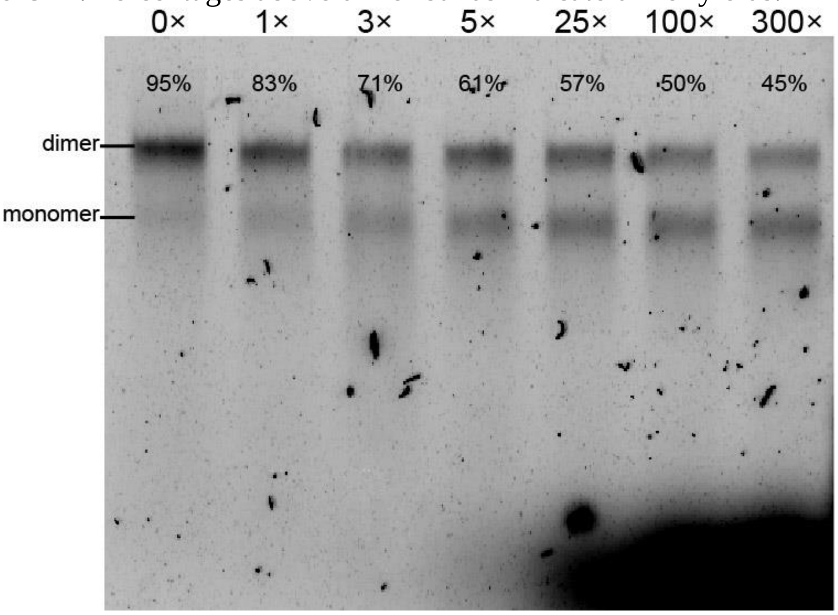

Supplementary Figure 32. Native agarose gel electrophoresis results of switch from ON to OFF with seven pairs of sticky ends and seven blockers. Lane 0×: sample with binding switched ON; lanes n× (n=1, 3, 5, …, 300): samples with binding switched from ON to OFF. Percentages above dimer bands indicate dimer yields.

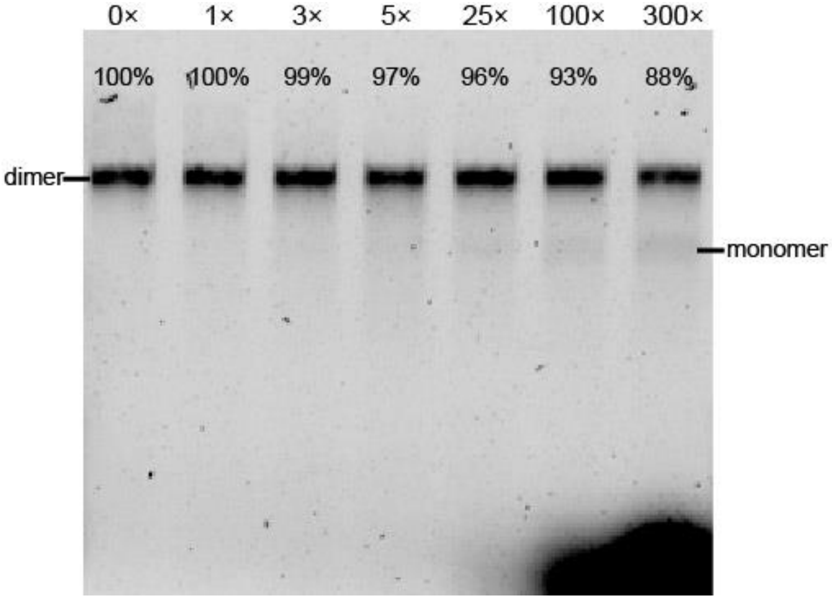

Supplementary Figure 33. Native agarose gel electrophoresis results of switch from ON to OFF with fifteen pairs of sticky ends and fifteen blockers. Lane 0×: sample with binding switched ON; lanes n× (n=1, 3, 5, …, 300): samples with binding switched from ON to OFF. Percentages above dimer bands indicate dimer yields.

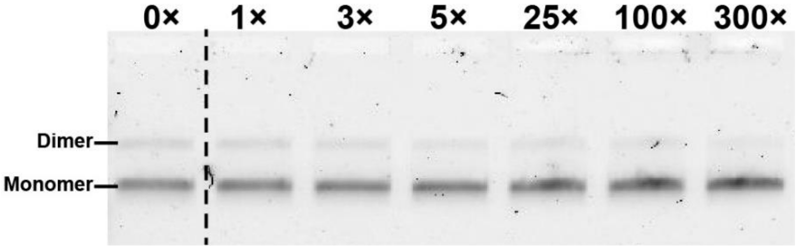

Supplementary Figure 34. Native agarose gel electrophoresis results of ON/OFF switch at pre-reaction state with seven pairs of sticky ends and seven blockers. The length of binding sticky end is 8-nt. Lane 0×: sample with binding switched ON; lanes n× (n=1, 3, 5, …, 300): samples with binding switched from ON to OFF. The switch into OFF is inapparent.

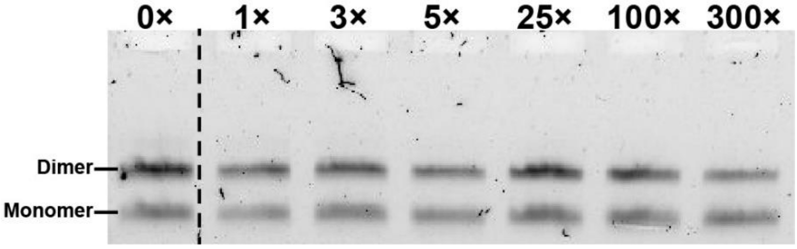

Supplementary Figure 35. Native agarose gel electrophoresis results of switch from ON to OFF with seven pairs of sticky ends and seven blockers. The length of binding sticky end is 8-nt. Lane 0×: sample with binding switched ON; lanes n× (n=1, 3, 5, …, 300): samples with binding switched from ON to OFF. The switch from ON to OFF is inapparent.

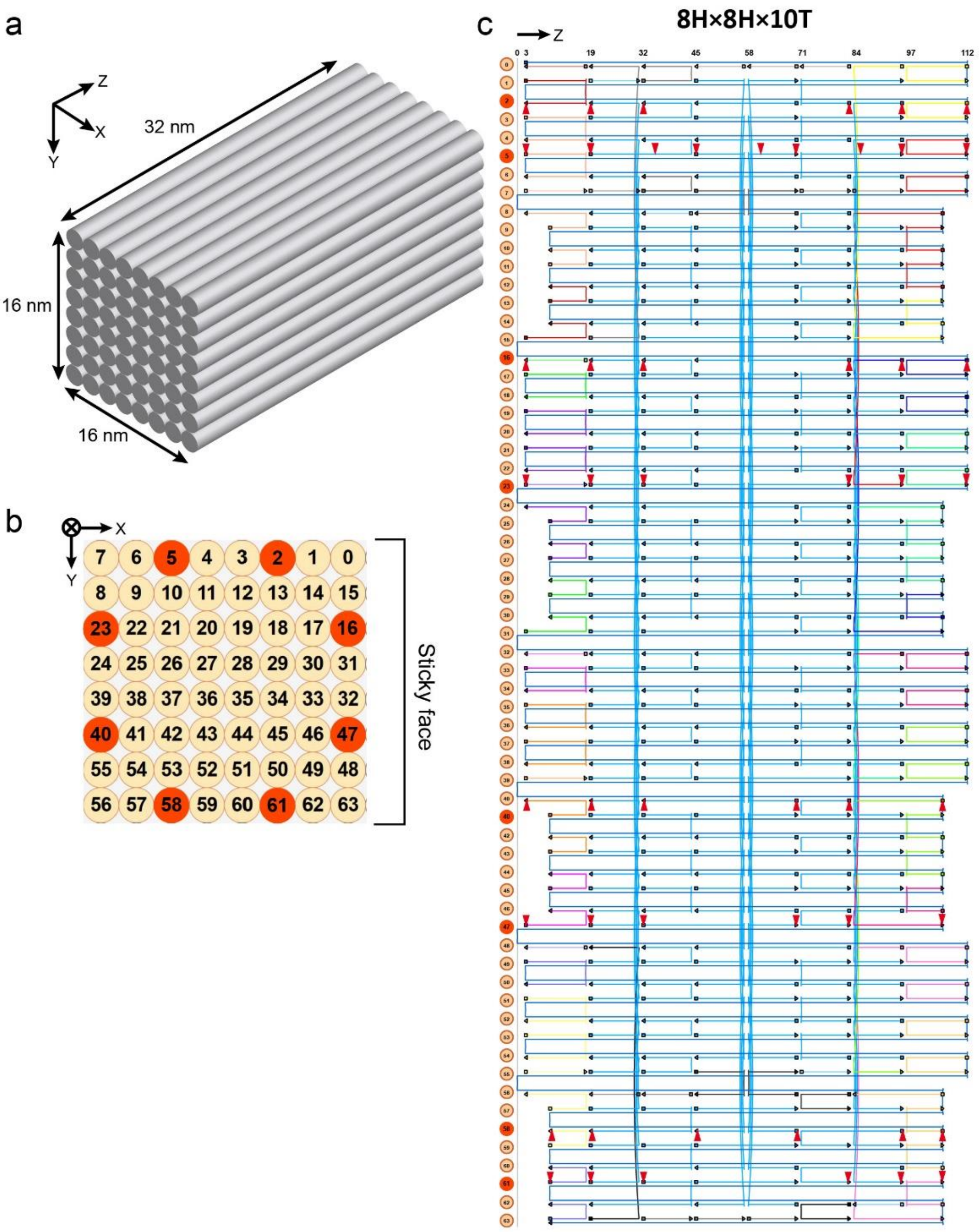

Supplementary Figure 36. Design and strand diagram of the origami cuboid (8H×8H×10T). (a) A cylinder model. (b) Cross-section view, looking down the Z+ direction of the cuboid. The orangered circles highlight the sites where connection staples locate. (c) Detailed strand diagram. Red triangles point to the connection staple position at which the combined segment of the linker and the sticky end are extended. Details available when zoomed in.

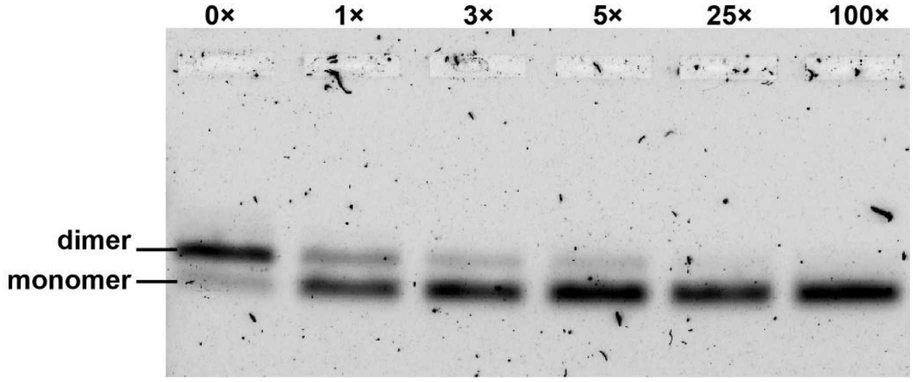

Supplementary Figure 37. Native agarose gel electrophoresis results of 2-bit input/1-bit output system. Lane 0×: sample with binding switched ON; lanes n× (n=1, 3, 5, 25, 100): samples with binding switched OFF. The incremental concentration of blocker set *N*’ resulted in an increasingly higher efficiency of blocking. At 100× concentration, no dimer product was available.

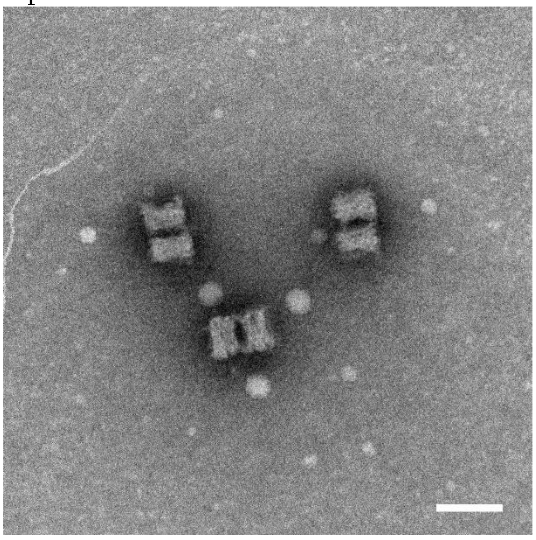

Supplementary Figure 38. TEM image of origami cuboid dimer as an ON state. Scale bar: 50 nm.

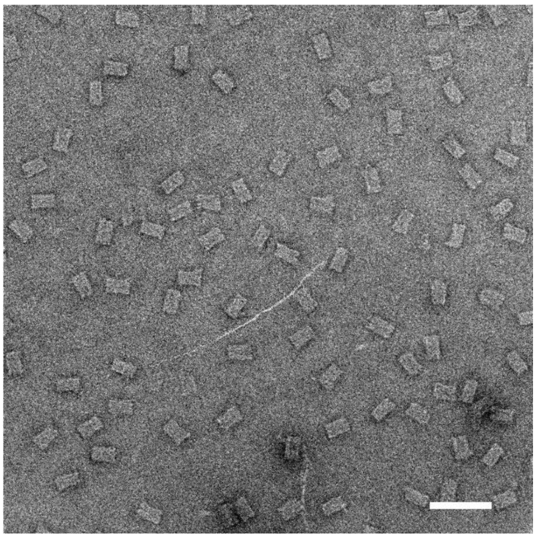

Supplementary Figure 39. TEM image of origami cuboid monomer as an OFF state. Scale bar: 100 nm.

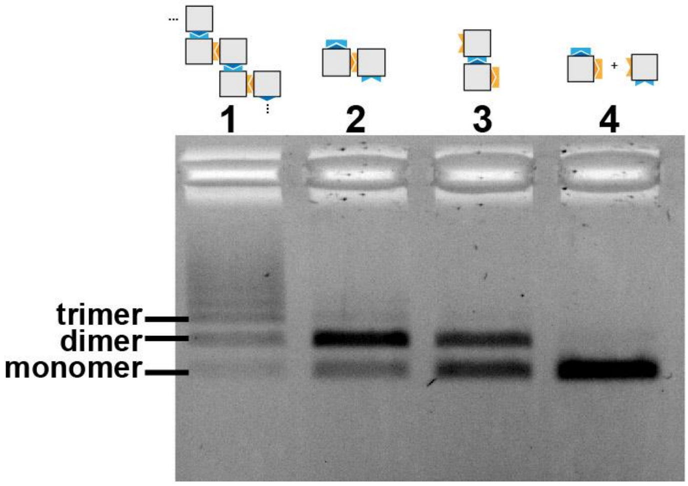

Supplementary Figure 40. Native agarose gel electrophoresis results of 4-bit input/2-bit input system. Lane 1: the absence of both *N*' and *M*' resulted in multimers; lane 2: the presence of either *N*' resulted in dimer; lane 3: the presence of either *M*' resulted in dimer; lane 4: the presence of both *N*' and *M*' resulted in monomer.

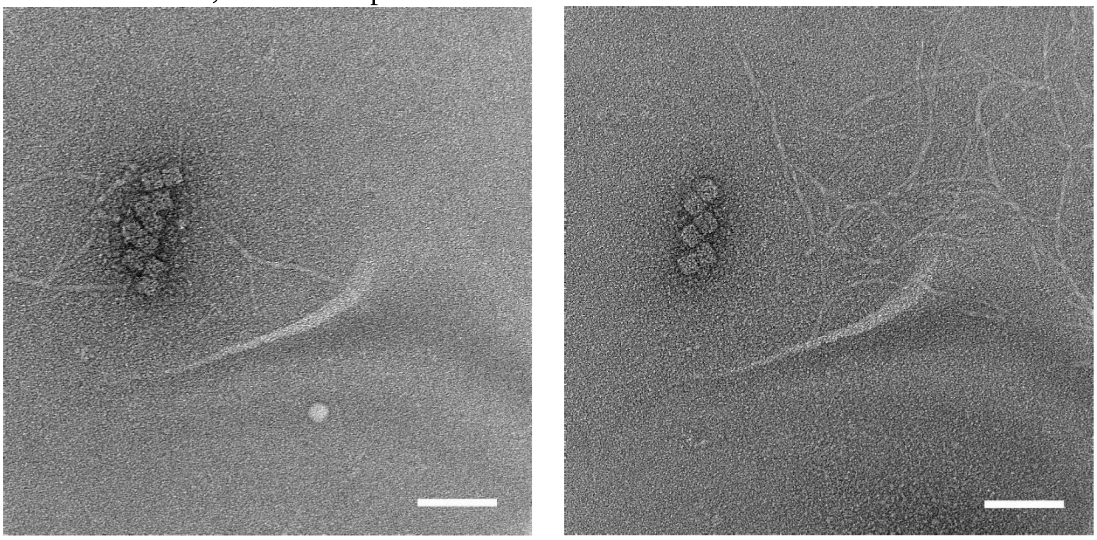

Supplementary Figure 41. TEM images of origami cuboid multimers. Scale bars: 100 nm.

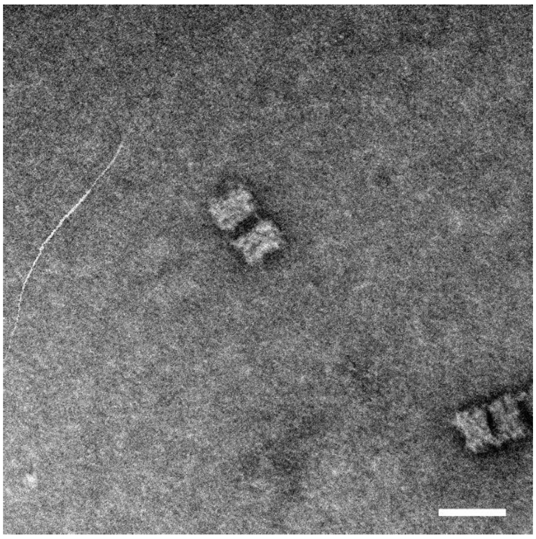

Supplementary Figure 42. TEM image of origami cuboid dimer with blocker *N*'. Scale bar: 50 nm.

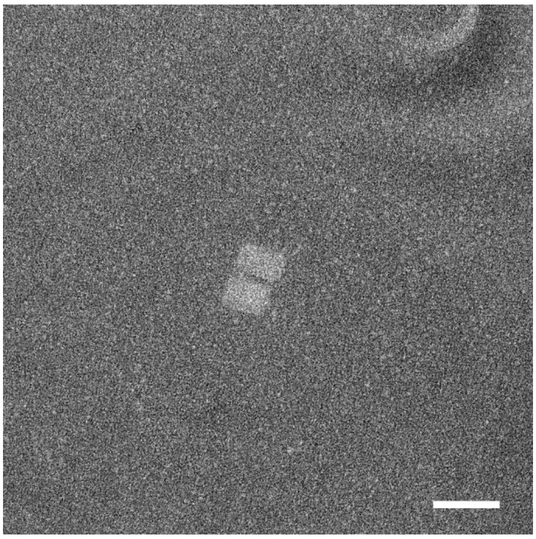

Supplementary Figure 43. TEM image of origami cuboid dimer with blocker *M*'. Scale bar: 50 nm.

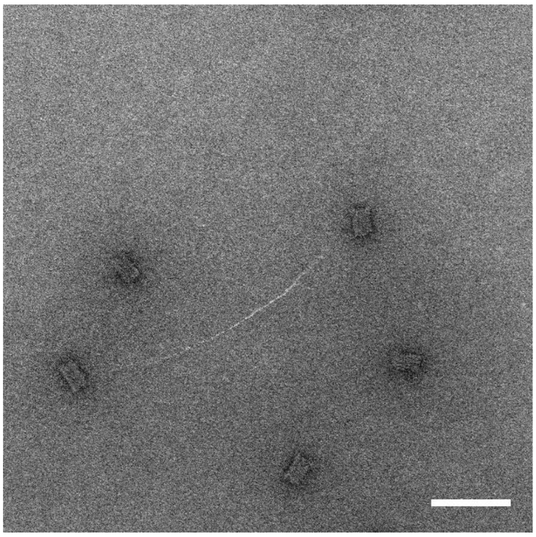

Supplementary Figure 44. TEM image of origami cuboid monomer with blockers *N*' and *M*'. Scale bar: 100 nm.

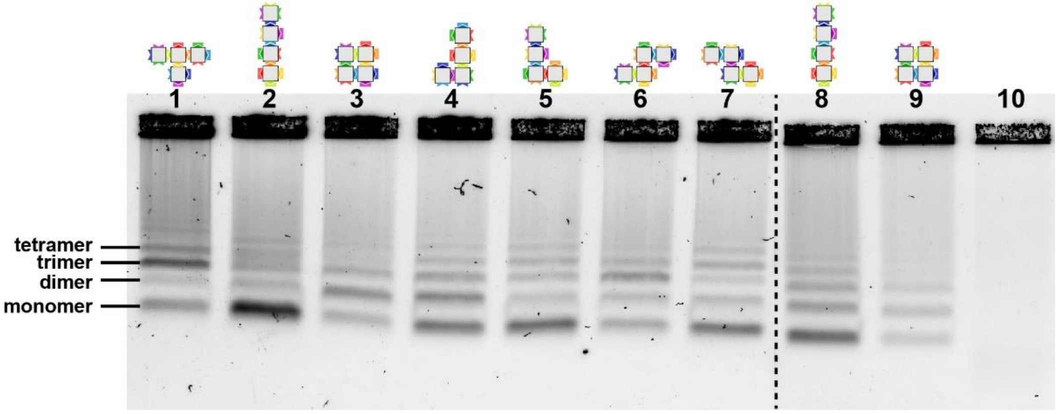

Supplementary Figure 45. Native agarose gel electrophoresis results of seven tetrominoes assembled from individual PEG-purified units. Lane 1: T-tetromino; lanes 2 and 8: I-tetromino; lanes 3 and 9: O-tetromino; lane 4: J-tetromino; lane 5: L-tetromino; lane 6: S-tetromino; lane 7: Z-tetromino; lane 10: control without blockers.

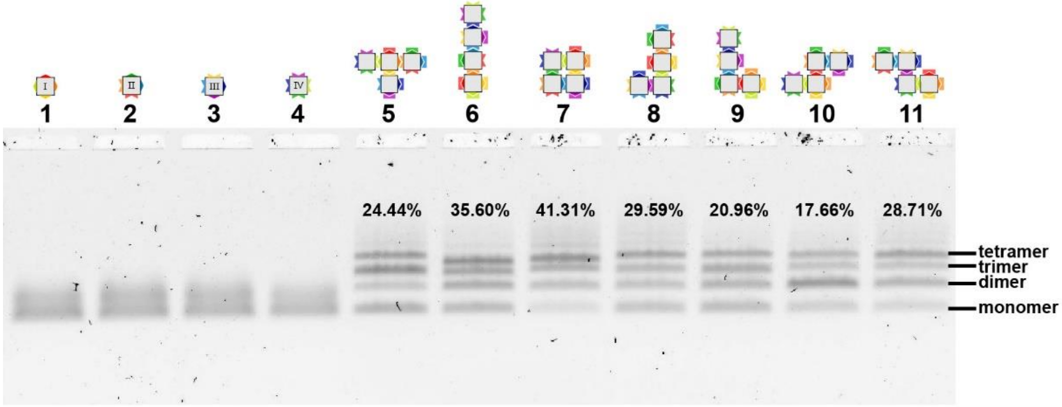

Supplementary Figure 46. Native agarose gel electrophoresis results of seven tetrominoes assembled from individual gel-purified units. Lane 1: cuboid I; lane 2: cuboid II; lane 3: cuboid III; lane 4: cuboid IV; lane 5: T-tetromino; lane 6: I-tetromino; lane 7: O-tetromino; lane 8: J-tetromino; lane 9: L-tetromino; lane 10: S-tetromino; lane 11: Z-tetromino. Percentages above tetramer bands indicate tetramer yields.

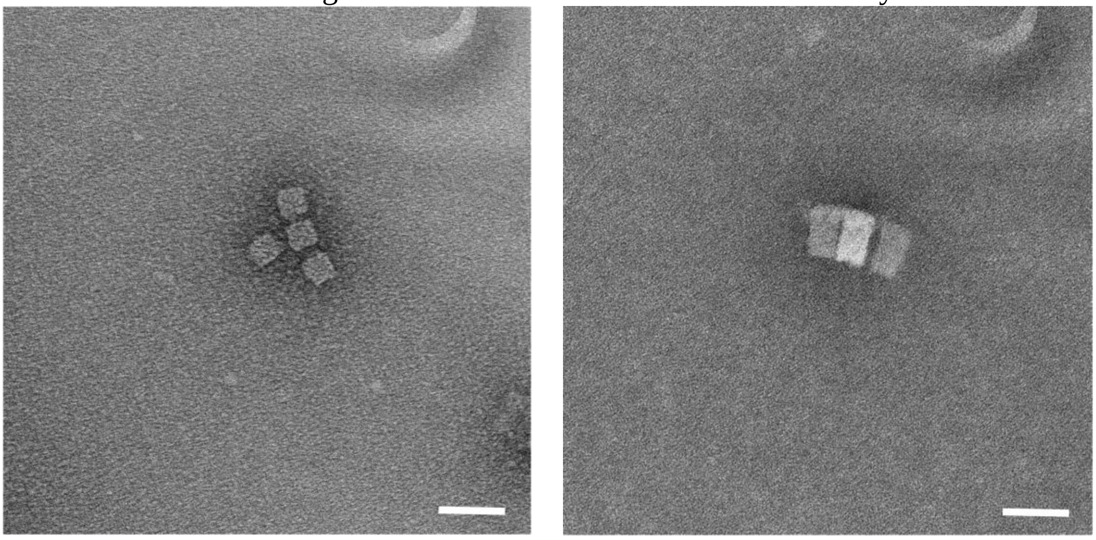

Supplementary Figure 47. TEM images of T-tetromino of different landing orientations. Scale bars: 50 nm.

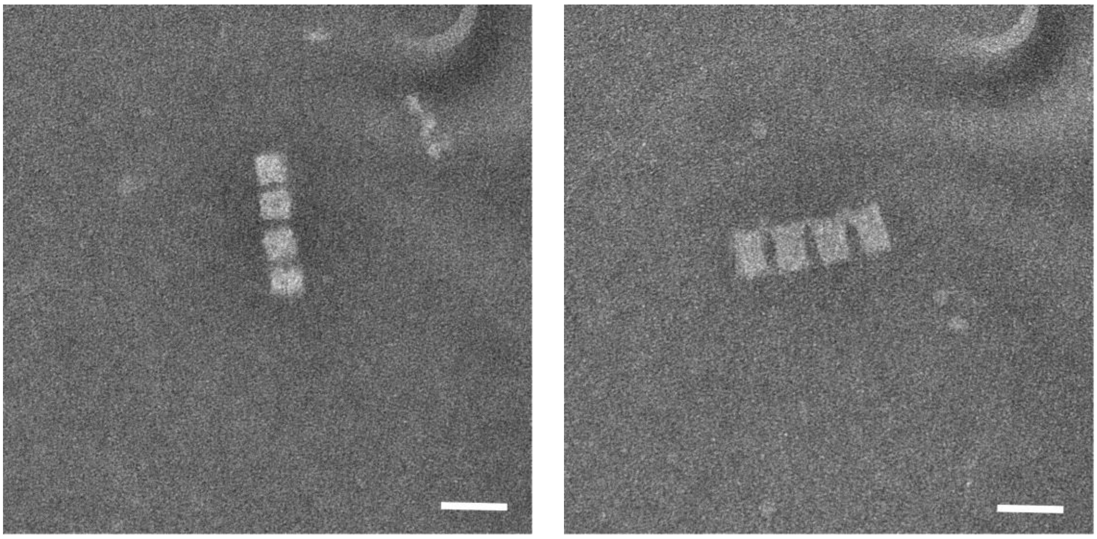

Supplementary Figure 48. TEM images of I-tetromino of different landing orientations. Scale bars: 50 nm.

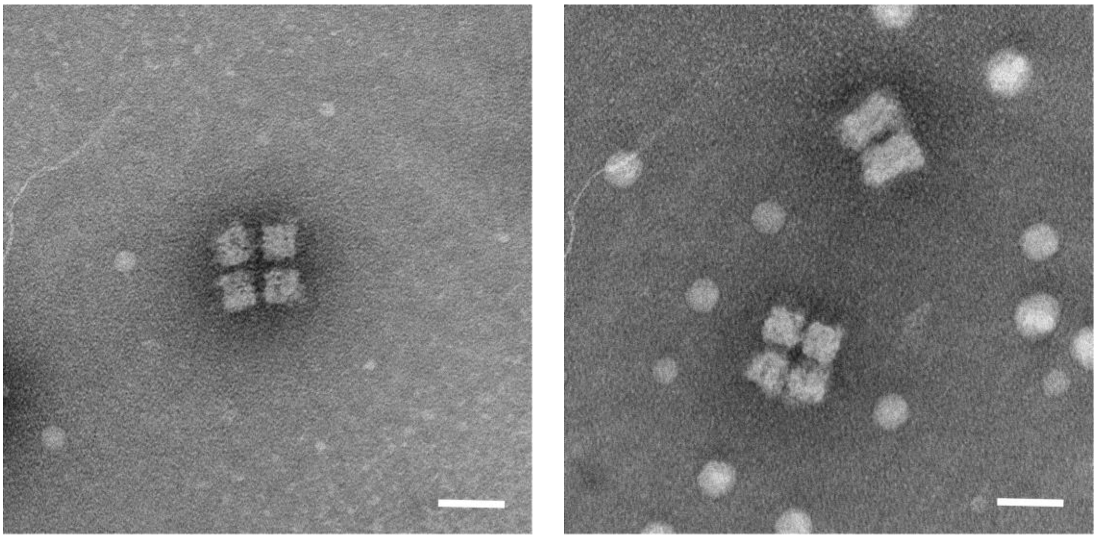

Supplementary Figure 49. TEM images of O-tetromino of different landing orientations. Scale bars: 50 nm.

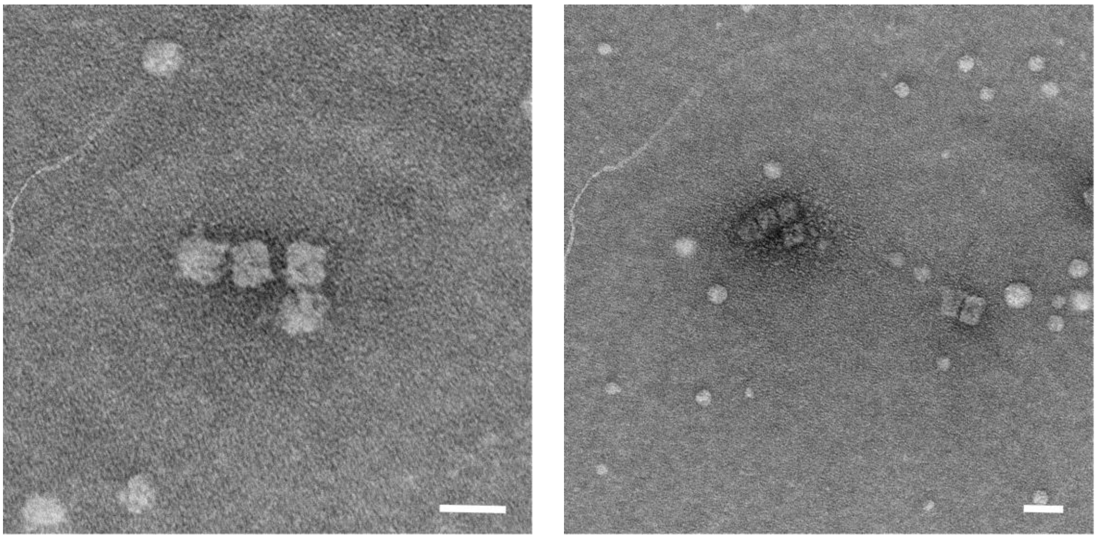

Supplementary Figure 50. TEM images of J-tetromino of different landing orientations. Scale bars: 50 nm.

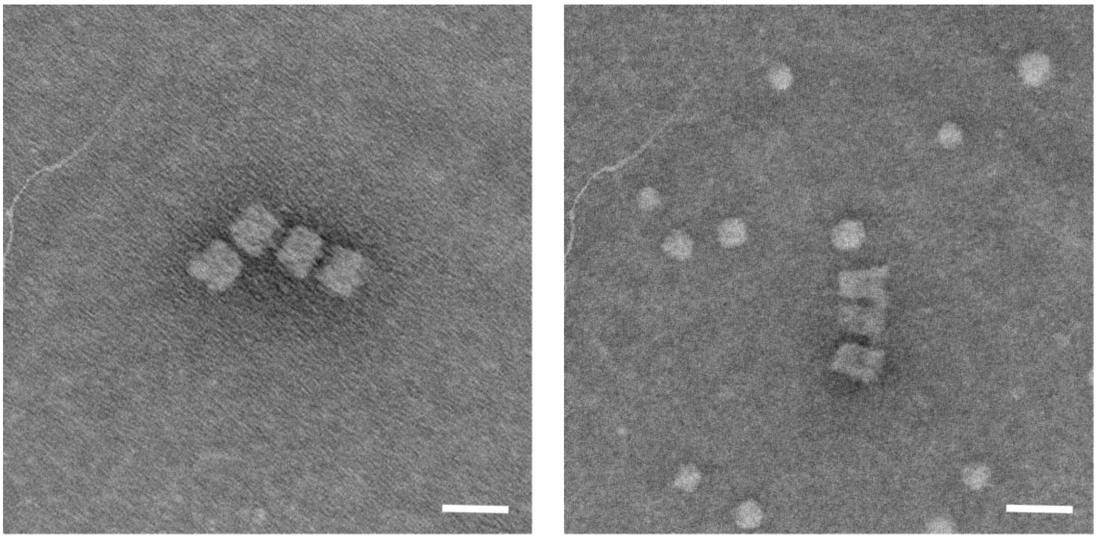

Supplementary Figure 51. TEM images of L-tetromino of different landing orientations. Scale bars: 50 nm.

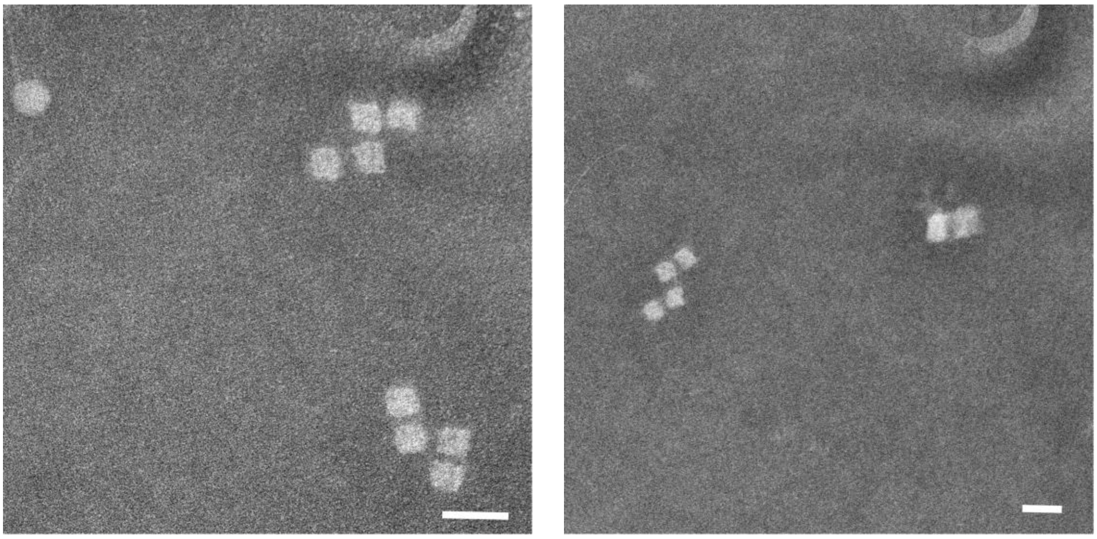

Supplementary Figure 52. TEM images of S-tetromino of different landing orientations. Scale bars: 50 nm.

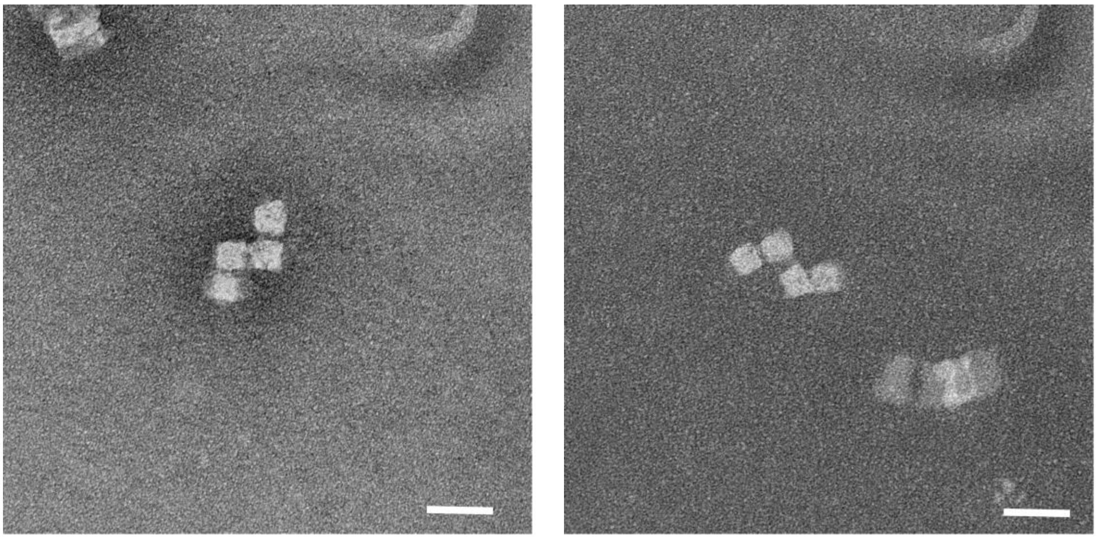

Supplementary Figure 53. TEM images of Z-tetromino of different landing orientations. Scale bars: 50 nm.

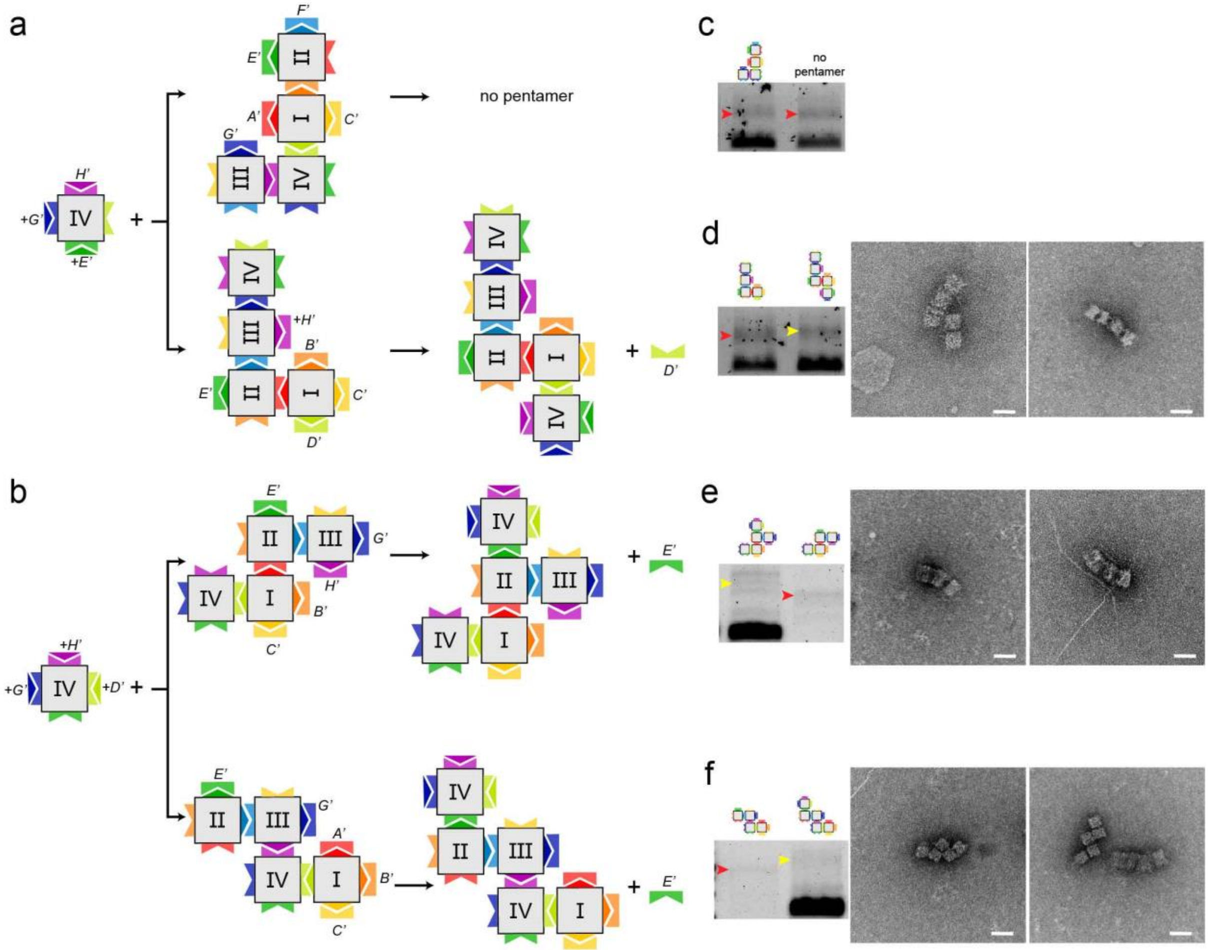

Supplementary Figure 54. Mirrored shape identification. (a) Schematic diagram of J-/L-tetrominoes identification. Preformed origami cuboid IV with blockers were excessively added to J-tetromino and L-tetromino, respectively, and pentamer only formed from L-tetromino but not from J-tetromino. (b) Schematic diagram of S-/Z-tetrominoes identification. Preformed origami cuboid IV with blockers were excessively added to S-tetromino and Z-tetromino, respectively, and pentamers of a specific shape formed from either tetromino. (c-f) Native agarose gel electrophoresis and TEM results. Left: native agarose gel electrophoresis results; right: TEM images of pentamers. Red arrows point to tetramer bands. Yellow arrows point to pentamer bands. Scale bars: 50 nm.

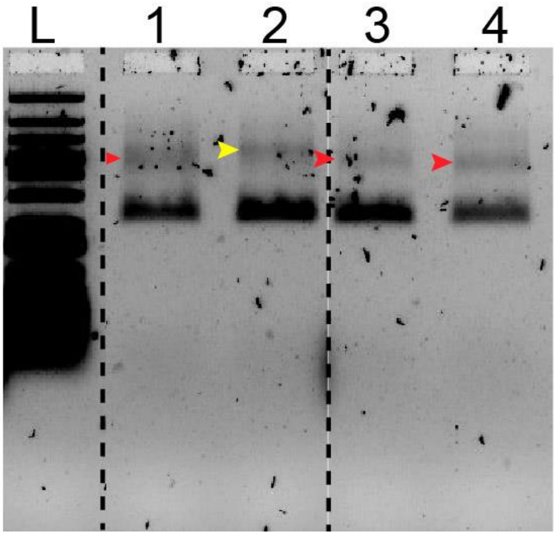

Supplementary Figure 55. Native agarose gel electrophoresis result of J-/L-tetrominoes identification. Lane L: 1 kb plus. Lane 1: Preformed origami L-tetromino. Lane 2: Preformed origami cuboid IV with blockers were excessively added to L-tetromino. Lane 3: Preformed origami J-tetromino. Lane 4: Preformed origami cuboid IV with blockers were excessively added to J-tetromino. Red triangles point to tetramer bands. Yellow triangle points to pentamer bands.

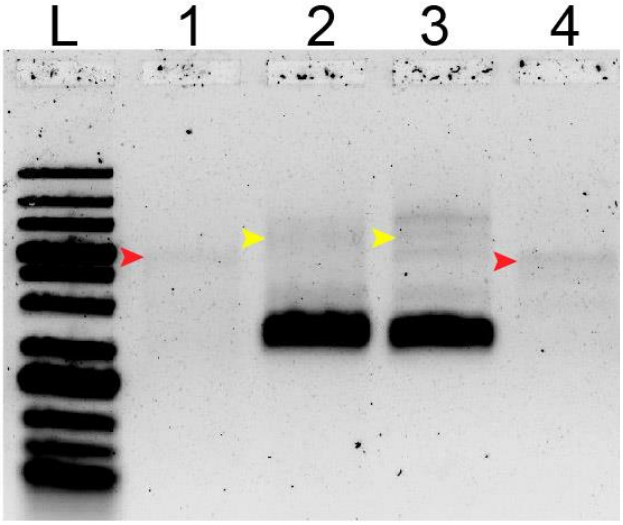

Supplementary Figure 56. Native agarose gel electrophoresis result of S-/Z-tetrominoes identification. Lane L: 1 kb plus. Lane 1: Preformed origami Z-tetromino. Lane 2: Preformed origami cuboid IV with blockers were excessively added to Z-tetromino. Lane 3: Preformed origami cuboid IV with blockers were excessively added to S-tetromino. Lane 3: Preformed origami S-tetromino. Red triangles point to tetramer bands. Yellow triangle points to pentamer bands.

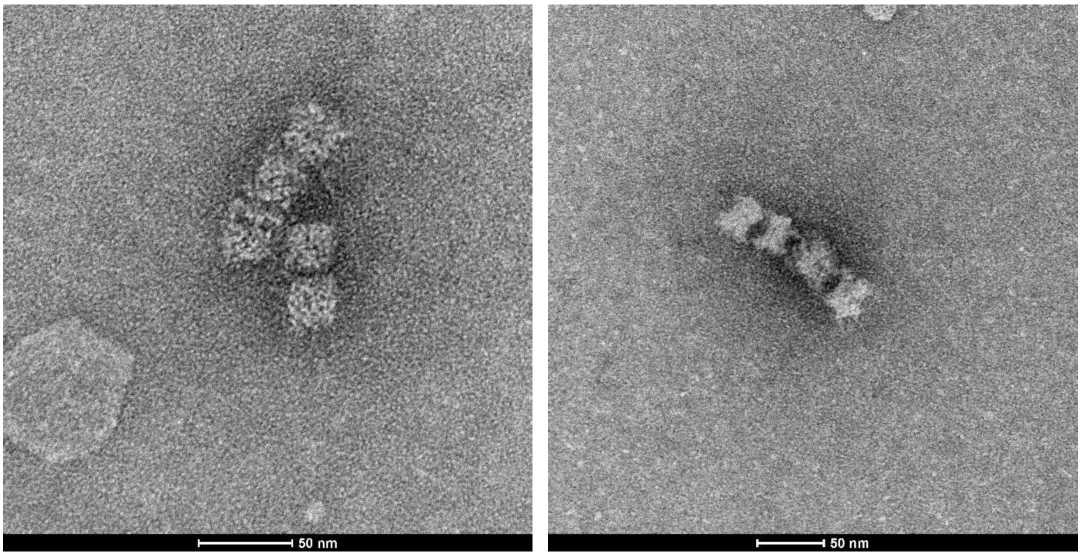

Supplementary Figure 57. TEM images of pentamer formed from L-tetromino of different landing orientations. Scale bars: 50 nm.

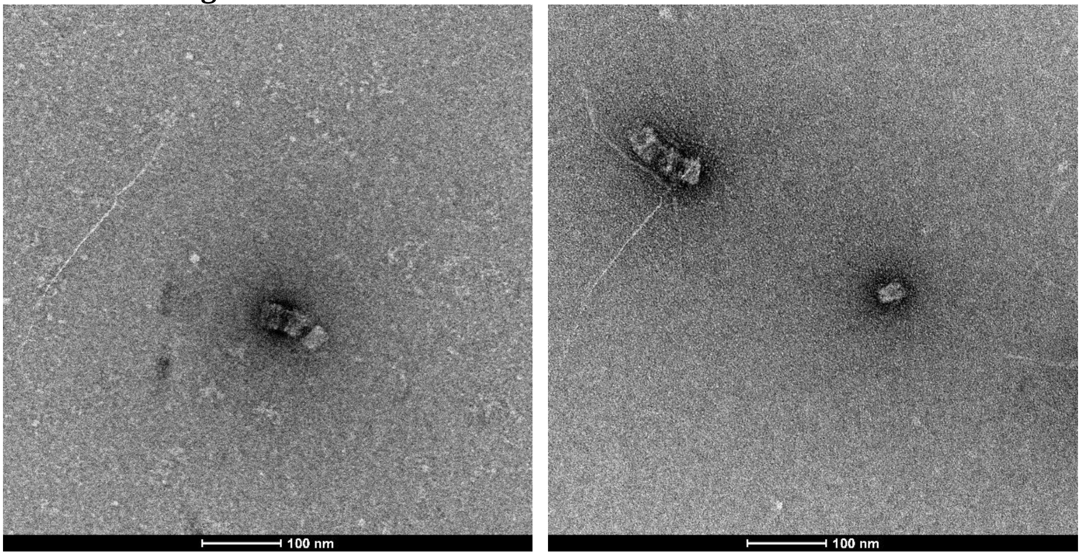

Supplementary Figure 58. TEM images of pentamer formed from S-tetromino of different landing orientations. Scale bars: 50 nm.

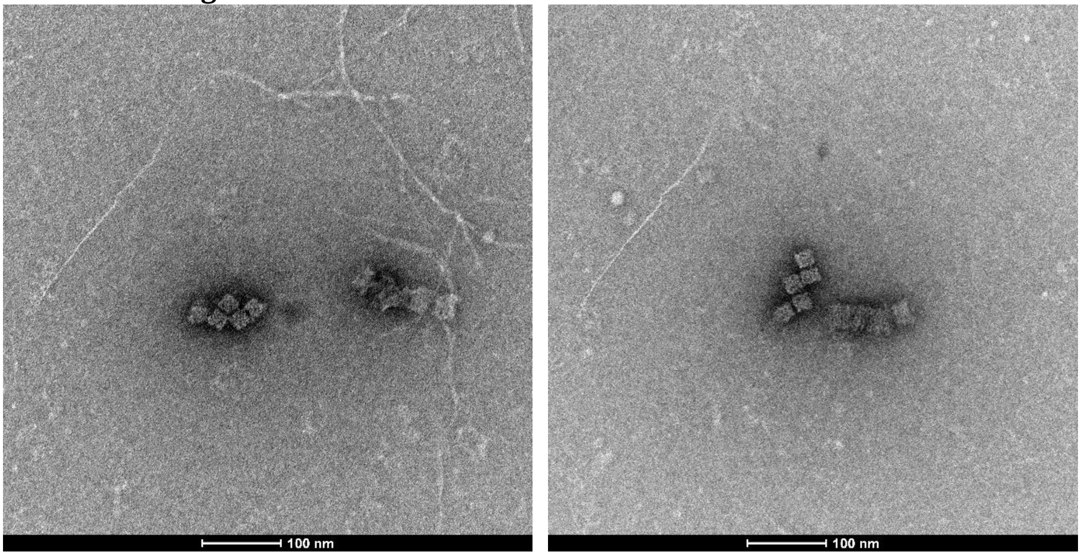

Supplementary Figure 59. TEM images of pentamer formed from Z-tetromino of

different landing orientations. Scale bars: 50 nm.

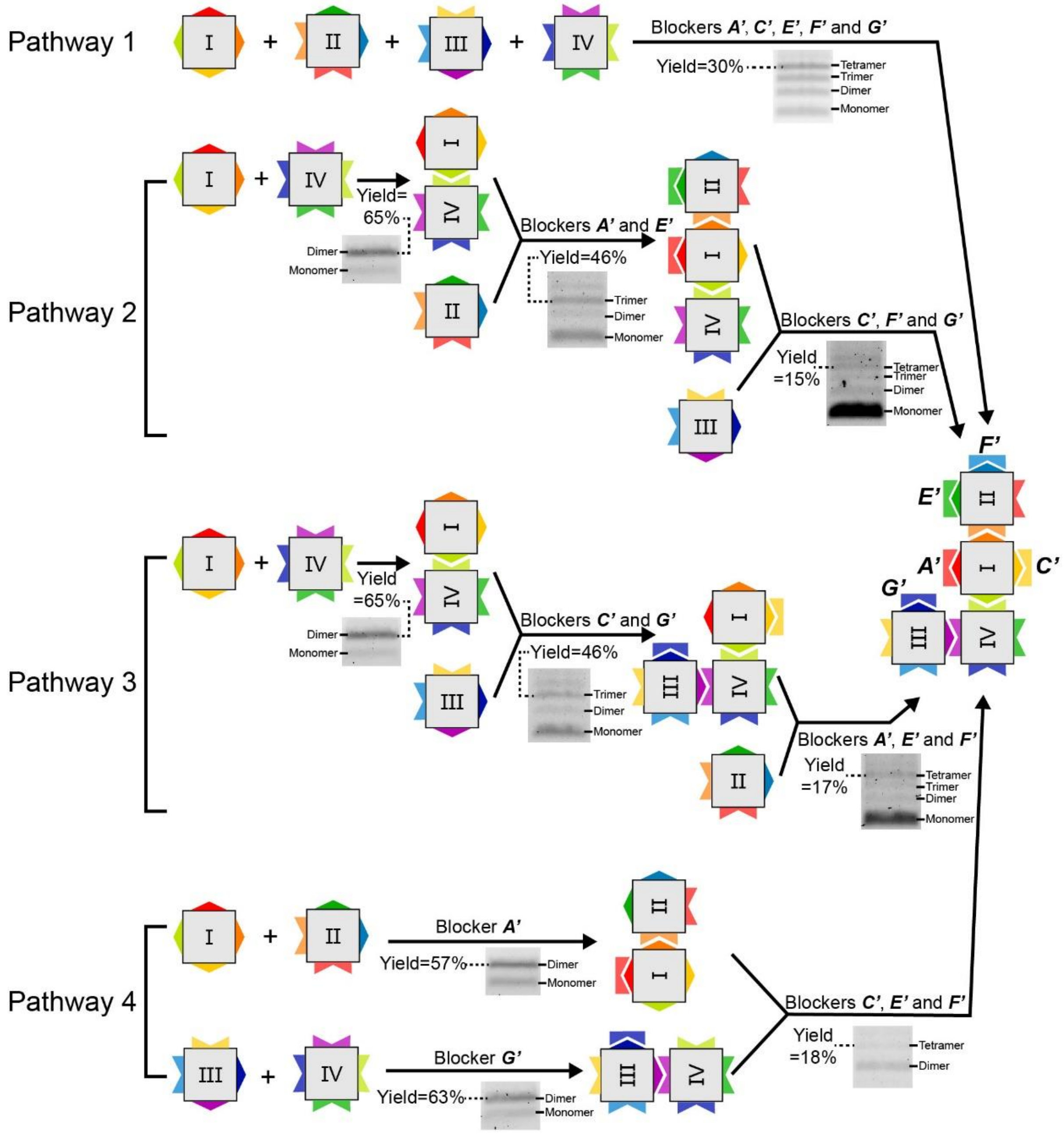

Supplementary Figure 60. Schematic diagrams and results of alternative pathways to assemble the J-tetromino. Pathway 1: one-pot reaction of I+II+III+IV. The yield of pathway 1 is 30% (full gel image in Supplementary Figure 46.). Pathway 2: hierarchical reaction of ((I+IV)+II)+III. The final yield of pathway 2 = 65% × 46% × 15% = 5%. Pathway 3: hierarchical reaction of ((I+IV)+III)+II. The final yield of pathway 3 = 65% × 46% × 17% = 5%. Pathway 4: hierarchical reaction of (I+II)+(III+IV). The final yield of pathway 4 = 57% × 18% = 10%. The yields of J-tetromino of pathways 2-4 are significantly lower than that of pathway 1.

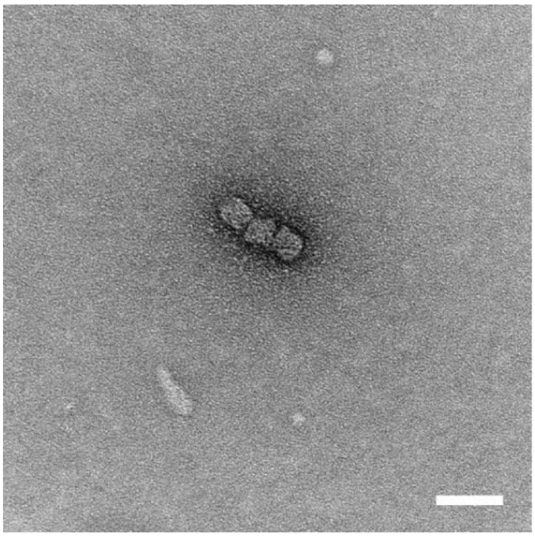

Supplementary Figure 61. TEM image of (I+IV)+II trimer product of pathway 2. Scale bar: 50 nm.

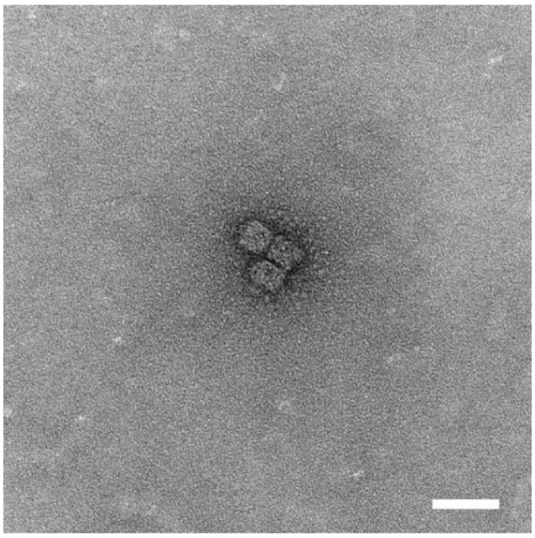

Supplementary Figure 62. TEM image of (I+IV)+III trimer product of pathway 3. Scale bar: 50 nm.

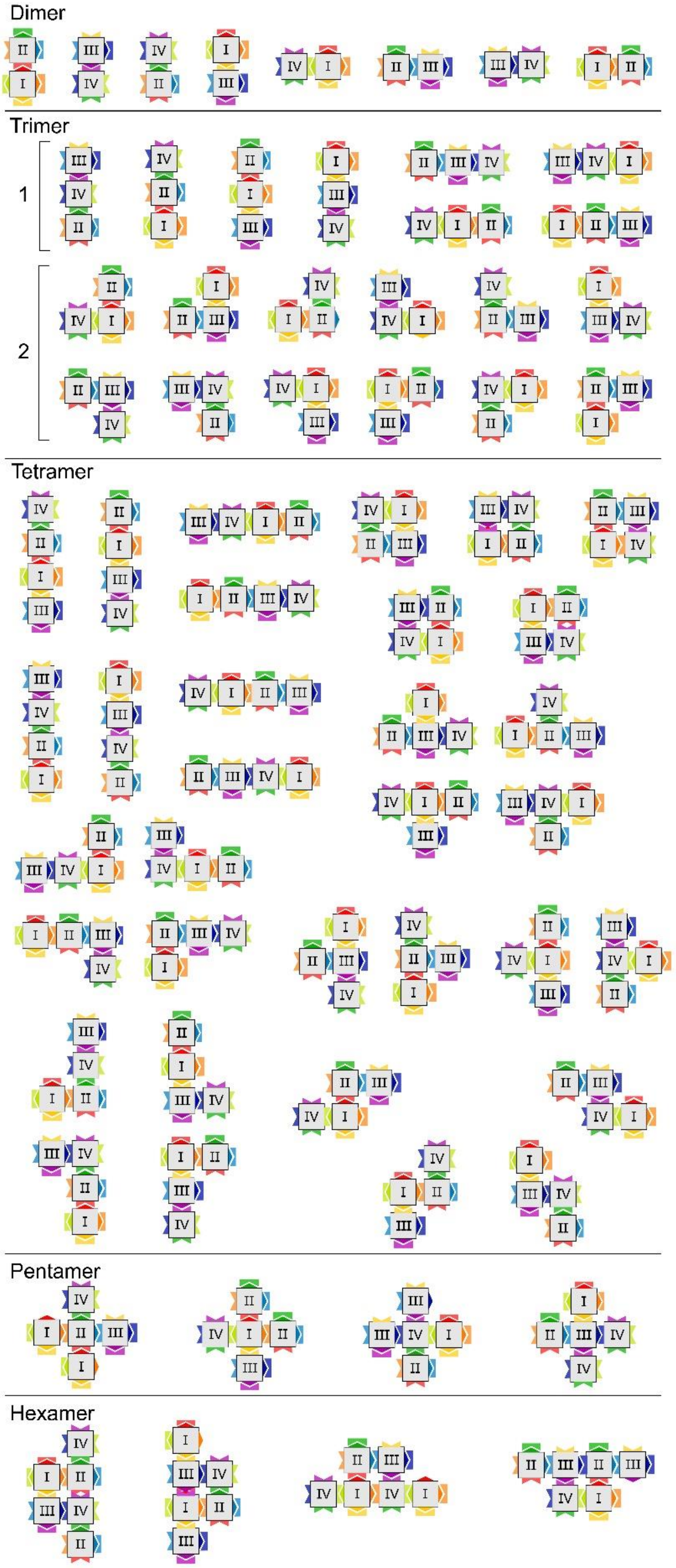

Supplementary Figure 63. Schematic diagrams of 69 infinite shapes, including dimer, trimer, tetramer, pentamer and hexamer.

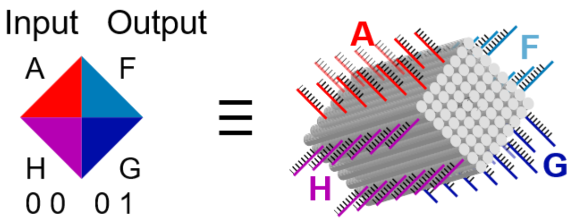

Supplementary Figure 64. Schematic of 3D cuboid DNA origami tile. Twelve sticky ends are extended from each sticky face. The left two sticky faces work as input, and the right two sticky faces work as output that build a 2-bit circuit element.

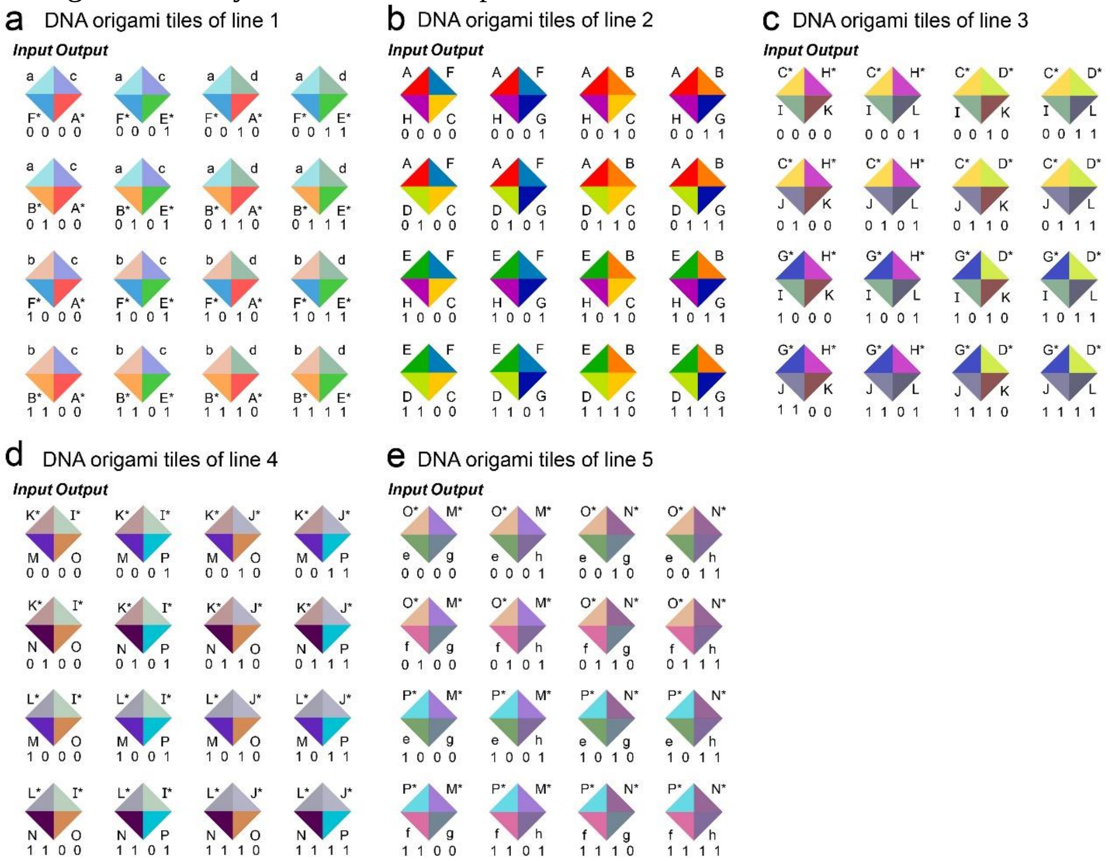

Supplementary Figure 65. Schematics of 80 kinds of different 3D cuboid DNA origami tiles. The lettered sticky face is complementary with the star lettered sticky face (e.g., A/A*). The DNA origami tiles of line 1 (a) bind with that of line 2 (b), and the rest can bind to each other line by line. Then the sticky faces with small letter can bind with boundary origami tiles (not supported here). The different combination of tiles can operate 6-bit input different algorithms.

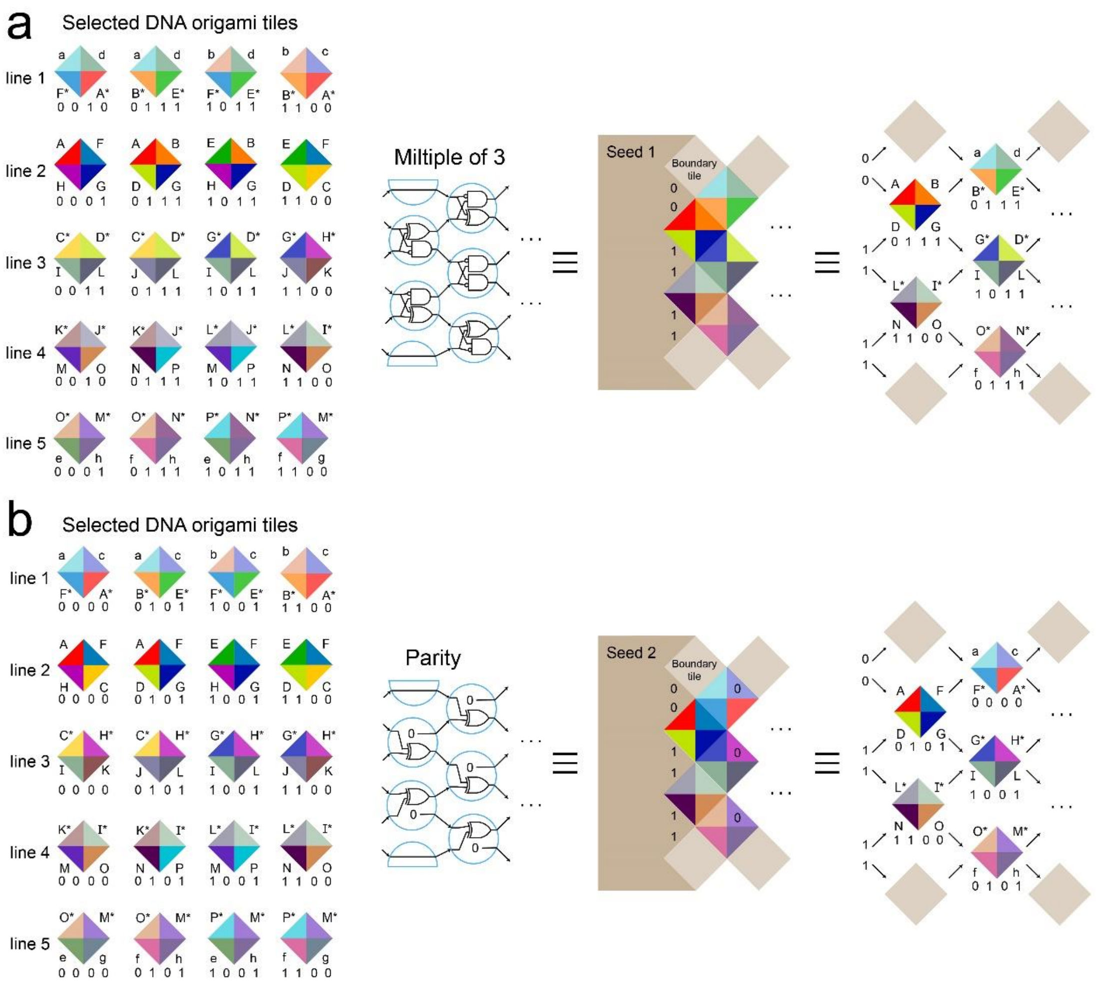

Supplementary Figure 66. Schematics of two 6-bit input algorithms. Multiple of 3 (a) and Parity (b). The specific DNA origami tiles are selected from Supplementary Figure 65 that shown at the left column. The origami tiles from different lines locate at their position when operate the algorithm. The light brown squares present the boundary DNA origami tiles that are used to constrain the shapes. The circuits abstract the operation processes.

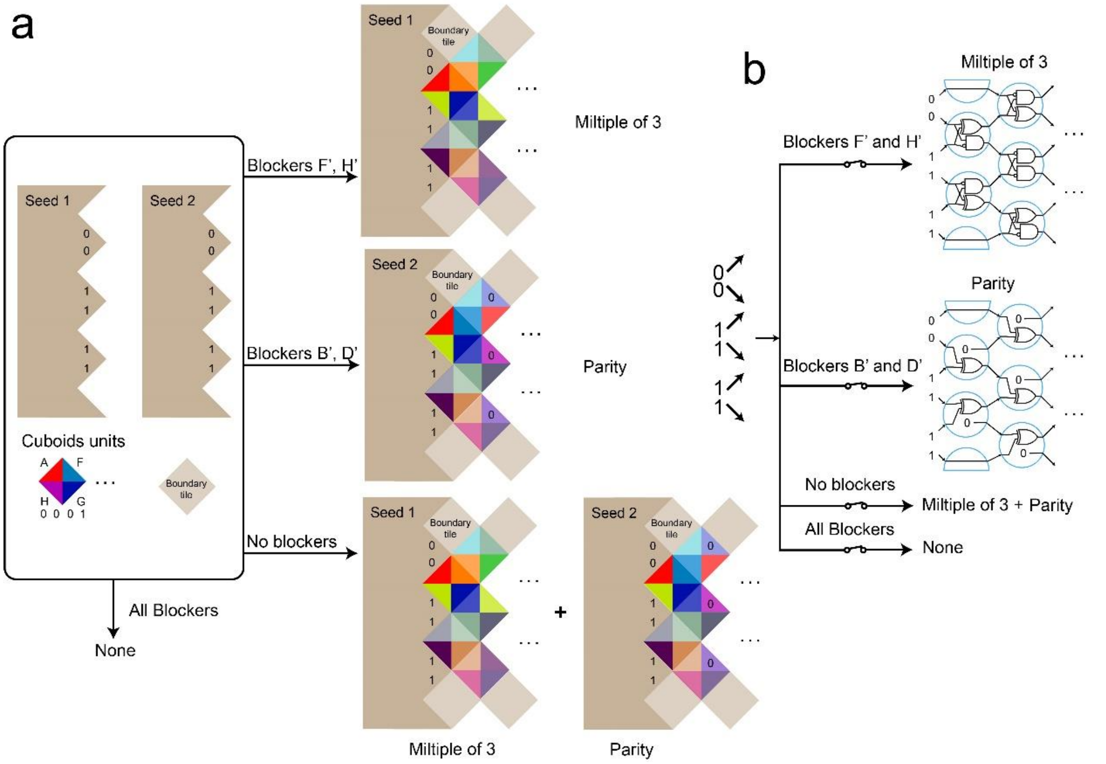

Supplementary Figure 67. Schematics of an example that a specific Boolean function could be implemented by a specific combination of blockers. (a) The mixture contains two kinds of origami seeds and corresponding origami tiles. The Multiple of 3 will be executed with the excess blockers F' and H'. The Parity will be executed with the excess blockers B' and D'. These two algorithms will both executed without the control of any blockers. And the algorithms will be blocked by added both blockers. (b) The circuitry is used to abstract the control process.

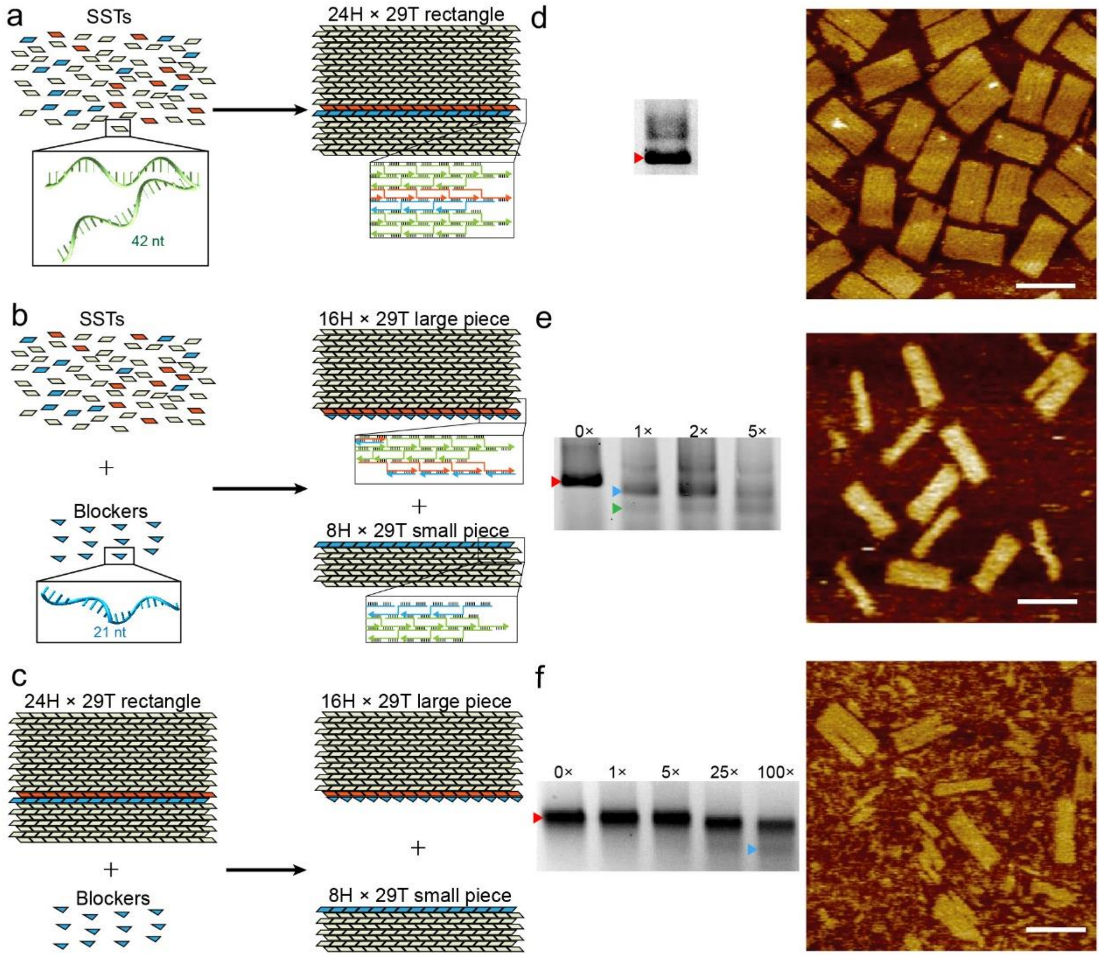

Supplementary Figure 68. Split-up of SST rectangle structure (24H×29T). (a) Schematic diagram of self-assembly of 24H×29T rectangle. (b) Schematic diagram of ON/OFF switch from pre-reaction state. Component SSTs (1×, 105 nM) and blockers at gradient concentrations (0×, 1×, 2× and 5×) were mixed in 0.5× TBE buffer supplemented with 15 mM $MgCl_2$. Samples were subjected to an isothermal incubation (65℃ for 10 minutes and 45.7℃ for 14 hours) before characterization. (c) Schematic diagram of switch from ON to OFF. Preformed rectangle structures after purification were mixed with blockers at gradient concentrations (0×, 1×, 5×, 25× and 100×). Samples were subjected to an isothermal incubation (45.7℃ for 40 hours) before characterization. (d, e, f) Corresponding native agarose gel electrophoresis (left) and AFM (right) results of (a, b, c). Red triangles point to 24H×29T rectangle bands. Blue triangles point to large piece bands. Green triangles point to small piece bands. Scale bars: 400 nm.

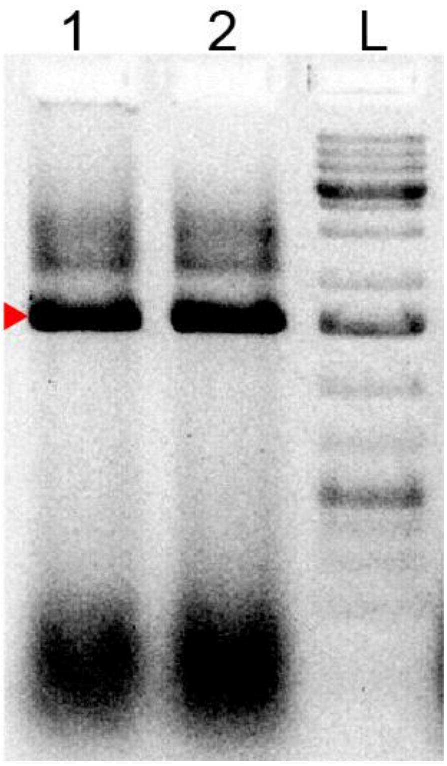

Supplementary Figure 69. Native agarose gel electrophoresis result of split-up of SST rectangle structure (24H×29T) in lanes 1 and 2. Lane L: 1 kb plus. DNA ladder. Red triangles point to 24H×29T rectangle bands.

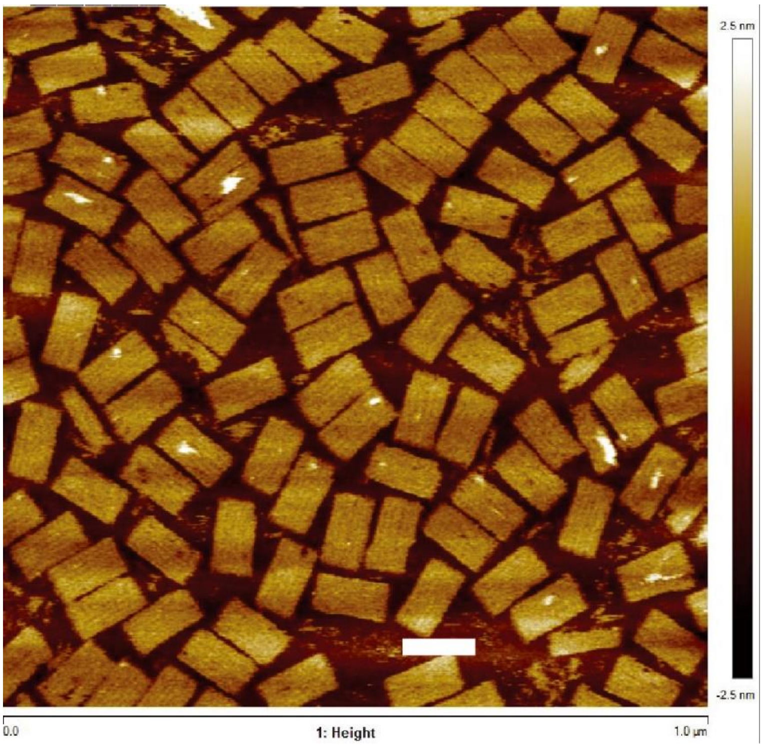

Supplementary Figure 70. AFM result of split-up of SST rectangle structure (24H×29T). Scale bars: 400 nm.

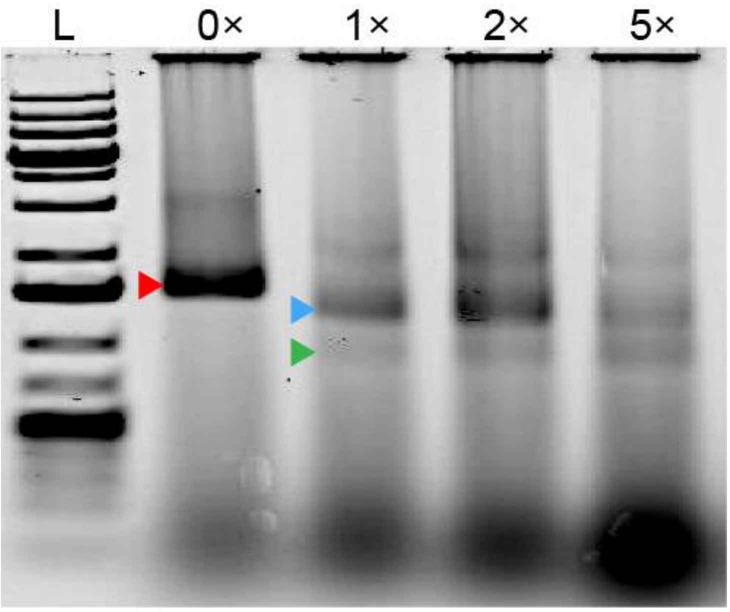

Supplementary Figure 71. Native agarose gel electrophoresis result of ON/OFF switch from pre-reaction state. Red triangles point to 24H×29T rectangle bands. Lane L: 1 kb plus. Blue triangles point to large piece bands. Green triangles point to small piece bands.

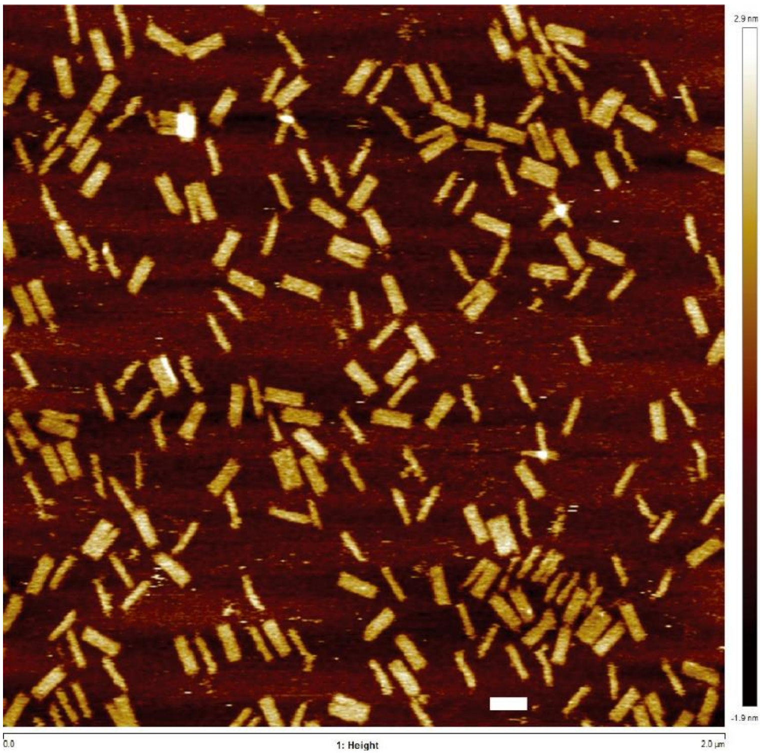

Supplementary Figure 72. AFM result of ON/OFF switch from pre-reaction state. Scale bars: 400 nm.

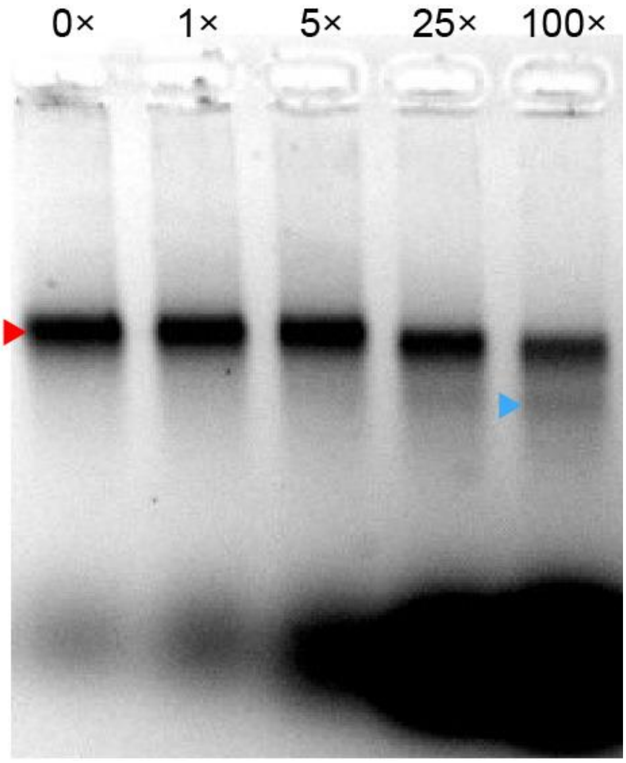

Supplementary Figure 73. Native agarose gel electrophoresis result of switch from ON to OFF. Lane L: 1 kb plus. Red triangles point to 24H×29T rectangle bands. Blue triangles point to large piece bands.

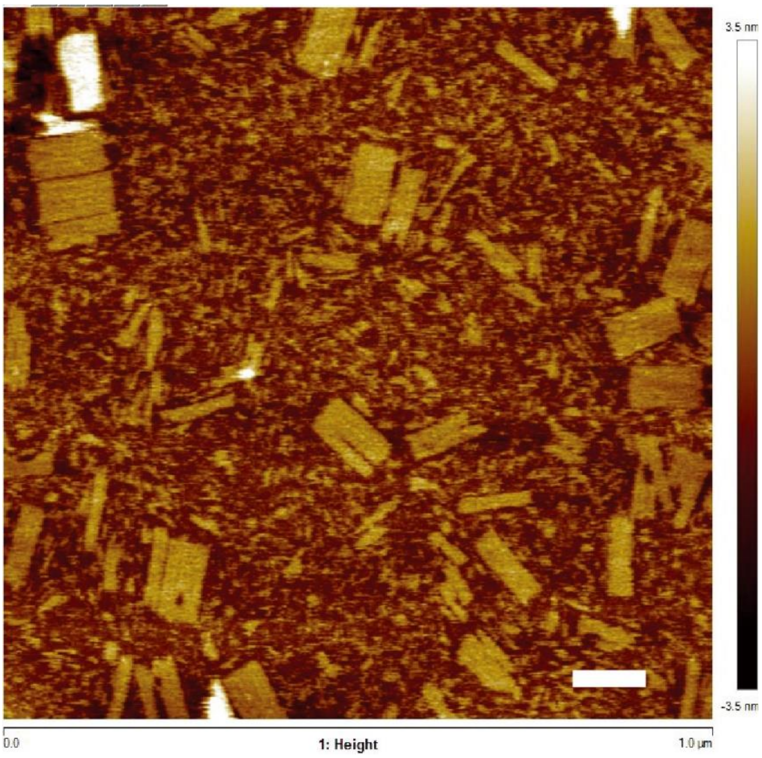

Supplementary Figure 74. AFM result of switch from ON to OFF. Scale bars: 400 nm.

Supplementary Tables

Supplementary Table 1. Number of configurations (N_α) for different toehold lengths (L_t)

L_t	0	1	2	6
N_α	7513	6712	6017	3837

Supplementary Table 2. Propensities for different transition events

Events	Forward Propensity	Backward Propensity
Fraying	$k_{\mathrm{cl}} = 75\times10^6/\mathrm{s}$	$k_{\mathrm{op}} = k_{\mathrm{cl}} \exp(-\Delta G_{bp} / RT)$
Primary binding	$k_{\mathrm{on}} = 3\times10^6/\mathrm{M/s}$	$k_{\mathrm{off}} = k_{\mathrm{on}}[S]/\exp(-\Delta G_{\mathrm{on}} / RT)$<br>$\Delta G_{\mathrm{on}} = \Delta G_{\mathrm{ini}} + RT\ln([S]_0/[S]) - \Delta G_{\mathrm{bp}},\ [S]_0 = 1\mathrm{M}$

*[S] represents for concentration of substrate D_2 or B, $[S]_0$ is the standard concentration.

Supplementary Table 3. Estimated Gibbs free energy in single-dual system

T/°C	ΔG / kcal/mol		
	ΔG_{D}	ΔG_{B1}	ΔG_{B2}
25	-10.75	-7.17	-6.10
40	-10.91	-8.74	-7.82

Supplementary Table 4. Estimated Gibbs free energy for dual pairs of binding partners

T / ˚C	ΔG / kcal/mol	
	ΔG_{D}	ΔG_{B}
25	-8.82	-9.33
40	-7.81	-10.00

Supplementary Table 5. The truth table of the 2-bit input/1-bit output Boolean function f

Input		Output	
System state	Controller N'	New system state	
$s_k(1)$	$c_k(1)$	$s_{k+1}(1)$	Shape
0	0	0	Dimer
0	1	1	Monomer
1	0	1	Monomer
1	1	1	Monomer

Supplementary Table 6. The truth table of the 4-bit input/2 -bit output Boolean function f_1 and f_2

Input				Output		
System state		Controller		New system state		
		N'	*M*'			
$s_k(1)$	$s_k(2)$	$c_k(1)$	$c_k(2)$	$s_{k+1}(1)$	$s_{k+1}(2)$	Shape
0	0	0	0	0	0	Multimer
0	0	0	1	0	1	Dimer
0	0	1	0	1	0	Dimer
0	0	1	1	1	1	Monomer
0	1	0	0	0	1	Dimer
0	1	0	1	0	1	Dimer
0	1	1	0	1	1	Monomer
0	1	1	1	1	1	Monomer
1	0	0	0	1	0	Dimer
1	0	0	1	1	1	Monomer
1	0	1	0	1	0	Dimer
1	0	1	1	1	1	Monomer
1	1	0	0	1	1	Monomer
1	1	0	1	1	1	Monomer
1	1	1	0	1	1	Monomer
1	1	1	1	1	1	Monomer

Supplementary Table 7. The truth table of 12 specific input/output

Input																Output								
System state								Controller								New system state								
								A'	*B'*	*C'*	*D'*	*E'*	*F'*	*G'*	*H'*									
$s_k(1)$	$s_k(2)$	$s_k(3)$	$s_k(4)$	$s_k(5)$	$s_k(6)$	$s_k(7)$	$s_k(8)$	$c_k(1)$	$c_k(2)$	$c_k(3)$	$c_k(4)$	$c_k(5)$	$c_k(6)$	$c_k(7)$	$c_k(8)$	$s_{k+1}(1)$	$s_{k+1}(2)$	$s_{k+1}(3)$	$s_{k+1}(4)$	$s_{k+1}(5)$	$s_{k+1}(6)$	$s_{k+1}(7)$	$s_{k+1}(8)$	Shape
0	0	0	0	0	0	0	0	0	1	1	1	1	1	1	1	0	1	1	1	1	1	1	1	Dimer
0	0	0	0	0	0	0	0	1	0	1	1	1	0	1	1	1	0	1	1	1	0	1	1	Trimer1
0	0	0	0	0	0	0	0	0	1	1	1	1	0	1	1	0	1	1	1	1	0	1	1	Trimer2
0	0	0	0	0	0	0	0	1	0	1	1	1	0	0	1	1	0	1	1	1	0	0	1	I
0	0	0	0	0	0	0	0	1	1	0	0	0	0	1	1	1	1	0	0	0	0	1	1	O
0	0	0	0	0	0	0	0	1	0	1	1	0	0	1	1	1	0	1	1	0	0	1	1	T
0	0	0	0	0	0	0	0	1	0	1	1	0	1	1	0	1	0	1	1	0	1	1	0	J
0	0	0	0	0	0	0	0	0	1	0	1	1	1	0	1	0	1	0	1	1	1	0	1	L
0	0	0	0	0	0	0	0	0	1	1	0	1	0	1	1	0	1	1	0	1	0	1	1	S
0	0	0	0	0	0	0	0	1	1	1	0	1	0	1	0	1	1	1	0	1	0	1	0	Z
0	0	0	0	0	0	0	0	0	0	1	1	0	0	1	1	0	0	1	1	0	0	1	1	Pentamer
0	0	0	0	0	0	0	0	1	0	0	1	0	1	0	1	1	0	0	1	0	1	0	1	Hexamer

Supplementary Table 8. The truth table of tetromino shapes of 16-bit input/8-bit output Boolean function f_3

Input																Output								
System state								Controller								New system state								
								A'	*B'*	*C'*	*D'*	*E'*	*F'*	*G'*	*H'*									
$s_k(1)$	$s_k(2)$	$s_k(3)$	$s_k(4)$	$s_k(5)$	$s_k(6)$	$s_k(7)$	$s_k(8)$	$c_k(1)$	$c_k(2)$	$c_k(3)$	$c_k(4)$	$c_k(5)$	$c_k(6)$	$c_k(7)$	$c_k(8)$	$s_{k+1}(1)$	$s_{k+1}(2)$	$s_{k+1}(3)$	$s_{k+1}(4)$	$s_{k+1}(5)$	$s_{k+1}(6)$	$s_{k+1}(7)$	$s_{k+1}(8)$	Shape
0	0	0	0	0	0	0	0	0	1	0	1	0	1	1	1	0	1	0	1	0	1	1	1	
0	0	0	0	0	0	0	0	0	1	0	1	1	1	1	0	0	1	0	1	1	1	1	0	
0	0	0	0	0	0	0	0	0	1	1	1	0	1	1	0	0	1	1	1	0	1	1	0	
0	0	0	0	0	0	0	0	1	0	1	0	1	0	1	1	1	0	1	0	1	0	1	1	
0	0	0	0	0	0	0	0	1	0	1	0	1	1	0	1	1	0	1	0	1	1	0	1	I
0	0	0	0	0	0	0	0	1	0	1	1	1	0	0	1	1	0	1	1	1	0	0	1	
0	0	0	0	0	0	0	0	1	1	0	1	0	1	1	0	1	1	0	1	0	1	1	0	
0	0	0	0	0	0	0	0	1	1	1	0	1	0	0	1	1	1	1	0	1	0	0	1	
0	0	0	0	0	0	0	0	1	1	0	1	1	0	0	1	1	1	0	1	1	0	0	1	
0	0	0	0	0	0	0	0	1	1	0	1	1	0	1	0	1	1	0	1	1	0	1	0	
0	0	0	0	0	0	0	0	0	1	0	0	1	1	1	1	0	1	0	0	1	1	1	1	
0	0	0	0	0	0	0	0	0	1	1	1	0	0	1	1	0	1	1	1	0	0	1	1	T
0	0	0	0	0	0	0	0	1	0	0	0	1	1	1	1	1	0	0	0	1	1	1	1	
0	0	0	0	0	0	0	0	1	0	1	1	0	1	0	1	1	0	1	1	0	1	0	1	
0	0	0	0	0	0	0	0	1	1	1	0	0	1	0	1	1	1	1	0	0	1	0	1	

0	0	0	0	0	0	0	0	1	1	1	0	0	1	1	0	1	1	1	0	0	1	1	0	
0	0	0	0	0	0	0	0	0	1	1	0	1	1	1	0	0	1	1	0	1	1	1	0	
0	0	0	0	0	0	0	0	0	1	1	1	1	0	1	0	0	1	1	1	1	0	1	0	
0	0	0	0	0	0	0	0	1	0	0	1	1	1	0	1	1	0	0	1	1	1	0	1	
0	0	0	0	0	0	0	0	1	0	1	1	0	1	0	1	1	0	1	1	0	1	0	1	
0	0	0	0	0	0	0	0	1	1	0	0	0	1	1	1	1	1	0	0	0	1	1	1	**O**
0	0	0	0	0	0	0	0	1	1	0	0	1	0	1	1	1	1	0	0	1	0	1	1	
0	0	0	0	0	0	0	0	1	1	0	1	0	0	1	1	1	1	0	1	0	0	1	1	
0	0	0	0	0	0	0	0	1	1	1	0	0	0	1	1	1	1	1	0	0	0	1	1	
0	0	0	0	0	0	0	0	1	1	0	0	0	0	1	1	1	1	0	0	0	0	1	1	
0	0	0	0	0	0	0	0	1	0	0	1	1	1	1	0	1	0	0	1	1	1	1	0	
0	0	0	0	0	0	0	0	1	0	1	0	1	1	1	0	1	0	1	0	1	1	1	0	**J**
0	0	0	0	0	0	0	0	1	0	1	1	0	1	1	0	1	0	1	1	0	1	1	0	
0	0	0	0	0	0	0	0	1	0	1	1	1	0	1	0	1	0	1	1	1	0	1	0	
0	0	0	0	0	0	0	0	0	1	0	1	1	1	0	1	0	1	0	1	1	1	0	1	
0	0	0	0	0	0	0	0	0	1	1	0	1	1	0	1	0	1	1	0	1	1	0	1	
0	0	0	0	0	0	0	0	0	1	1	1	0	1	0	1	0	1	1	1	0	1	0	1	**L**
0	0	0	0	0	0	0	0	0	1	1	1	1	0	0	1	0	1	1	1	1	0	0	1	
0	0	0	0	0	0	0	0	0	1	1	0	1	0	1	1	0	1	1	0	1	0	1	1	
0	0	0	0	0	0	0	0	1	1	0	1	0	1	1	0	1	1	0	1	0	1	1	0	**S**
0	0	0	0	0	0	0	0	1	0	0	1	0	1	1	1	1	0	0	1	0	1	1	1	
0	0	0	0	0	0	0	0	1	1	1	0	1	0	1	0	1	1	1	0	1	0	1	0	**Z**

Supplementary Notes

Supplementary Note 1. Different reaction initial conditions in the proof-of-concept system

In the single-pair system. Pre-reaction initial condition, the preformed duplex D_1 with single binding partner segment *n* (1×, 250 nM) with Cy3 fluorophore at 3'end and duplex D_2 with binding partner segment *n** (1×) mixed with corresponding blocker strand *n*' at gradient concentrations (0×, 1×, 3×, 5×, 25×, 100× and 300×) in 1× TAE buffer supplemented with 15 mM $MgCl_2$. Post-reaction initial condition, the preformed dimer D_1D_2 (1×) was mixed with corresponding blocker strand *n*' at gradient concentrations (0×, 1×, 3×, 5×, 25×, 100× and 300×) in 1× TAE buffer supplemented with 15 mM $MgCl_2$ (Supplementary Figure 1).

In the dual-pair system. Pre-reaction initial condition, the preformed duplex D_1 with two pairs of binding partner segment n_1 and n_2 (1×, 250 nM) with Cy3 fluorophore at 3'end of n_1 and duplex D_2 with binding partner segment n_1* and n_2* (1×) mixed with corresponding blocker strand n_1' and n_2' (Cy5 fluorophore at 3'end) at gradient concentrations (0×, 1×, 3×, 5×, 25×, 100× and 300×) in 1× TAE buffer supplemented with 15 mM $MgCl_2$. Post-reaction initial condition, the preformed dimer D_1D_2 (1×) was mixed with corresponding blocker strand n_1' and n_2' at gradient concentrations (0×, 1×, 3×, 5×, 25×, 100× and 300×) in 1× TAE buffer supplemented with 15 mM $MgCl_2$ (Supplementary Figure 2).

Supplementary Note 2. Probabilistic modelling of DNA base pairing kinetics

Master equations describe a continuous-time Markov process[1], which are used here to model the DNA base pairing kinetics of the toehold-free displacement system. Each state of the model describes the probability of observing the system in a specific configuration as a function of time. The dynamic development of the probability of each discrete state can be described by a set of master equations, which requires the definition of the possible states and their transition rates. The master equations can be solved simultaneously as a set of ordinary differential equations (ODEs) when the number of states is of limited size. However, a Monte Carlo approach such as the stochastic simulation algorithm[1, 2] is needed when the number of states is large, which samples solution trajectories to obtain the statistical characteristics of the probability distribution described by the model. Various models based on master equations have been used for simulating DNA hybridization processes involving the kinetics of DNA breathing and the self-assembly of DNA origami.[3-6]

The nature of DNA strand hybridization is discrete and stochastic on a molecular level. The configuration of a double-helix structure can be classified based on the status of individual nucleotides. Transitions between different configurations are dictated by the kinetics of base pairing, which leads to commonly observed processes such as end fraying and branch migration.[7, 8] We use these concepts here to construct a probabilistic model of the toehold-free displacement system based on master equations, which allows for better understanding of the kinetic pathways that lead to displacement. Furthermore, the novel toehold-free displacement system can be compared to conventional toehold-mediated displacement systems with different toehold lengths to illustrate the importance of toehold-free displacement for the proposed switch. One of the challenges of modeling the kinetics of DNA self-assembly is the potentially large difference in time scales between the various kinetic events. This causes stochastic methods to sample predominantly fast processes such as end fraying (typically every 10^{-6} s) and only rarely the events of interest that change the structural nature of the system such as a new primary binding from solution. Consequently, Monte Carlo

methods are expected to have a high computational load, as long-time scales must be simulated to observe events of interest, yet most of that time would be spend on sampling fast reversible events, which are of little interest. Therefore, we have formulated the model as a set of ODEs and used an implicit variable-step ODE solver to solve instances of the model. The benefit is twofold. First, we can simulate the dynamic development of the probability of each state with error control of an ODE solver. Two, the computational load can be drastically reduced when simulating the time scales of interest. Several assumptions were made to assure that the number of configurations considered was tractable.

The modelled displacement systems are based on the post-assembly experiment. A target strand (D_1) is complementary to either the D_2 strand or blocker (B). D_1 is initially paired to the incumbent D_2 forming a D_1D_2 hybrid, while B acts as the invading strand to displace D_2. To possibly generate a toehold-domain on D_1, D_1 and D_2 are kept at a constant length of 16-nt, while the length of B can be varied within the range 16-nt to 10-nt creating a possible toehold at one end of B. Consequently, D_1 remains fully complementary to D_2 and partially to B if a toehold domain exists. The concentrations of the strands D_1 and D_2 are constant (250 nM), and the concentration of B are varied in the simulations according to the experimental parameters, i.e., as a ratio with respect to D_2 (1×, 3×, 5×, 25×, 100×, 300×). Finally, the temperatures are set at 25℃ or 40℃ to match the experimental conditions in the simulation.

A master equation describes the probability of finding D_1 within a certain configuration α at a given time as follows:

$$\frac{\mathrm{d}P_\alpha}{\mathrm{d}t}(t) = -\sum_{\beta\neq\alpha} \varepsilon_{\beta\leftarrow\alpha} P_\alpha(t) + \sum_{\beta\neq\alpha} \varepsilon_{\alpha\leftarrow\beta} P_\beta(t), \quad \forall t \in (t_0, t_f], \quad P_\alpha(t_0) = P_{\alpha,0}, \qquad (1)$$

where P_α is the probability of finding D_1 in configuration α, $\varepsilon_{\beta\leftarrow\alpha}$ is the propensity factor describing the rate of transition from configuration α to β. Writing Equation (1) for all configurations leads to

$$\frac{\mathrm{d}\mathbf{P}}{\mathrm{d}t}(t) = \boldsymbol{\varepsilon}\mathbf{P}(t), \quad \forall t \in (t_0, t_f], \quad \mathbf{P}(t_0) = \mathbf{P}_0, \qquad (2)$$

where **P** is the state vector consisting of the probabilities of every configuration and $\boldsymbol{\varepsilon}$ is a $(N_\alpha \times N_\alpha)$ propensity matrix with elements as follows:

$$\boldsymbol{\varepsilon}_{\alpha\beta} = \varepsilon_{\alpha\leftarrow\beta} \text{ if } \alpha \neq \beta, \quad \boldsymbol{\varepsilon}_{\alpha\alpha} = -\sum_{\beta\neq\alpha} \varepsilon_{\beta\leftarrow\alpha},$$

where N_α is the number of total configurations.

The number of states in the model (N_α) will be large when considering either hybridization with D_2 or B at each position (i.e., $N_\alpha = 3^{16}$), which would complicate the proposed direct solution procedure. Therefore, model reduction is applied. We assume that primary binding of strands B or D_2 from solution only occur at the vacant and correct positions of D_1 (Supplementary Figure 15.a). Furthermore, at most one strand of D_2 and B can be hybridized to the target strand, i.e., the impact of any competition between the same type of strand or displacement from a third one is neglected (Supplementary Figure 15.b). Finally, fraying can only occur from the edges of a double strand domain (Supplementary Figure 15.c). The impact of any DNA breathing is neglected, which is justified by the short length of the strands in our system. To systematically define all possible states under the given assumptions, we represent each configuration of D_1 with length *l* by $\mathrm{D}_{n_1,n_2,\ldots,n_i,\ldots,n_l}$ for a toehold-free case, where the indices $n_i \in \{0,1,2\}$ indicate the state of each base pair *i* on D_1, i.e., vacant ('0'), paired

to D_2 (‘1’), paired to B (‘2’). An algorithm has been developed to generate all possible configurations under the given assumptions. A matrix **X** with dimension $N_\alpha \times 16$ is defined to store the indices of each configuration of D_1 (i.e., $D_{n_1,n_2,\ldots,n_i,\ldots,n_l}$), and a matrix **I** with dimension $N_\alpha \times 4$ is used for the determination of the connections between configurations. The number of resulting configurations as function of toehold length (L_t) is listed in Supplementary Table 1. The number of configurations translate to a set of ODEs that is sufficiently small to solve instances of the model with standard numerical ODE solvers.

ALGORITHM FOR IDENTIFICATION OF ALL CONFIGURATIONS

Set l = 16 # length of D_1

Set $L_t \in \{0, 1, 2, 6\}$ # length of toehold domain

Set $l_d \in [0, 16]$ # length of D_2 domain

Set $d_1 \in [1, l]$ # first position of D_2 domain

Set $d_2 \in [1, l]$ # final position of D_2 domain

Set $l_b \in [0, l - L_t]$ # length of B domain

Set $b_1 \in [L_t + 1, l]$ # first position of B domain

Set $b_2 \in [L_t + 1, l]$ # final position of B domain

Set $\mathbf{X} \in \mathbf{0}^{N_\alpha \times 16}$ # matrix for storing all the configuration of D_1, initialized as a zero matrix

Set $\mathbf{I} \in \mathbb{Z}^{N_\alpha \times 4}$ # matrix for storing the information of a given configuration. Elements [l_d, d_1, l_b, b_1] represent length and first position of D_2, length and first position of B

```
# Generation of configuration
Set icount = 1
Set X(1,:) = 0^(1×16)
Set I(1,:) = [0 0 0 0]
# Generation of configurations
WHILE l_d <= l
        IF l_d == 0 THEN # only B domain exist
              # determine all the possible positions of B
              # B begins at the first possible position on D_1 with length of 1-nt
              Set l_b = 1
              Set b_1 = L_t + 1
              WHILE l_b <= l – L_t
                   b_2 = b_1 + l_b – 1 # calculation of the end of the B domain
                   IF b_2 <= l THEN
                        icount = icount + 1
                        X(icount, b_1:b_2) = 2 # store the configuration
                        I(icount) = [0 0 l_b b_1] # store the position of B
                        IF b_2 < l THEN
                             # keep l_b and shift the position of B with 1-nt
                             b_1 = b_1 + 1
                        ELSEIF b_2 == l THEN
                             # extend the length of B with 1-nt
                             # shift the first position of B to L_t + 1
                             b_1 = L_t + 1
```

```
            lb = lb + 1
          END IF
        END IF
      END WHILE
    ELSEIF ld > 0 THEN
      # determine possible position of D2
      # D2 begins at the first position of D1 with length of 1-nt
      Set ld = 1
      Set d1 = 1
      WHILE ld <= l
        d2 = d1 + ld – 1 # calculation of the end of D2 domain
        IF d2 <= l THEN
          icount = icount + 1
          X(icount, d1:d2) = 1
          I(icount) = [ld d1 0 0]
          # for the given D2 domain, determine B domain
          # begin with B at the first available position with 1-nt length
          Set lb = 1
          Set b1 = the first '0' position at domain [Lt + 1, l]
          WHILE b <= max{d1 – Lt, l – d2}
            b2 = b1 + lb – 1 # calculation of the end of B domain
            IF {b1, b2|b1<b2<d1} or {b1, b2|b1>d2, b2<=l} THEN
              # B domain exists at the one side of D2 domain
              icount = icount + 1
              X(icount, d1:d2) = 1
              X(icount, b1:b2) = 2
              I(icount) = [ld, d1, lb, b1]
              IF b2 < d1−1 or b2 < l THEN
                # keep lb and shift B with 1-nt
                b1 = b1 + 1
              ELSEIF b2 == d1−1 or b2 == l THEN
                # extend the length of B with 1nt
                # begin from the first position of B
                lb = lb + 1
                b1 = the first '0' position at [Lt+1, l]
              END IF
            END IF
          END WHILE
          IF d2 < l THEN
            # keep length and shift D2 domain with 1-nt
            d1 = d1 + 1
          ELSEIF d2 == l THEN
            # extend D2 with 1-nt and begin from the first position
            ld = ld + 1
            d1 = 1
          END IF
        END IF
      END WHILE
    END IF
END WHILE
```

To construct the propensity matrix, two types of events are considered, i.e., primary binding of D_2 or B from solution, edge fraying of a double-strand domain (illustrated in Supplementary Figure 16.). The possible transitions for a given configuration are stored by searching the matrix **I** with specific conditions. For example, to identify the possible transitions of a D_1 configuration ($[l_d, d_1, 0, 0]$) by primary binding from a B strand, the searching condition will be: (1) length and position (l_d, d_1) of D_2 of the given configuration; (2) one base pair of D_1 binding to blocker (l_b=1). To identify the transitions by edge fraying of D_2 domain of a given configuration ($[l_d, d_1, l_b, b_1]$), searching conditions will be: (1) $[l_d -1, d_1+1, l_b, b_1]$ for the unpairing of first position; (2) $[l_d +1, d_1-1, l_b, b_1]$ for the pairing on neighboring position of the first position; (3) $[l_d -1, d_1, l_b, b_1]$ for the unpairing of final position; (4) $[l_d +1, d_1, l_b, b_1]$ for the pairing on neighboring position of the final position. Propensities based on the base paring kinetics are calculated for the transitions between two states to construct the propensity matrix with sparsity pattern as shown in Supplementary Figure 17. The free energy change for the pairing of DNA strands is obtained from the nearest-neighbor thermodynamics model[9],

$$\Delta G = \Delta G_{\text{ini}} + \sum \Delta G_{NN} \qquad (3)$$

where ΔG_{ini} is the free energy change for primary binding, ΔG_{NN} is the free energy change for neighboring pairs. In our approach, ΔG_{bp} for base pairing is defined as the averaged value of ΔG_{NN} at a given temperature. Therefore, ΔG for the pairing of strands will be $\Delta G_{\text{ini}}+(l\text{-}1)\ \Delta G_{\text{bp}}$. The reaction kinetic constants for pairing steps (k_{on} and k_{cl}) are obtained from the literature[7, 8], and kinetic constants for unpairing steps are calculated based on the ΔG of given events. The propensities for the different events are shown in Supplementary Table 2.

The master equations (Eqn. 2) for systems with 4 types of toehold domain were numerically solved by the variable-step, variable-order ODE solver *ode15s* in Matlab (The MathWorks, Inc.). The probability of observing D_1 in a dimer configuration, i.e., with at least one base pair connected to D_2, is shown in Supplementary Figure 18. These model simulations predict that the reversibility of the displacement system largely vanishes with an increasing toehold domain length. For instance, a 50% probability of observing the system in a dimer configuration corresponds to a concentration ratio of B to D_2 of 1× for the toehold-free strand displacement system, which increases to 100× when there is a toehold of 2-nt. The nearest-neighbor thermodynamic model predicts that the difference of ΔG for the formation of BD_1 and D_1D_2 is a function of the toehold length (L_t) and temperature, which is reflected by the gap of probabilities for the two tested temperatures for different toehold lengths ($L_t > 0$). While for $L_t = 0$, an identical ΔG is calculated from our model since D_2 and B share the same base sequences, therefore, the same equilibrium probability distributions are obtained regardless of the temperature difference. The dynamic trajectories of the probabilities for the displacement process (Supplementary Figure 19.) demonstrate that the system reaches equilibrium within hundreds of seconds, which is consistent with the experimental observations (Supplementary Figure 20.). This suggests that the duration of the experiments of one hour was enough to reach the equilibrium state of the displacement system.

Supplementary Note 3. Second-order reaction model of the proof-of-concept single-pair system

The reversible elementary reactions in the proof-of concept displacement system with a single pair of binding partners and their Gibbs free energy change are described by:

$$\mathrm{D_1 + D_2 \rightleftharpoons D_1D_2},\ \Delta G_{\mathrm{D}}, \quad \text{(R1)}$$

$$\mathrm{D_1 + B_1 \rightleftharpoons B_1D_1},\ \Delta G_{\mathrm{B1}}, \quad \text{(R2)}$$

$$\mathrm{D_1 + B_2 \rightleftharpoons B_2D_1},\ \Delta G_{\mathrm{B2}}, \quad \text{(R3)}$$

where D_1 and D_2 are the duplexes. B_1 is the blocker fully complementary to D_1, B_2 is a blocker that is 1-nt shorter than D_1. D_1D_2 is the dimer structure (defined as 'ON' state), B_1D_1 and B_2D_1 are the blocked D_1 structures (defined as 'OFF' state). The combination of the dimerization (R1) and blocking (R2) reactions on D_1 creates a toehold-free strand displacement (TFSD) system, as D_2 and B_1 are of identical length and have the same base pair sequence. In contrast, when combining the dimerization reaction (R1) with the blocking reaction (R3), a toehold-mediated strand displacement (TMSD) system is created due to the toehold domain on B_2D_1 resulting from the difference in length between B_2 and D_1. ΔG_{D}, ΔG_{B1} and ΔG_{B2} are the Gibbs free energy change for the dimerization and blocking reactions on a single sticky end. At equilibrium, the concentrations of the species are described by the reaction equilibrium constants as follows,

$$\frac{C_{\mathrm{D_1D_2}}}{C_{\mathrm{D_1}}C_{\mathrm{D_2}}} = K_{\mathrm{D}}, \quad (4)$$

$$\frac{C_{\mathrm{B_1D_1}}}{C_{\mathrm{D_1}}C_{\mathrm{B_1}}} = K_{\mathrm{B1}}, \quad (5)$$

$$\frac{C_{\mathrm{B_2D_1}}}{C_{\mathrm{D_1}}C_{\mathrm{B_2}}} = K_{\mathrm{B2}}, \quad (6)$$

where $C_{\sim}$ is the equilibrium concentration of a given species in solution. K_{D}, K_{B1} and K_{B2} are the equilibrium constants for dimerization and blocking reactions, respectively, which can be obtained from the corresponding ΔG as follows:

$$K_{\mathrm{D}} = \exp(-\Delta G_{\mathrm{D}} / RT), \quad (7)$$

$$K_{\mathrm{B1}} = \exp(-\Delta G_{\mathrm{B1}} / RT), \quad (8)$$

$$K_{\mathrm{B2}} = \exp(-\Delta G_{\mathrm{B2}} / RT), \quad (9)$$

where T is the temperature and R is the ideal gas constant. Material balances for the species in the displacement systems complete the model,

$$C_{\mathrm{D_1},0} = C_{\mathrm{D_1}} + C_{\mathrm{D_1D_2}} + C_{\mathrm{B_1D_1}} + C_{\mathrm{B_2D_1}}, \quad (10)$$

$$C_{\mathrm{D_2},0} = C_{\mathrm{D_2}} + C_{\mathrm{D_1D_2}}, \quad (11)$$

$$C_{\mathrm{B_1},0} = C_{\mathrm{B_1}} + C_{\mathrm{B_1D_1}}, \quad (12)$$

$$C_{\mathrm{B_2},0} = C_{\mathrm{B_2}} + C_{\mathrm{B_2D_1}}, \quad (13)$$

where $C_{\sim,0}$ is the initial concentration of a given species.

Supplementary Note 4. Parameter estimation of Δ*G* in single-pair system

For the analytical calculation of the concentrations of the target species at a given temperature, we assume that the reaction systems only have one type of blocker at the same time, which is consistent with the experiments, i.e., the concentration of either B_1 or B_2 is equal to zero. Therefore, C_{B} represents the concentration of either B_1 or B_2, whichever is nonzero. The equilibrium constants K_{D}, K_{B1} and K_{B2} are obtained from equations 7 to 9 for a given temperature T. By substitution of equation 4 into 11, the concentration of D_2 as a function of the concentration of D_1 is obtained,

$$C_{D_2} = \frac{C_{D_2,0}}{1 + K_D C_{D_1}}. \quad (14)$$

Substitution of 5 and 6 into 12 and 13 leads to an expression for the concentration of B as follows:

$$C_B = \frac{C_{B,0}}{1 + K_B C_{D_1}}. \quad (15)$$

From substitution of equation 11, 12 and 13 into 10, expression of C_{D_1} is derived as

$$C_{D_1} = C_{D_1,0} - C_{D_2,0} - C_{B,0} + C_{D_2} + C_B, \quad (16)$$

Given that for our experimental conditions, the initial concentration of D_1 is equal to D_2 (i.e., $C_{D_1,0} = C_{D_2,0}$). Therefore, by substituting equation 14 and 15 into 16, an implicit expression in which the concentration of D_1 is the only unknown can be obtained,

$$K_D K_B C_{D_1}^3 + (K_D + K_B + K_D K_B C_{B,0}) C_{D_1}^2 + (1 + K_B (C_{B,0} - C_{D_1,0})) C_{D_1} - C_{D_1,0} = 0. \quad (16)$$

The coefficients of equation 16 compared to the standard cubic equation $ax^3+bx^2+cx+d=0$ are

$$a = K_D K_B,$$
$$b = K_D + K_B + K_D K_B C_{B,0},$$
$$c = 1 + K_B (C_{B,0} - C_{D_1,0}),$$
$$d = -C_{D_1,0},$$

Only the solution of equation 16 within the concentration range of D_1 ($0 < C_{D_1} < C_{D_1,0}$) is feasible. The concentrations for the other species are obtained by the substitution of C_{D_1} into equations 4 to 6, 14 and 15. All equations were implemented in Matlab (The MathWorks, Inc. version: R2020b Update 4 9.9.0.1570001).

The equilibrium state of the displacement system at a given temperature is only governed by the initial concentrations of D_1, D_2, B_1 and B_2, and the ΔG for the elementary reactions. We aim to fit the experimental data involving the fractions of different species (i.e., D_1 D_2, B_1D_1, B_2D_1 and D_2, fractions of given species in S1) at different initial blocker concentrations to the model presented in this section by estimating the values of ΔG_D, ΔG_{B1} and ΔG_{B2} from the following optimization problem (P1) based on maximum likelihood estimation:

$$\min_{\substack{\Delta G_D \in (-30,0) \\ \Delta G_{B1} \in (-30,0) \\ \Delta G_{B2} \in (-30,0)}} \Phi = \sum_{i=1}^{N_r} \sum_{j=1}^{M} \left(\frac{\left(\tilde{F}_{D_1D_2,ij} - F_{D_1D_2,i}\right)^2}{\sigma_{D_1D_2,i}^2} + \frac{\left(\tilde{F}_{B_1D_1,ij} - F_{B_1D_1,i}\right)^2}{\sigma_{B_1D_1,i}^2} + \frac{\left(\tilde{F}_{B_2D_1,ij} - F_{B_2D_1,i}\right)^2}{\sigma_{B_2D_1,i}^2} + \frac{\left(\tilde{F}_{D_2,ij} - F_{D_2,i}\right)^2}{\sigma_i^2} \right)$$

s.t. equation 4 to 9, 14, 15, 16

$$C_{D_1,0} = 250 \text{ nM}$$
$$C_{D_2,0} = 250 \text{ nM}$$
$$C_{B_1,0} = 250 \text{ nM} \times N$$
$$C_{B_2,0} = 250 \text{ nM} \times N \quad \text{(P1)}$$
$$N \in \{0,1,3,5,25,100,300\},$$

$$F_{\mathrm{D_1D_2}} = C_{\mathrm{D_1D_2}} / (C_{\mathrm{D_1D_2}} + C_{\mathrm{B_1D_1}} + C_{\mathrm{B_2D_1}} + C_{\mathrm{D_2}}),$$
$$F_{\mathrm{B_1D_1}} = C_{\mathrm{B_1D_1}} / (C_{\mathrm{D_1D_2}} + C_{\mathrm{B_1D_1}} + C_{\mathrm{B_2D_1}} + C_{\mathrm{D_2}}),$$
$$F_{\mathrm{B_2D_1}} = C_{\mathrm{B_2D_1}} / (C_{\mathrm{D_1D_2}} + C_{\mathrm{B_1D_1}} + C_{\mathrm{B_2D_1}} + C_{\mathrm{D_2}}),$$
$$F_{\mathrm{D_2}} = C_{\mathrm{D_2}} / (C_{\mathrm{D_1D_2}} + C_{\mathrm{B_1D_1}} + C_{\mathrm{B_2D_1}} + C_{\mathrm{D_2}}),$$

where N_r is the total number of tested ratios of the blocker concentration to the D2 concentration, and M is the total number of measurements for each experiment. $\widetilde{F}_{\sim,ij}$ is the measured fraction for a given species from experiments, and $F_{\sim,i}$ is the fraction predicted from the model at the same conditions. $\sigma^2_{\sim,i}$ is the variance of experimental data. The value of the variance is replaced with an averaged variance of the measurements of other species from the same experiment in case the variance is equal to zero. The lower bound for ΔG of -30kcal/mol is chosen sufficiently low compared to the value predicted from the nearest-neighbor thermodynamics model[9] (around -22kcal/mol) to allow for realistic solutions while avoiding potential numerical complications resulting from an unrestricted search space. Optimization problem P1 was implemented in Matlab and was solved with the fmincon solver based on the experimental results from two temperatures, i.e., 25°C and 40°C.

The experimental and simulation results are illustrated in Supplementary Figure 21. The model simulations consistently describe the decreasing trends of dimer fractions with the increasing blocker concentrations. The model simulations well fit the fractions of D_1D_2, B_1D_1 and D_2 in the TFSD system, while lower fractions of D_1D_2 and higher fractions of D_2 from TMSD system when N < 25 are obtained from the reaction model. The TFSD and TMSD share same strands with different length of blocker strand, so the dimer fractions from experiments without blocker should be identical. But an increment of fractions of D_1D_2 and a decrement of fractions of D_2 at $N = 0$ from TMSD compared to the TFSD were obtained from the experiment results, which is unexpected. Such differences of fractions could attribute to unreplicated experiment of TMSD. Our reaction model only involves ΔG and concentrations of species as variables, therefore, it could not capture the differences from unexpected effect.

The estimated values for ΔG_{D}, ΔG_{B1}, ΔG_{B2} at different temperatures are listed in Supplementary Table 3. The estimated ΔG_{D}, ΔG_{B1} and ΔG_{B2} are higher than those predicted from the nearest-neighbor thermodynamics model, which could point to a reduced entropy change during the pairing reaction of binding partner of D_1 compared to a standard duplex formation. Focusing on the ΔG_{D}, ΔG_{B1} from TFSD, the pairing of D_1 by D_2 appears more stable than pairing with a blocker at 25°C and 40°C, as the estimated ΔG_{D} is lower than ΔG_{B1}. The reason why dimer is more stable could be some internal interactions (e.g., Van der Waals forces) between two duplexes. The effect of temperature on the dimerization process is not substantial based on our model simulations, as the experimental fractions without blockers at 25°C and 40°C do not differ substantially. ΔG_{B1} decreases with increasing temperature suggesting that B_1D_1 is more stable at a higher tested temperature, which could be resulted from some unexpected effects. For instance, a higher temperature will promote the entanglement of the poly-T domain of binding partner of B_1D_1 with the duplex, which could stabilize the blocked duplex. Such entanglement is difficult to occur in a dimer structure as the binding partner connected to two duplexes is less flexible. Therefore, a low temperature favors the 'ON' state.

Supplementary Note 5. Second-order reaction model of the proof-of-concept dual-

pair system

In the dual-pair system, two types of blockers are introduced to complement the dual binding partners. The binding partners and blockers all share the same length; therefore, the displacement process is toehold-free. We assume that the paring of any pair of binding partners involves identical free energies (ΔG_D), and blocking of any binding partner involves identical ΔG_B. The dual-pair system can be described by four reaction equations with corresponding free-energy changes as follows:

$$D_I + D_{II} \rightleftharpoons D_I D_{II},\ 2\Delta G_D, \quad (R4)$$

$$D_I + D_{II} + B_I \rightleftharpoons D_I B_I D_{II},\ \Delta G_D + \Delta G_B, \quad (R5)$$

$$D_I + D_{II} + B_{II} \rightleftharpoons D_I B_{II} D_{II},\ \Delta G_D + \Delta G_B, \quad (R6)$$

$$D_I + B_I + B_{II} \rightleftharpoons D_I B_I B_{II},\ 2\Delta G_B, \quad (R7)$$

where D_I and D_{II} are the duplexes, B_I and B_{II} are the blockers fully complementary to the corresponding sticky ends on D_I. $D_I D_{II}$ is the dimer structure (defined as 'ON' state), and $D_I B_I B_{II}$ is the fully blocked structure ('OFF' state). $D_I B_I D_{II}$ and $D_I B_{II} D_{II}$ are the intermediate structures with one of the binding partners blocked and the other connected to D_{II}. The equilibrium concentrations of the reactions are described by:

$$\frac{C_{D_I D_{II}}}{C_{D_I} C_{D_{II}}} = K_D{}^2, \quad (17)$$

$$\frac{C_{D_I B_I D_{II}}}{C_{D_I} C_{D_{II}} C_{B_I}} = K_D K_B, \quad (18)$$

$$\frac{C_{D_I B_{II} D_{II}}}{C_{D_I} C_{D_{II}} C_{B_{II}}} = K_D K_B, \quad (19)$$

$$\frac{C_{D_I B_I B_{II}}}{C_{D_I} C_{B_I} C_{B_{II}}} = K_B{}^2, \quad (20)$$

where $C_{\sim}$ is the equilibrium concentration of a given species. K_D and K_B are the equilibrium constants described by:

$$K_D = \exp(-\Delta G_D / RT), \quad (21)$$

$$K_B = \exp(-\Delta G_B / RT), \quad (22)$$

The material balances of D_I, D_{II} and both blockers complete the model:

$$C_{D_I,0} = C_{D_I} + C_{D_I D_{II}} + C_{D_I B_I D_{II}} + C_{D_I B_{II} D_{II}} + C_{D_I B_I B_{II}}, \quad (23)$$

$$C_{D_{II},0} = C_{D_{II}} + C_{D_I D_{II}} + C_{D_I B_I D_{II}} + C_{D_I B_{II} D_{II}}, \quad (24)$$

$$C_{B_I,0} = C_{B_I} + C_{D_I B_I D_{II}} + C_{D_I B_I B_{II}}, \quad (25)$$

$$C_{B_{II},0} = C_{B_{II}} + C_{D_I B_{II} D_{II}} + C_{D_I B_I B_{II}}, \quad (26)$$

where $C_{\sim,0}$ is the initial concentration of a given species.

Supplementary Note 6. Parameter estimation of ΔG in dual-pair system

The model has an explicit solution for the concentration of D_I, which can be derived as follows. Since B_I and B_{II} share identical total concentration ($C_{B,0}$), it can be shown that

$$C_{B_I} = C_{B_{II}} = C_B. \quad (27)$$

We assume that the concentrations of the intermediate species (i.e., $D_I B_I D_{II}$ and $D_I B_{II} D_{II}$) are negligibly small compared to the concentrations of the dimer structure and the fully

blocked species. Under this assumption, the model can be reduced to two reaction equations, R4 and R7, and only the equilibrium (17 and 20) will be involved. The mass balances are reduced as below

$$C_{D_I,0} = C_{D_I} + C_{D_I D_{II}} + C_{D_I B_I B_{II}}\,, \qquad (28)$$

$$C_{D_{II},0} = C_{D_{II}} + C_{D_I D_{II}}\,, \qquad (29)$$

$$C_{B,0} = C_B + C_{D_I B_I B_{II}}\,. \qquad (30)$$

By combining equations 28 to 30 with the experiment condition that D_I and D_{II} share identical total concentration, the material balance of D_I can be written as:

$$C_{D_I} - C_{D_{II}} - C_{D_I B_I B_{II}} + C_{B,0} = 0\,. \qquad (31)$$

Substitution of 17 into 3.2.13 and 20 into 30 gives the concentration of D_{II} and B as function of the concentration of D_I,

$$C_{D_{II}} = \frac{C_{D_{II},0}}{K_D^2 C_{D_I} + 1}\,, \qquad (32)$$

$$K_B{}^2 C_{D_I} C_B^2 + C_B - C_{B,0} = 0\,. \qquad (33)$$

Note that C_B should be nonnegative value. Therefore, the only feasible solution of quadratic equation 33 is given by:

$$C_B = \frac{\sqrt{4K_B{}^2 C_{B,0} C_{D_I} + 1} - 1}{2K_B{}^2 C_{D_I}}\,. \qquad (34)$$

An approximation to the square root by continued fraction is applied:

$$\sqrt{x^2 + y} \approx x + \cfrac{y}{2x + \cfrac{y}{2x + \ldots}}\,. \qquad (35)$$

We use the second iteration of 35 to obtain:

$$\sqrt{4K_B{}^2 C_{B,0} C_{D_I} + 1} \approx 1 + \frac{4K_B{}^2 C_{B,0} C_{D_I}}{2 + 4K_B{}^2 C_{B,0} C_{D_I} / 2}\,, \qquad (36)$$

The approximation will result in a 5% difference between the LHS and RHS of 36 by substituting the obtained C_{D_I} from equation 38. The concentration of the blockers as a function of concentration of D_I is given by

$$C_B = \frac{C_{B,0}}{1 + K_B{}^2 C_{B,0} C_{D_I}}\,, \qquad (37)$$

Substitution of 32 and 37 into 31 leads to the equation 38 for the concentration of D_I,

$$C_{D_I} - \frac{C_{D_{II},0}}{K_D^2 C_{D_I} + 1} - \frac{C_{B,0}}{1 + K_B{}^2 C_{B,0} C_{D_I}} + C_{B,0} = 0\,, \qquad (38)$$

Equation 38 is a cubic equation and can be solved explicitly. The concentrations of the other species are obtained by substituting the concentration of D_I into equation 32, 34, 27 and 29.

Estimation of ΔG_D and ΔG_B has been done by solving the optimization problem P2 to fit the model to the experimental fractions of the identified species from PAGE assay (i.e., $D_I D_{II}$, $D_I B_I B_{II}$, and D_{II}) at the tested temperatures (i.e., 25ºC or 40ºC):

$$\min_{\substack{\Delta G_D \in [-30,0] \\ \Delta G_B \in [-30,0]}} \Phi = \sum_{i=1}^{N_r} \sum_{j=1}^{M} \alpha_i \left(\frac{\left(\tilde{F}_{D_I D_{II},ij} - F_{D_I D_{II},i}\right)^2}{\sigma^2_{D_I D_{II},i}} + \frac{\left(\tilde{F}_{D_I B_I B_{II},ij} - F_{D_I B_I B_{II},i}\right)^2}{\sigma^2_{D_I B_I B_{II},i}} + \frac{\left(\tilde{F}_{D_{II},ij} - F_{D_{II},i}\right)^2}{\sigma^2_{D_{II},i}} \right)$$

s.t. equation 27 to 29, 32, 34, 38,

$$C_{\mathrm{D_I},0} = 250\ \mathrm{nM},$$
$$C_{\mathrm{D_{II}},0} = 250\ \mathrm{nM},$$
$$C_{\mathrm{B},0} = 250\ \mathrm{nM} \times N, \qquad \text{(P2)}$$
$$N \in \{0,1,3,5,25,100,300\},$$
$$F_{\mathrm{D_I D_{II}}} = C_{\mathrm{D_I D_{II}}} / (C_{\mathrm{D_I D_{II}}} + C_{\mathrm{D_I B_I B_{II}}} + C_{\mathrm{D_{II}}}),$$
$$F_{\mathrm{D_I B_I B_{II}}} = C_{\mathrm{D_I B_I B_{II}}} / (C_{\mathrm{D_I D_{II}}} + C_{\mathrm{D_I B_I B_{II}}} + C_{\mathrm{D_{II}}}),$$
$$F_{\mathrm{D_{II}}} = C_{\mathrm{D_{II}}} / (C_{\mathrm{D_I D_{II}}} + C_{\mathrm{D_I B_I B_{II}}} + C_{\mathrm{D_{II}}}),$$

where α is a weighing factor for improving the quality of the fit at higher blocker concentrations, as the experimental results of dimer fractions are around 100% at low concentrations of blockers (N < 25). We used two sets of α for two temperatures, $\alpha \in \{1,1,1,1,10,100,1000\}$ for 40°C and $\alpha \in \{0,0,0,1,10,100,1000\}$ for 25°C.

Optimization problem P2 was solved with the same method as was used for P1. The experimental and simulation results are shown in Supplementary Figure 22. The experimental trend of decreasing fractions of $\mathrm{D_I D_{II}}$ only when N > 5 at both temperatures is captured well by the model. The fractions of the intermediate structures ($\mathrm{D_I B_I D_{II}}$ and $\mathrm{D_I B_{II} D_{II}}$) as predicted by the model are shown in Supplementary Figure 23. The assumption of a negligibly small concentration of intermediates is valid in the final solution, as the concentrations, obtained from equations 18 and 19, are less than 1% of the concentration of $\mathrm{D_I D_{II}}$ and $\mathrm{D_I B_I B_{II}}$. These structures were also not observed from the gel electrophoresis. The small fraction of intermediate structures and blocker concentration threshold are expected as the results from the cooperativity of binding partners. Comparing the system with single and dual pairs of binding partners (eqn. 3.1.1 to 3.1.3, eqn. 17 to 20), the formation of dimer and blocked species with n_{P} pairs of binding partners can be derived,

$$C_{\mathrm{D_I D_{II}}} = K_{\mathrm{D}}^{n_p} C_{\mathrm{D_I}} C_{\mathrm{D_{II}}}, \qquad (39)$$
$$C_{\mathrm{BD_I}} = K_{\mathrm{B}}^{n_p} C_{\mathrm{D_I}} C_{\mathrm{B}}^{n_p}, \qquad (40)$$
$$\frac{C_{\mathrm{D_I D_{II}}}}{C_{\mathrm{BD_I}}} = \frac{K_{\mathrm{D}}^{n_p} C_{\mathrm{D_{II}}}}{K_{\mathrm{B}}^{n_p} C_{\mathrm{B}}^{n_p}}, \qquad (41)$$

where $\mathrm{BD_I}$ represents fully blocked species. Noting that equation 40 and 41 are under the condition that all the blockers share identical total concentration. In case of the total concentration $C_{\mathrm{B},0}$ < 1M, increasing binding partners will reduce the magnitude of $C_{\mathrm{B}}^{n_p}$. Therefore, when the total concentrations of blocker and $\mathrm{D_{II}}$ are close (e.g., N < 5 in the dual-pair experiment), $C_{\mathrm{D_{II}}}$ is substantially larger than $C_{\mathrm{B}}^{n_p}$, which is the reason why $\mathrm{D_I D_{II}}$ is the dominant structure when N < 5. The threshold of blocker concentration is the concentration which shifts the equilibrium composition from dimer structure to blocked species. Such shifting of equilibrium composition needs to be identified by PAGE separation.

The estimated values for the free energy are listed in Supplementary Table 4. Opposite to the findings from the single-pair system, ΔG_{B} is smaller than ΔG_{D}, which is consistent with a blocked state that is more stable than the dimer. This difference could be resulted from an entropy penalty from the internal loop formation of the dimer with dual binding partners.

To conclude, increasing binding partners on the duplexes will raise the requirement of concentration of blockers to obtain a switch between ON and OFF states. OFF state will be more stable with the increased pairs of binding partners. In the case of sufficient concentration of blocker, switch from ON to OFF state will be benefit from multiple binding partners.

Supplementary Note 7. Reactions for ON/OFF switch from pre-reaction state

The preformed origami rectangle I with connection staples *N* (1×, 1.25 nM) and origami rectangle II with connection staples *N** (1×) after purification were mixed with corresponding blocker set *N*' at gradient concentrations (0×, 1×, 3×, 5×, 25×, 100× and 300×) in 1× TAE buffer supplemented with 15 mM $MgCl_2$. Samples were subjected to an isothermal incubation (40°C for 40 hours) before native agarose gel electrophoresis.

Supplementary Note 8. Reactions for switch from ON state to OFF state

The preformed origami rectangles I and II without purification were directly mixed and subjected to an isothermal annealing program (40°C for 40 hours) before native agarose gel electrophoresis purification. The purified dimers (ON state) were turned to be switched to monomers (OFF state) with blocker set *N*' at gradient concentrations (0×, 1×, 3×, 5×, 25×, 100× and 300×) in an isothermal incubation (48.6°C for 40 hours). Samples after displacement reaction were subjected to native agarose gel electrophoresis.

Supplementary Note 9. Reactions for the 2-bit input/1-bit output system

The preformed origami cuboid I with sticky face *N* (1×, 1.25nM) and origami cuboid II with sticky face *N** (1×) after purification were mixed with corresponding blocker set *N*' at gradient concentrations (0×, 1×, 3×, 5×, 25× and 100×) in 0.5× TAE buffer supplemented with 35 mM $MgCl_2$. Samples were subjected to an isothermal incubation (42°C for 14 hours) before native agarose gel electrophoresis.

Supplementary Note 10. Reactions for the 4-bit input/2-bit output system

The preformed origami cuboid I (with sticky faces *N* and *M*) and origami cuboid II (with sticky faces *N** and *M**) after purification were mixed with corresponding blocker sets *N*' and *M*' at an excess concentration in 0.5× TAE buffer supplemented with 35 mM $MgCl_2$. Samples were subjected to an isothermal incubation (42°C for 14 hours) before native agarose gel electrophoresis.

Supplementary Note 11. Definition and the MIMO Boolean functions of a dual-unit system of origami cuboids

The Boolean function contains inputs (system state and controller) and output (new system state). Binary variables (0/1) of the system state are sticky faces, and that of the controller are blockers. We use 0 to represent sticky face not being blocked or blockers not being added. And we use 1 to represent sticky face being blocked or blockers being added with an excess amount. Boolean function formulation may describe iterative computations.

In the manuscript's Fig. 3(a) the Boolean function of 2-bit input and 1-bit output is described. Let $s_k = s_k(1)$ be the system state at the k-th iteration, where $s_k(1)$ represents whether *N* is blocked by *N*'. Let $c_k = c_k(1)$ be the controller at the k-th iteration, where $c_k(1)$ represents whether blocker *N*' is added at an excess amount. Then $s_{k+1} = s_{k+1}(1)$

represents the new system state after the k-th iteration. We have

$$s_{k+1}(1) = f(s_k(1), c_k(1)),$$

where function f is defined by the truth table in Supplementary Table 5.

In the manuscript's Fig. 3(b), the Boolean function of 4-bit input and 2-bit output are described. Let $s_k = (s_k(1), s_k(2))$ be the system state at the k-th iteration, where $s_k(1)$ and $s_k(2)$ represent whether *N* and *M* are blocked by *N*' and *M*', respectively. Let $c_k=(c_k(1), c_k(2))$ be the controller at the k-th iteration, where $c_k(1)$ and $c_k(2)$ represent whether the blockers *N*' and *M*' are added at an excess amount, respectively. Then $s_{k+1} = (s_{k+1}(1), s_{k+1}(2))$ represent the system state after the k-th iteration. Since the dynamics at the first 1-bit system state $s_k(1)$ and the second 1-bit system state $s_k(2)$ are independently controlled by $c_k(1)$ and $c_k(2)$, respectively. We have

$$s_{k+1} = (f_1(s_k(1), c_k(1)), f_2(s_k(2), c_k(2))),$$

where function f_1 and f_2 are defined by the truth table in Supplementary Table 6. We can see that f_1 and f_2 are the same as the aforementioned f. So we have

$$s_{k+1} = (f(s_k(1), c_k(1)), f(s_k(2), c_k(2))).$$

This example shows that we may use the Boolean function formulation presented in this work to describe internal structures of the function, such as iterations and compositions.

Supplementary Note 12. Reactions for tetromino assembly

The preformed origami cuboids I, II, III and IV after purification were mixed with certain combinations of blocker sets at an excess amount in 1× TAE buffer supplemented with 35 mM $MgCl_2$. Samples were subjected to an isothermal incubation (42°C for 14 hours) before native agarose gel electrophoresis.

Supplementary Note 13. The MIMO Boolean functions of a quadruple-unit system of origami cuboids

The tetromino assembly is a Boolean function of 16-bit input and 8-bit output. Among the 16-bit input in this quadruple-unit system, the first 8 bits represent whether sticky faces are being blocked, and the rest 8 bits represent whether the blockers are added at an excess amount. Then the 8-bit output define the new system state. This Boolean function as f_3, relative to tetromino shapes, is shown in the truth table at Supplementary Table 8. Let $s_k = s_k(i)$, (i = 1, 2, 3, 4, 5, 6, 7 and 8), where $s_k(i)$ represents whether sticky faces (*A*, *B*, *C*, *D*, *E*, *F*, *G* and *H*) are blocked by the blockers (*A*', *B*', *C*', *D*', *E*', *F*', *G*' and *H*'), respectively. Then $s_{k+1} = (s_{k+1}(i), i=1, \ldots, 8)$ represent the new system state after the k-th iteration. So we have

$$s_{k+1} = f_3(s_k, c_k)$$

where for each $i = 1, \ldots, 8$ we have

$$s_{k+1}(i) = (f(s_k(i), c_k(i)))$$

in which function f is defined by the truth table in Supplementary Table 8. The gray-colored rows in Supplementary Table 8 represent ones to be chosen as experimental groups shown in manuscript's Fig. 5.

Supplementary Note 14. The MIMO Boolean functions of alternative pathways to assemble the J-tetromino

Pathway 1 is a one-pot reaction. The pre-reaction state consists of cuboids I, II, III and IV (sticky faces *A*, *B*, *C*, *D*, *E*, *F*, *G* and *H*), and blockers *A*', *C*', *E*', *F*' and *G*' at an excess amount. This defines a Boolean function, denoted as $f_{pathway1}$, which is the same

as f_3, i.e.,

$$s_{k+1} = f_{pathway1}(s_k, c_k) \equiv f_3(s_k, c_k).$$

For simplicity, we denote the J-tetromino state in Supplementary Figure 60 as J. Then we have

$$J = f_{pathway1}(\mathbf{0}, (1,0,1,0,1,1,1,0)),$$

where 0 represents an 8-dimensional Boolean vector with all 0's. The controller input is the same as the one shown in the gray-colored row in Supplementary Table 8.

Pathway 2 is a hierarchical reaction that contains three steps.

In the first step, cuboid I (sticky face *D*), cuboid IV (sticky faces *D**), and blocker *D*' at an excess amount were mixed. A Boolean function f_4 with 2-bit input and 1-bit output is described. We have

$$s_{k+1}(4) = f_4(s_k(4), c_k(4)),$$
$$s_{k+1}(i) = s_k(i),\ i \neq 4,$$

where $f_4(x, c) = x \text{ OR } c$, for $x, c \in \mathbb{B} \equiv \{0, 1\}$.

In the second step, cuboid II (sticky faces *A**, *B** and *E*), dimer I-IV (sticky faces *A*, *B* and *E**) and blockers *A*', *B*' and *E*' at an excess amount were mixed. A Boolean function f_5 of 6-bit input and 3-bit output is described. We have

$$(s_{k+2}(1), s_{k+2}(2), s_{k+2}(5)) = f_5((s_{k+1}(1), s_{k+1}(2), s_{k+1}(5)), (c_{k+1}(1), c_{k+1}(2), c_{k+1}(5))),$$
$$s_{k+2}(i) = s_{k+1}(i),\ i \neq 1, 2, 5,$$

where $f_5(x, c) = x \text{ OR } c$, for $x, c \in \mathbb{B}^3$.

In the third step, cuboid III (sticky faces *G*, *H*, *C** and *F**), trimer I-II-IV (sticky faces *C*, *F*, *G** and *H**) and blockers *C*', *F*', *G*' and *H*' at an excess amount were mixed. A Boolean function f_6 with 8-bit input and 4-bit output is described. We have

$$(s_{k+3}(3), s_{k+3}(6), s_{k+3}(7), s_{k+3}(8)) = f_6((s_{k+2}(3), s_{k+2}(6), s_{k+2}(7), s_{k+2}(8)), (c_{k+2}(3), c_{k+2}(6), c_{k+2}(7), c_{k+2}(8))),$$
$$s_{k+3}(i) = s_{k+2}(i),\ i \neq 3, 6, 7, 8,$$

where $f_6(x, c) = x \text{ OR } c$, for $x, c \in \mathbb{B}^4$.

This three-step process defines the following Boolean function

$$s_{k+3} = f_{pathway2}(s_k, c_k).$$

And we have

$$J = f_{pathway2}(\mathbf{0}, (1,0,1,0,1,1,1,0)).$$

Pathway 3 is a hierarchical reaction that contains three steps.

In the first step, cuboid I (sticky face *D*), cuboid IV (sticky faces *D**) and the *D*' at an excess amount were mixed. A Boolean function f_4 with 2-bit input and 1-bit output is the same as the first step of pathway 2.

In the second step, cuboid III (sticky faces *G*, *H* and *C**), dimer I-IV (sticky faces *C*, *G** and *H**) and blockers *C*', *G*' and *H*' at an excess amount were mixed. A Boolean function f_7 with 6-bit input and 3-bit output is described. We have

$$(s_{k+2}(3), s_{k+2}(7), s_{k+2}(8)) = f_7((s_{k+1}(3), s_{k+1}(7), s_{k+1}(8)), (c_{k+1}(3), c_{k+1}(7), c_{k+1}(8))),$$
$$s_{k+2}(i) = s_{k+1}(i),\ i \neq 3, 7, 8,$$

where $f_7(x, c) = x \text{ OR } c$, for $x, c \in \mathbb{B}^3$.

In the third step, cuboid II (sticky faces *E*, *F*, *A** and *B**), trimer I-III-IV (sticky faces *A*, *B*, *E** and *F**) and blockers *A*', *B*', *E*' and *F*' at an excess amount were mixed. A Boolean function f_8 with 8-bit input and 4-bit output is described. We have

$$(s_{k+3}(1), s_{k+3}(2), s_{k+3}(5), s_{k+3}(6)) = f_8((s_{k+2}(1), s_{k+2}(2), s_{k+2}(5), s_{k+2}(6)), (c_{k+2}(1), c_{k+2}(2), c_{k+2}(5), c_{k+2}(6))),$$
$$s_{k+3}(i) = s_{k+2}(i),\ i \neq 1, 2, 5, 6,$$

where $f_8(x, c) = x \text{ OR } c$, for $x, c \in \mathbb{B}^4$.

This three-step process defines the following Boolean function

$$s_{k+3} = f_{pathway3}(s_k, c_k).$$

And we have

$$J = f_{pathway3}(\mathbf{0}, (1,0,1,0,1,1,1,0)).$$

Pathway 4 is a hierarchical reaction that contains two steps.

In the first step, two independent systems simultaneously work. One hand, cuboid I (sticky faces *A* and *B*), cuboid II (sticky faces *A** and *B**) and blockers *A*' and *B*' at an excess amount were mixed. A Boolean function f_9 with 4-bit input and 2-bit output is described. We have

$$(s_{k+1}(1), s_{k+1}(2)) = f_9((s_k(1), s_k(2)), (c_k(1), c_k(2))),$$

where $f_9(x, c) = x \text{ OR } c$, for $x, c \in \mathbb{B}^2$.

Another hand, cuboid III (sticky faces *G* and *H*), cuboid IV (sticky faces *G** and *H**) and blockers *G*' and *H*' at an excess amount were mixed. A Boolean function f_{10} with 4-bit input and 2-bit output is described. We have

$$(s_{k+1}(7), s_{k+1}(8)) = f_{10}((s_k(7), s_k(8)), (c_k(7), c_k(8))),$$

where $f_{10}(x, c) = x \text{ OR } c$, for $x, c \in \mathbb{B}^2$. And we have

$$s_{k+1}(i) = s_k(i), i \neq 1, 2, 7, 8.$$

In the second step, dimer I-II (sticky faces *C*, *D*, *E* and *F*), dimer III-IV (sticky faces *C**, *D**, *E** and *F**) and blockers *C*', *D*', *E*' and *F*' at an excess amount were mixed. A Boolean function f_{11} with 8-bit input and 4-bit output is described. We have

$$(s_{k+2}(3), s_{k+2}(4), s_{k+2}(5), s_{k+2}(6)) = f_{11}((s_{k+1}(3), s_{k+1}(4), s_{k+1}(5), s_{k+1}(6)), (c_{k+1}(3), c_{k+1}(4), c_{k+1}(5), c_{k+1}(6))),$$

$$s_{k+2}(i) = s_{k+1}(i), i \neq 3, 4, 5, 6,$$

where $f_{11}(x, c) = x \text{ OR } c$, for $x, c \in \mathbb{B}^4$.

This two-step process defines the following Boolean function

$$s_{k+2} = f_{pathway4}(s_k, c_k).$$

And we have

$J = f_{pathway4}(\mathbf{0}, (1,0,1,0,1,1,1,0))$.

Supplementary References

1. Gillespie, D. T. Exact stochastic simulation of coupled chemical reactions. *J. Phys. Chem.* **81**, 2340-2361 (1977).

2. Lakerveld, R., Stephanopoulos, G. & Barton, P. I. A master-equation approach to simulate kinetic traps during directed self-assembly. *J. Chem. Phys.* **136**, 184109 (2012).

3. Metzler, R. & Ambjörnsson, T. Dynamic approach to DNA breathing. *J. Biol. Phys.* **31**, 339-350 (2005).

4. Ambjörnsson, T., Banik, S. K., Lomholt, M. A. & Metzler, R. Master equation approach to DNA breathing in heteropolymer DNA. *Physical Review E* **75**, 021908 (2007).

5. Ambjörnsson, T., Banik, S. K., Krichevsky, O. & Metzler, R. Breathing dynamics in heteropolymer DNA. *Biophys. J.* **92**, 2674-2684 (2007).

6. Dunn, K. E. *et al*. Guiding the folding pathway of DNA origami. *Nature* **525**, 82-86 (2015).

7. Srinivas, N. *et al*. On the biophysics and kinetics of toehold-mediated DNA strand displacement. *Nucleic Acids Res.* **41**, 10641-10658 (2013).

8. Zhang, D. Y. & Winfree, E. Control of DNA strand displacement kinetics using toehold exchange. *J. Am. Chem. Soc.* **131**, 17303-17314 (2009).

9. SantaLucia Jr, J. & Hicks, D. The thermodynamics of DNA structural motifs. *Annu.Rev.Biophys.Biomol.Struct.* **33**, 415-440 (2004).
