# Supplementary material for: DNA dynamics and computation based on toehold-free strand displacement
Source: Nat Commun. 2021 Aug 17;12:4994. doi: 10.1038/s41467-021-25270-7 (PMC8371076; doi:10.1038/s41467-021-25270-7)
Supplement: Supplementary file 4 — Description of Additional Supplementary Files [file 41467_2021_25270_MOESM4_ESM.pdf]

Title: Supplementary Data 1
Description: DNA sequences

Title: Supplementary Data 2
Description: Codes for simulation models
