# Supplementary material for: DNA dynamics and computation based on toehold-free strand displacement
Source: Nat Commun. 2021 Aug 17;12:4994. doi: 10.1038/s41467-021-25270-7 (PMC8371076; doi:10.1038/s41467-021-25270-7)
Supplement: Supplementary file 5 — Reporting Summary [file 41467_2021_25270_MOESM5_ESM.pdf]

nature research

Corresponding author(s): Qing-Shan Jia, Richard Lakerveld, Bryan Wei

Last updated by author(s): Jul 29, 2021

Reporting Summary

Nature Research wishes to improve the reproducibility of the work that we publish. This form provides structure for consistency and transparency in reporting. For further information on Nature Research policies, see Authors & Referees and the Editorial Policy Checklist .

Statistics

For all statistical analyses, confirm that the following items are present in the figure legend, table legend, main text, or Methods section.

n/a	Confirmed	
☐	☒	The exact sample size (*n*) for each experimental group/condition, given as a discrete number and unit of measurement
☐	☒	A statement on whether measurements were taken from distinct samples or whether the same sample was measured repeatedly
☒	☐	The statistical test(s) used AND whether they are one- or two-sided<br>*Only common tests should be described solely by name; describe more complex techniques in the Methods section.*
☒	☐	A description of all covariates tested
☒	☐	A description of any assumptions or corrections, such as tests of normality and adjustment for multiple comparisons
☐	☒	A full description of the statistical parameters including central tendency (e.g. means) or other basic estimates (e.g. regression coefficient) AND variation (e.g. standard deviation) or associated estimates of uncertainty (e.g. confidence intervals)
☒	☐	For null hypothesis testing, the test statistic (e.g. *F*, *t*, *r*) with confidence intervals, effect sizes, degrees of freedom and *P* value noted<br>*Give P values as exact values whenever suitable.*
☒	☐	For Bayesian analysis, information on the choice of priors and Markov chain Monte Carlo settings
☐	☒	For hierarchical and complex designs, identification of the appropriate level for tests and full reporting of outcomes
☒	☐	Estimates of effect sizes (e.g. Cohen's *d*, Pearson's *r*), indicating how they were calculated

Our web collection on statistics for biologists contains articles on many of the points above.

Software and code

Policy information about availability of computer code

Data collection	AFM images were collected by NANOSCOPE ANALYSIS (version 1.50; Bruker Corp.). TEM images were collected by TEM Imaging & Analysis software (version 4.6.4). The gel images were collected by SynGene_GeneTools software (Version 4.03.05.0) and Amersham Typhoon Control software (version 2.0.0.6). canDNAno software (version 2.0.0) and Uniquimer software (version 1.0) were used to design the DNA nanostructures and produced corresponding DNA sequences.
Data analysis	The fluorescent intensity of gel band was calculated by ImageJ software (version 1.52a) and ImageQuant TL software (version 8.1). Microsoft Excel (version 2016) was used to make graphs. Modeling and simulation were conducted by a custom code in Matlab software (version: R2018b).

For manuscripts utilizing custom algorithms or software that are central to the research but not yet described in published literature, software must be made available to editors/reviewers. We strongly encourage code deposition in a community repository (e.g. GitHub). See the Nature Research guidelines for submitting code & software for further information.

Data

Policy information about availability of data

All manuscripts must include a data availability statement. This statement should provide the following information, where applicable:

- Accession codes, unique identifiers, or web links for publicly available datasets
- A list of figures that have associated raw data
- A description of any restrictions on data availability

The authors confirm that the data supporting the findings of this study are available within the article and its supplementary materials.

Field-specific reporting

Please select the one below that is the best fit for your research. If you are not sure, read the appropriate sections before making your selection.

- [x] Life sciences
- [ ] Behavioural & social sciences
- [ ] Ecological, evolutionary & environmental sciences

For a reference copy of the document with all sections, see nature.com/documents/nr-reporting-summary-flat.pdf

Life sciences study design

All studies must disclose on these points even when the disclosure is negative.

Sample size	No statistical methods were used to predetermine sample size. The data of figures 2 and 3 were independently repeated as technical triplicate and the discrete plots were shown in the bar graphs. In other cases, the data were independently performed at least more than three time that were enough to prove that our DNA nanostructures are correct and rigid, and the dynamic results are correct and credible.
Data exclusions	No data were excluded.
Replication	The data were reliably reproduced using high-quality DNA strands. The data of gel results (agarose and PAGE) and structure images (AFM and TEM) were independently performed more than at least three times. The intervals between repeated experiments are from several hours to several days.
Randomization	Not applicable. All the results collected were subjected to analysis. The repeated tests of certain experiment were performed in the same condition and there was no apparent systematic bias, so randomization is not applicable.
Blinding	Not applicable. The DNA nanostructures were rationally designed and the results are deterministic, so blinding is not applicable.

Reporting for specific materials, systems and methods

We require information from authors about some types of materials, experimental systems and methods used in many studies. Here, indicate whether each material, system or method listed is relevant to your study. If you are not sure if a list item applies to your research, read the appropriate section before selecting a response.

Materials & experimental systems

n/a	Involved in the study	
☒	☐	Antibodies
☒	☐	Eukaryotic cell lines
☒	☐	Palaeontology
☒	☐	Animals and other organisms
☒	☐	Human research participants
☒	☐	Clinical data

Methods

n/a	Involved in the study	
☒	☐	ChIP-seq
☒	☐	Flow cytometry
☒	☐	MRI-based neuroimaging
